# Identification of diverse full-length endogenous betaretroviruses in megabats and microbats

**DOI:** 10.1186/1742-4690-10-35

**Published:** 2013-03-27

**Authors:** Joshua A Hayward, Mary Tachedjian, Jie Cui, Hume Field, Edward C Holmes, Lin-Fa Wang, Gilda Tachedjian

**Affiliations:** 1 Retroviral Biology and Antivirals Laboratory, Centre for Virology, Burnet Institute, Melbourne, VIC, 3004, Australia; 2 Department of Microbiology, Monash University, Clayton, VIC, 3800, Australia; 3 CSIRO Animal, Food and Health Sciences, Australian Animal Health Laboratory, Geelong, VIC, 3220, Australia; 4 Sydney Emerging Infections and Biosecurity Institute, School of Biological Sciences and Sydney Medical School, The University of Sydney, Sydney, NSW, 2006, Australia; 5 Queensland Centre for Emerging Infectious Diseases, Department of Agriculture, Fisheries, and Forestry, Brisbane, QLD, 4007, Australia; 6 Fogarty International Center, National Institutes of Health, Bethesda, MD, 20892, USA; 7 Emerging Infectious Disease Program, Duke-NUS Graduate Medical School, Singapore, Singapore; 8 Department of Microbiology and Immunology, The University of Melbourne, Parkville, VIC, 3010, Australia; 9 Department of Medicine, Monash University, Melbourne, VIC, 3004, Australia

**Keywords:** Retrovirus, Betaretrovirus, Endogenous, Evolution, Bats, *Pteropus*, *Myotis*, *Rhinolophus*

## Abstract

**Background:**

Betaretroviruses infect a wide range of species including primates, rodents, ruminants, and marsupials. They exist in both endogenous and exogenous forms and are implicated in animal diseases such as lung cancer in sheep, and in human disease, with members of the human endogenous retrovirus-K (HERV-K) group of endogenous betaretroviruses (βERVs) associated with human cancers and autoimmune diseases. To improve our understanding of betaretroviruses in an evolutionarily distinct host species, we characterized βERVs present in the genomes and transcriptomes of mega- and microbats, which are an important reservoir of emerging viruses.

**Results:**

A diverse range of full-length βERVs were discovered in mega- and microbat genomes and transcriptomes including the first identified intact endogenous retrovirus in a bat. Our analysis revealed that the genus *Betaretrovirus* can be divided into eight distinct sub-groups with evidence of cross-species transmission. Betaretroviruses are revealed to be a complex retrovirus group, within which one sub-group has evolved from complex to simple genomic organization through the acquisition of an *env* gene from the genus *Gammaretrovirus*. Molecular dating suggests that bats have contended with betaretroviral infections for over 30 million years.

**Conclusions:**

Our study reveals that a diverse range of betaretroviruses have circulated in bats for most of their evolutionary history, and cluster with extant betaretroviruses of divergent mammalian lineages suggesting that their distribution may be largely unrestricted by host species barriers. The presence of βERVs with the ability to transcribe active viral elements in a major animal reservoir for viral pathogens has potential implications for public health.

## Background

Retroviruses (family Retroviridae) are a diverse and widely distributed family of RNA viruses distinguished by their use of a viral RNA-dependent DNA polymerase (reverse transcriptase; RT) and ability to integrate into the genomes of their cellular hosts [1]. In addition to the existence of infectious viral particles that are horizontally transmitted between hosts (exogenous retroviruses), the capacity of retroviruses to integrate into the host germline also generates vertically transmissible endogenous retroviruses (ERVs) [1,2]. ERVs may or may not be capable of producing infectious viral particles, and germline integration over the course of multiple generations typically leads to the accumulation of mutations that render them defective and non-functional [2].

The retroviral family is composed of seven genera: *Alpharetrovirus*, *Betaretrovirus*, *Gammaretrovirus*, *Deltaretrovirus*, *Epsilonretrovirus*, *Lentivirus*, and *Spumavirus*[3]. The genomic organization of retroviruses is classified as either ‘simple’ or ‘complex’, with simple retroviruses encoding the structural polyproteins Gag and Env, and the functional polyproteins Pro and Pol [4]. Complex retroviruses encode additional accessory and regulatory proteins with diverse functions that typically establish and maintain virus replication and pathogenesis [5]. The core elements of all retroviruses are flanked by a pair of typically untranslated nucleotide regions at their 5′ and 3′ ends. In the provirus, formed by integration of the viral cDNA into the host cell chromosome, these regions are referred to as ‘long terminal repeats’ (LTR) [4].

Exogenous retroviruses of zoonotic origin have been associated with disease in humans, the most notable being human immunodeficiency virus (HIV) [6]. Other retroviruses such as human foamy virus (HFV) and human T-cell leukemia virus (HTLV) are known to be capable of infecting humans [7,8]. The retroviruses most recently associated with human disease are betaretroviruses. The up-regulation of gene products derived from the human endogenous retrovirus-K (HERV-K) group of betaretroviruses has been linked to a diverse range of cancers such as those of the breast, ovaries, and prostate alongside other significant human maladies [9,10].

The genus *Betaretrovirus* consists of the Type B and Type D groups of exogenous and endogenous retroviruses and the HERV-K group of endogenous retroviruses. Among the exogenous, infectious members of the genus are the Type B Mouse mammary tumour virus (MMTV), the Type D Jaagsiekte sheep retrovirus (JSRV), which causes pulmonary carcinoma in sheep, and the Type D Mason-Pfizer monkey virus (MPMV) which causes wasting and immunosuppression in new-born Rhesus monkeys [11-13]. All betaretroviruses utilize variants of the lysine tRNA primer binding site (PBS) and encode a deoxyuridine triphosphatase (dUTPase), within their *pro* gene which functions as a nucleocapsid-dUTPase fusion protein [14-16]. Type B and Type D betaretroviruses differ in several respects including their complement of accessory factors, virion morphology, strategies for RNA nuclear export, and the length of their LTR regions. Type B betaretroviruses contain spherical viral cores and have LTRs of ~1,200 nucleotides while Type D contain cylindrical viral cores and have LTRs of ~300 nucleotides. The prototypical Type B betaretrovirus, MMTV, encodes the accessory proteins regulator of export of MMTV mRNA (Rem) and negative acting factor (Naf), which have roles in viral mRNA export, protein synthesis and gene expression [17-19], in addition to the virulence factor, superantigen (Sag) [20]. The Type D retrovirus JSRV has been shown to encode the *trans*-acting factor Rej which has a role in protein synthesis and may assist RNA nuclear export [21]. While no distinct oncogenes or Sag-like virulence-associated proteins are known to be encoded by Type D betaretroviruses, the Env protein of JSRV is associated with oncogenesis [13,14].

There are two major strategies employed by betaretroviruses to export unspliced or partially spliced viral RNA from the nucleus that use distinct export pathways. Complex betaretroviruses such as MMTV employ a HIV Rev-like accessory protein encoded within the *env* gene that binds and facilitates export of intron containing retroviral RNA by recruitment of the cellular karyopherin export factor, chromosome region maintenance 1/exportin 1 (Crm1/Xpo1) [17,19]. Simple betaretroviruses such as MPMV contain a constitutive transport element (CTE) within the nucleotide sequence at the 3′ end of the retroviral genome that recruits a cellular binding factor, Tap (nuclear RNA export factor 1; NXF1) which mediates nuclear export [22,23].

Importantly, ERVs provide a unique opportunity to study the evolutionary history of this family of viruses as they are essentially genetic ‘fossils’ of past retroviral infections [2,24]. As such, their existence serves as an indication of the potential host range of a given retroviral lineage and may be interpreted as evidence for the possible existence of exogenous retroviruses that have yet to be isolated. Indeed, previous studies have reported a number of endogenous betaretroviruses (βERVs) in species for which no exogenous betaretrovirus has yet been identified. These include mammalian species as diverse as primates, horses, rats, lemurs, and an Australian marsupial, the common brushtail possum [25-27].

There are over 1,100 known species of bats (order Chiroptera), accounting for approximately 20% of all mammalian species [28]. Bats are relatively divergent from other mammals, having branched off from the *Perissodactyla* (containing horses) approximately 88 million years ago (mya) [29]. They are divided into two major groups: megabats (suborder Megachiroptera) which are mainly fruit-eating, and microbats (suborder Microchiroptera), small insectivores that navigate by means of echolocation [30]. Notably, bats harbour over 100 viral species from a diverse range of virus families including the *Paramyxoviridae*, *Coronaviridae*, *Herpesviridae*, *Rhabdoviridae*, *Arenaviridae*, *Togaviridae*, *Flaviviridae*, *Orthomyxoviridae*, *Reoviridae*, *Bunyaviridae*, *Filoviridae*, and *Picornaviridae*[31]. Bats, belonging to the mammalian superorder Laurasiatheria, are a major viral reservoir that is evolutionarily distinct from another major viral reservoir, rodents, which together with primates belong to the superorder Euarchontoglires [29,32].

Bats have recently gained attention as they have been implicated in numerous newly emerging diseases of humans caused by viruses such as SARS-coronavirus, Hendra virus, Nipah virus, and the Ebola virus [33-35]. This track record of zoonotic transmission of previously unknown viral pathogens from bats to humans has prompted calls for a proactive approach to future emerging diseases originating in bats [30]. To this end a natural history survey of bats has begun, and we have recently reported the discovery of diversified defective endogenous gammaretroviruses in both mega- and microbats [36,37].

Previous studies of βERVs have tended to focus on isolated viruses, although a report on the βERVs of murid hosts indicated that the genus *Betaretrovirus* might possess a diverse and previously unrecognized range of sub-types extending beyond the classical Type B/Type D paradigm [25]. Using transcriptome and genome analyses of the megabats *Pteropus alecto* (black flying fox) and *Pteropus vampyrus* (large flying fox), and the microbats *Myotis lucifugus* (little brown bat), *Rhinolophus megaphyllus* (eastern horseshoe bat), and *Rhinolophus ferrumequinum* (greater horseshoe bat), we herein examine βERVs present in a diverse range of bat species. In conjunction with phylogenetic analyses, we incorporated the diversity of genomic organizations and the use of specific lysine tRNA PBS to identify eight distinct groups of betaretroviruses.

## Results

### βERVs in bat transcriptomes

To determine if bats contained and expressed a full suite of integrated endogenous betaretroviral genes we generated and analyzed transcriptome databases of *P. alecto*, *R. megaphyllus*, and *R. ferrumequinum*. Gag, Pol, and Env protein sequences were translated from the genomes of extant betaretroviruses: MMTV, JSRV, MPMV, squirrel monkey retrovirus (SMR), and simian retrovirus (SRV). Local tBLASTn searches were conducted to determine if the transcriptomes contained nucleotide sequences that, when translated into any of their six reading frames, contained significant protein sequence similarity to the betaretoviral protein query sequences. Because the variation in length between different transcripts causes difficulty when interpreting relatedness if similarity is expressed as a percentage identity, the significance of the similarity levels observed was determined on the basis of the e-value (probability of random sequence identity) of the BLAST hits. Each transcriptome was found to contain mRNA sequences with notable similarity (e-values < 1×10^-10^) to the betaretroviral proteins Gag, Pol, and Env, with the exception of the *R. ferrumequinum* transcriptome in which no betaretroviral *gag*-like transcripts were identified (Table 1).

**Table 1:** Betaretroviral elements in mega- and microbat transcriptomes

			***P. alecto***		***R. megaphyllus***		***R. ferrumequinum***	
			**Lowest **^**a**^	**# Hits **^**b**^	**Lowest**	**# Hits**	**Lowest**	**# Hits**
			**e-value**		**e-value**		**e-value**	
Betaretroviruses	**JSRV**	Gag	1.65×10^-121^	150	3.86×10^-28^	9	ND	0
		Pol	<1×10^-250^	246	7.80×10^-36^	16	1.0×10^-58^	1
		Env	1.29×10^-51^	48	4.00×10^-15^	3	ND	0
	**SMR**	Gag	1.75×10^-59^	185	1.31×10^-15^	3	ND	0
		Pol	<1×10^-250^	241	2.13×10^-40^	5	7.0×10^-56^	1
		Env	2.58×10^-31^	137	2.65×10^-31^	2	1.0×10^-87^	1
	**MPMV**	Gag	2.16×10^-104^	190	1.49×10^-20^	5	ND	0
		Pol	<1×10^-250^	287	7.71×10^-34^	21	1.0×10^-58^	1
		Env	1.83×10^-33^	140	1.48×10^-31^	6	2.0×10^-98^	1
	**MMTV**	Gag	6.85×10^-53^	90	4.82×10^-13^	2	ND	0
		Pol	<1×10^-250^	269	1.36×10^-31^	15	2.0×10^-45^	1
		Env	8.39×10^-54^	19	ND	0	ND	0
	**SRV**	Gag	1.70×10^-108^	185	2.96×10^-20^	5	ND	0
		Pol	<1×10^-250^	290	9.34×10^-39^	21	9.0×10^-62^	1
		Env	1.31×10^-35^	136	2.75×10^-26^	2	1.0×10^-109^	1

^a^Gag, Pol, and Env proteins were translated from the genomes of extant betaretroviruses and used as search queries in a tBLASTn analysis of the Illumina sequenced transcriptome of *P. alecto*, and the 454 sequenced transcriptomes of *R. megaphyllus*, and *R. ferrumequinum*. The e-value of the transcriptome hit with the greatest sequence similarity (lowest e-value) to each query sequence is displayed.

^b^The number of transcripts identified in the transcriptomes with an e-value < 1 × 10^-10^. ND: No data.

**Figure 1:**
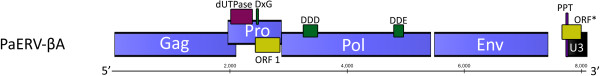
**A schematic representation of PaERV-βA.** Two transcripts were identified in the *P. alecto* Illumina sequenced transcriptome that overlapped by 3,152 nt with 100% sequence identity which were used to assemble the PaERV-βA genomic sequence. Indicated are the retroviral genes *gag*, *pro*, *pol*, and *env*, which have been rendered defective by random mutation since integration. Also shown are the key enzymatic active sites of the viral protease (D×G), reverse transcriptase (DDD), and integrase (DDE); the betaretroviral dUTPase domain in *pro*; two unique open reading frames (ORFs); the polypurine tract (PPT); and the (Unique 3') (U3) region. ORF* does not appear to be genuine, but rather has arisen as a result of an insertion mutation that has disrupted a stop codon.

Reciprocal BLASTx searches of the transcript hits with the lowest e-values (i.e. the top hits presented in Table 1) against the NCBI non-redundant protein database returned predominantly betaretroviral hits. The majority of the mRNA sequences identified within the bat transcriptomes were partial, not being of sufficient length to reveal an entire *gag*, *pol*, or *env* gene sequence. As a point of reference, the nucleotide sequence lengths of MPMV *gag*, *pol*, and *env* are 1,974, 2,583, and 1,758, respectively, while the majority of the transcripts identified in the BLAST analyses were <1,000. The *P. alecto* transcriptome was found to contain two retroviral transcripts 5,433 and 5,830 nucleotides in length which overlap each other by 3,152 bases with 100% sequence identity. The extent of overlap and perfect identity indicated that the two sequences likely represented a full-length retroviral genomic sequence >8,103 bases in length that was later determined through phylogenetic analysis to be a βERV. This full-length *P. alecto* βERV genomic transcript was named PaERV-βA (***P****teropus****a****lecto***E**ndogenous **R**etro**v**irus – **beta**retrovirus **A**) (Figure 1). In addition to PaERV-βA, different transcripts covering the length of one distinct betaretroviral *pol* transcript (PaPol-01) most closely related to JSRV *pol* and one *env* transcript (PaEnv-01) similar to Type C gammaretrovirus and MPMV-like Type D betaretrovirus *env* were identified in the *P. alecto* transcriptome. A single transcript (RfEnv-01) covering the length of a gammaretrovirus-like *env* gene most similar to RD114 *env* was identified in the *R. ferrumequinum* transcriptome. These transcripts were incorporated into the subsequent phylogenetic analyses.

The PaERV-βA sequence was found to begin 25 nucleotides upstream of the *gag* start methionine and contains all of the expected core retroviral genes along with the betaretroviral dUTPase domain (Figure 1). All of the genes were found to be defective as they each contained frameshift mutations. In addition, the *pol* and *env* genes contained premature stop codon mutations. Identification of a 19 nucleotide polypurine tract (PPT) allowed the delineation of the beginning of the unique 3′ (U3) region. Conserved retroviral active site motifs were present in the protease (DxG), reverse transcriptase (DDD), and integrase (DDE) domains. The major homology region (MHR; nucleotide coordinates 1,456 – 1,496) and zinc fingers (nucleotide coordinates 1,752 – 1,805 and 1,866 – 1,919) conserved in *gag* were also present.

Two additional ORFs were identified; the first overlaps the 3′ end of the *pro* gene while the second overlaps the U3 region. However, protein translations of the ORFs compared to the publicly accessible protein family (Pfam) database revealed no known protein domains. In addition, BLASTp analysis of the translations against the NCBI non-redundant protein database yielded no hits. Later identification of closely related *P. vampyrus* βERVs (PvERV-βJ and PvERV-βK) indicated that the ORF overlapping the U3 region was not legitimate.

### βERVs in bat genomes

Given the successful identification of betaretrovirus-like nucleotide sequences in the transcriptomes, we sought to mine the publicly available genomes of *P. vampyrus* and *M. lucifugus* for full-length endogenous betaretroviruses. The aforementioned extant betaretoviral protein sequences together with the retroviral mRNA sequences identified in the bat transcriptomes were used to conduct tBLASTn and tBLASTx searches on the *P. vampyrus* and *M. lucifugus* genomes. These searches revealed a number of hits in the genomes that contained betaretroviral *gag*, *pol*, and *env* genes. Full-length ERVs were delineated by the identification of retroviral *gag*, *pol*, and *env* sequences positioned next to each other and located between a pair of LTRs.

In total, we identified 11 full-length βERVs in *P. vampyrus* and six in *M. lucifugus* (Table 2). These bat βERVs contain all of the expected core elements and the betaretrovirus-specific dUTPase domain. As retroviruses were previously categorized based on the specific tRNA that anneals to their PBS required for initiation of reverse transcription, we determined the specific tRNA used by all identified bat βERVs through nucleotide alignment with known mammalian lysine tRNA sequences (Additional file 1: Figure S1). The PBS was intact and could be identified in the majority of the bat βERVs, and all but one (MlERV-βE) was found to harbour a PBS complementary to either tRNA lysine 1,2 (Lys 1,2) or tRNA lysine 3 (Lys 3) typical of betaretroviruses. Reciprocal BLASTp searches confirmed that the Gag, Pol, and Env of these full-length ERVs were more similar to known betaretroviral proteins than those of other retroviral genera with Pol sequence similarities ranging from 64% to 76% (Additional file 2: Table S1).

**Table 2:** **Full-length endogenous betaretroviruses identified in the Illumina sequenced transcriptome of *P. alecto *and the Sanger sequenced genomes of *P. vampyrus *and *M. lucifugus***

							**Extra**	**LTR**		
		**Genome**	***gag***^***b***^	***pro***^***c***^	***pol***	***env***	**ORFs**^**d**^	**Length**^**e**^	**PBS**^**f**^	**Additional notes**
		**Size**^**a **^**(nt)**					**≥ 300 nt**	**(nt)**		
***P. vampyrus***										
	**PvERV-βA**	7,705	Defective	Defective	Defective	Defective	0	407*	Unknown	100 nt NSR overlapping 5' LTR and beginning of *gag* gene
	**PvERV-βB**	9,257	Defective	**Intact**	**Intact**	Defective	1	1265	Lys 3	102 nt NSR within *gag* gene
	**PvERV-βC**	7,126	Defective	Defective	Defective	**Intact**	0	366*	Lys 3	Short *env* gene may indicate in-frame deletion
	**PvERV-βD**	7,928	Defective	Defective	Defective	Defective	1	398	Lys 1,2	NSRs overlapping 5' LTR and *pro-pol* junction
	**PvERV-βE**	7,879	**Intact**	Defective	**Intact**	**Intact**	1	371*	Lys 3	A single stop mutation in *pro* prevents this ERV being intact
	**PvERV-βF**	7,804	**Intact**	Defective	Defective	Defective	1	370	Lys 3	41 nt NSR at extreme 5' end of the 5′ LTR
	**PvERV-βG**	7,631	Defective	**Intact**	Defective	Defective	0	387*	Lys 3	Appears to contain a deletion that overlaps PPT and 3'LTR
	**PvERV-βH**	7,843	Defective	**Intact**	Defective	Defective	1	361	Lys 3	
	**PvERV-βI**	7,809	Defective	Defective	Defective	Defective	0	371*	Lys 3	
	**PvERV-βJ**	8,773	Defective	**Intact**	**Intact**	**Intact**	2	427*	Lys 1,2	
	**PvERV-βK**	8,611	Defective	Defective	**Intact**	**Intact**	1	425*	Lys 1,2	3' LTR appears truncated
***P. alecto***										
	**PaERV-βA**	>8,103^§^	Defective	Defective	Defective	Defective	2	Unknown	Unknown	Contains artifact ORF (denoted as ORF* in Figure 1)
***M. lucifugus***										
	**MlERV-βA**	9,866	Defective	**Intact**	Defective	Defective	0	422*	Lys 1,2	Large foreign insertion in 5' LTR
	**MlERV-βB**	8,121	Unknown	Defective	Intact	Defective	0	480	Lys 3	669 nt NSR within *gag* gene
	**MlERV-βC**	8,102	**Intact**	**Intact**	**Intact**	**Intact**	0	479*	Lys 3	Completely intact
	**MlERV-βD**	9,007	Defective	Defective	Defective	Intact	0	479*	Lys 3	Contains short foreign insertions in *pro* and *pol* genes
	**MlERV-βE**	7,890	Defective	Defective	Defective	Defective	1	440	Lys^†^	
	**MlERV-βF**	8,235	**Intact**	**Intact**	Defective	Defective	1	470	Lys 3	Small ~45nt deletion overlapping *pol* and *env* genes

^a^ The genome size is given for the proviral version of the βERVs. ^§^ The genome size of PaERV-βA is uncertain as the known sequence begins 25nt upstream of the *gag* gene and does not include the (unique 5') region.

^b^ The core retroviral genes *gag*, *pro*, *pol*, and *env* that contain frameshift or premature stop mutations are described as ‘defective’, those that contain neither of these are described as ‘intact’ in bold font.

^c^ The *pro* open reading frame (ORF) of each βERV was found to encode a betaretroviral dUTPase protein domain.

^d^ The number of ORFs that do not code for the core genes and are 300 nucleotides or greater in length.

^e^ The length of the long terminal repeats (LTRs). * For those βERVs whose 5′ and 3′ LTR lengths differ, the value of the 5′ LTR is given.

^f^ The specific lysine (Lys) tRNA complementary to the primer binding site (PBS) for each βERV is given. ^†^ The specific identity of the PBS of MlERV-βE is uncertain. NSR: non-sequenced region.

All of the bat βERVs possessed LTRs of 300–500 nucleotides in length, as expected for Type D betaretroviruses with the exception of PvERV-βB with LTR length typical of Type B betaretrovirues (1265 bp) (Table 2). Each bat βERV was found to contain a PPT immediately upstream of their 3′ LTR regions. We analyzed each *pro* and *pol* gene and identified the expected enzymatic active site motifs in the retroviral protease (D×G), reverse transcriptase (DDD), and integrase (DDE) domains. The *gag* gene of each βERV contained the expected MHR and zinc-knuckles. While the *M. lucifugus* genome sequencing coverage was relatively high (7× coverage), the *P. vampyrus* genome has only been sequenced to 2.6x coverage. The nature of a low-coverage genome such as this means that within the assembled ‘scaffolds’ there occasionally exist stretches of nucleotides of ambiguous identity. In this regard, several of the bat βERVs reported herein contain short ‘non-sequenced regions’ (NSR) (Table 2). As a result, the PBS present in PvERV-βA and the MHR of PvERV-βB could not be identified as they contained NSRs overlapping those elements. To confirm that each βERV was the product of a retroviral integration event, the four-nucleotide repeats known as genomic target site duplication (TSD) sequences that flank the proviruses were identified (Additional file 2: Table S2). TSDs were identified for all proviral βERVs with the exception of PvERV-βD and F whose 5′ LTRs were masked by NSR, PvERV-βK whose 3′ LTR appears to be truncated, and PvERV-βB which is the sole βERV to have intact and unambiguous LTRs yet no identifiable TSDs. To determine if closely related clusters of βERVs were generated as a result of post-integration chromosomal duplication events, we compared their flanking chromosomal DNA through a BLASTn analysis (Additional file 2: Table S3). One pair of bat βERVs (PvERV-βK and PvERV-βJ) was found to have homology in the chromosomal regions immediately up- and downstream of the proviruses. PvERV-βK and PvERV-βJ appear to have arisen as a result of a duplication of a single integrated provirus. The truncation of the 3′ LTR of PvERV-βK suggests a chromosomal duplication event.

### Phylogenetic analysis of betaretroviral Gag, Pol and Env elements

**Figure 2:**
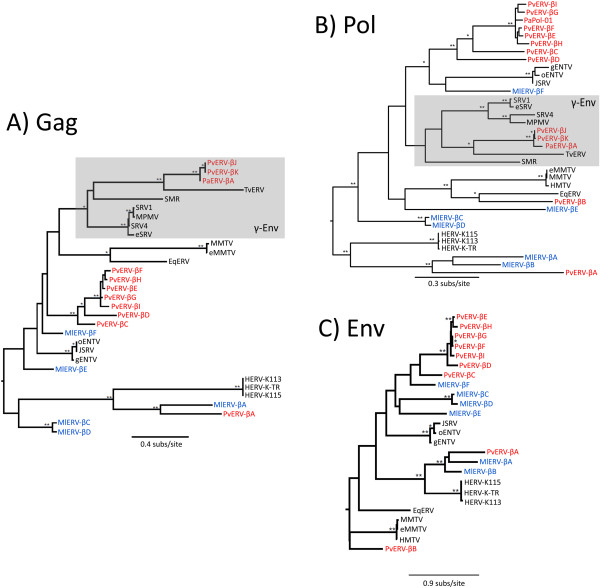
**Phylogenetic relationships of bat and non-bat betaretroviruses.** Maximum likelihood phylogenetic trees of (**A**) Gag, (**B**) Pol, and (**C**) Env amino acid sequences. Bootstrap values <70% are not shown, and branch lengths are drawn to a scale of amino acid substitutions per site. Bootstrap values are denoted as ** >90%; * >70% and < 90%. The trees are midpoint rooted for purposes of clarity only. βERV proteins of *P. vampyrus* and *P. alecto* are highlighted in red text. βERVs of *M. lucifugus* are highlighted in blue text. The clades within the Gag and Pol trees highlighted with a grey background (γ-Env) contain betaretroviruses whose Env sequence is not sufficiently closely related to the Env of other betaretroviruses to be included in the Env tree.

Next, we examined the phylogenetic relationships of the bat βERVs identified in our analysis of the bat genomes and transcriptomes (Table 2). Accordingly, the Gag, Pol, and Env of the full-length bat βERVs were aligned with those of known exogenous and endogenous betaretroviruses and phylogenetic trees were estimated for each (Figure 2).

In all three trees a great diversity of bat βERVs was observed, with individual βERVs clustering with members of the Type D (e.g. MPMV and JSRV), Type B (e.g. MMTV), and HERV-K groups. The close relationship between viral sequences derived from transcriptomes and some endogenous viral sequences mined from bat genomes suggests that at least some of the bat βERVs have the ability to transcribe. Notably, a number of bat βERVs (PvERV-βJ, K and PaERV-βA), together with several exogenous betaretroviruses, were found to possess Env sequences that formed a cluster so highly divergent, and more closely related to gammaretroviruses, as to require omission from the initial betaretroviral Env tree (Figure 2C). Finally, we also found some evidence for within-genome recombination (e.g. MlERV-βC, D and E) as reflected in the phylogenetic incongruence between the Gag and Pol and Env trees.

### Phylogenetic analysis of betaretrovirus and gammaretrovirus Env

**Figure 3:**
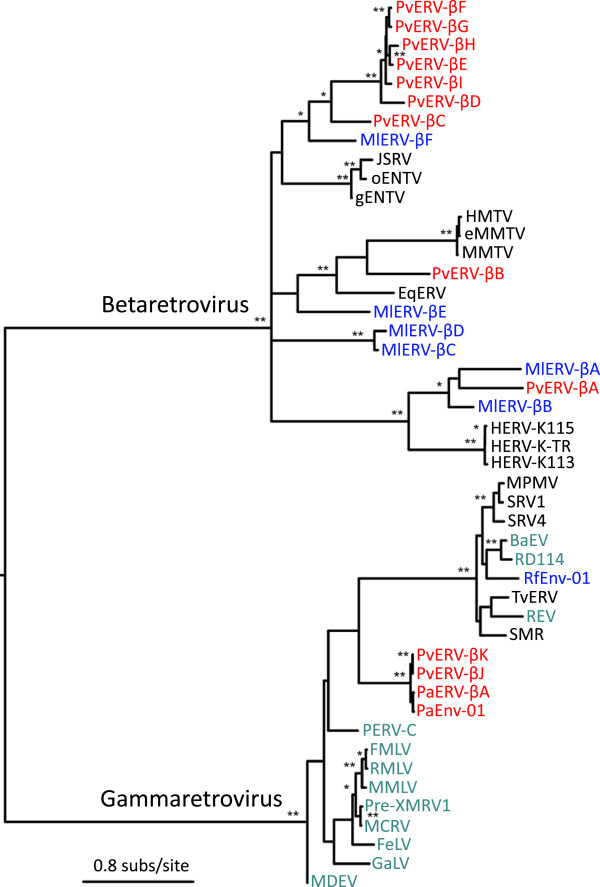
**Phylogenetic comparison of the envelope (Env) protein sequence of betaretroviruses and gammaretroviruses.** Bootstrap values <70% are not shown, and branch lengths are drawn to a scale of amino acid substitutions per site. Bootstrap values are denoted as ** >90%; * >70% and <90%. βERV proteins of *P. vampyrus* and *P. alecto* are highlighted in red text. βERVs of *M. lucifugus* and *R. ferrumequinum* are highlighted in blue text. Gammaretroviruses are highlighted in teal text.

Our reciprocal tBLASTx searches indicated that the *P. alecto* ERV (PaERV-βA) and two of the *P. vampyrus* ERVs (PvERV-βJ and K) encoded Env sequences that were more similar to gammaretroviral Env, while still possessing Gag and Pol sequences that closely resembled those of known betaretroviruses (see above). To confirm this observation we undertook a phylogenetic analysis of the Env sequences of known gammaretroviruses and betaretroviruses, together with the newly identified βERV Env sequences (Figure 3). This analysis confirmed previous observations [12,38] that the Env sequences of some extant Type D betaretroviruses, namely MPMV, SMR and simian retrovirus serotypes 1 and 4 (SRV1 and SRV4), cluster with gammaretroviral Env, as do those of PvERV-βJ, K, PaERV-βA, PaEnv-01 (Env sequence derived from *P. alecto*), and RfEnv-01 (Env sequence derived from *R. ferrumequinum*). Other Type D retroviruses such as JSRV and the enzoonotic nasal tumor viruses (ENTV) of sheep and goats did not fall into this cluster. This indicates that a recombination event has occurred, in which a sub-lineage of Type D betaretroviruses acquired a gammaretroviral *env* gene.

### Analysis of bat βERV sub-genus clades

**Figure 4:**
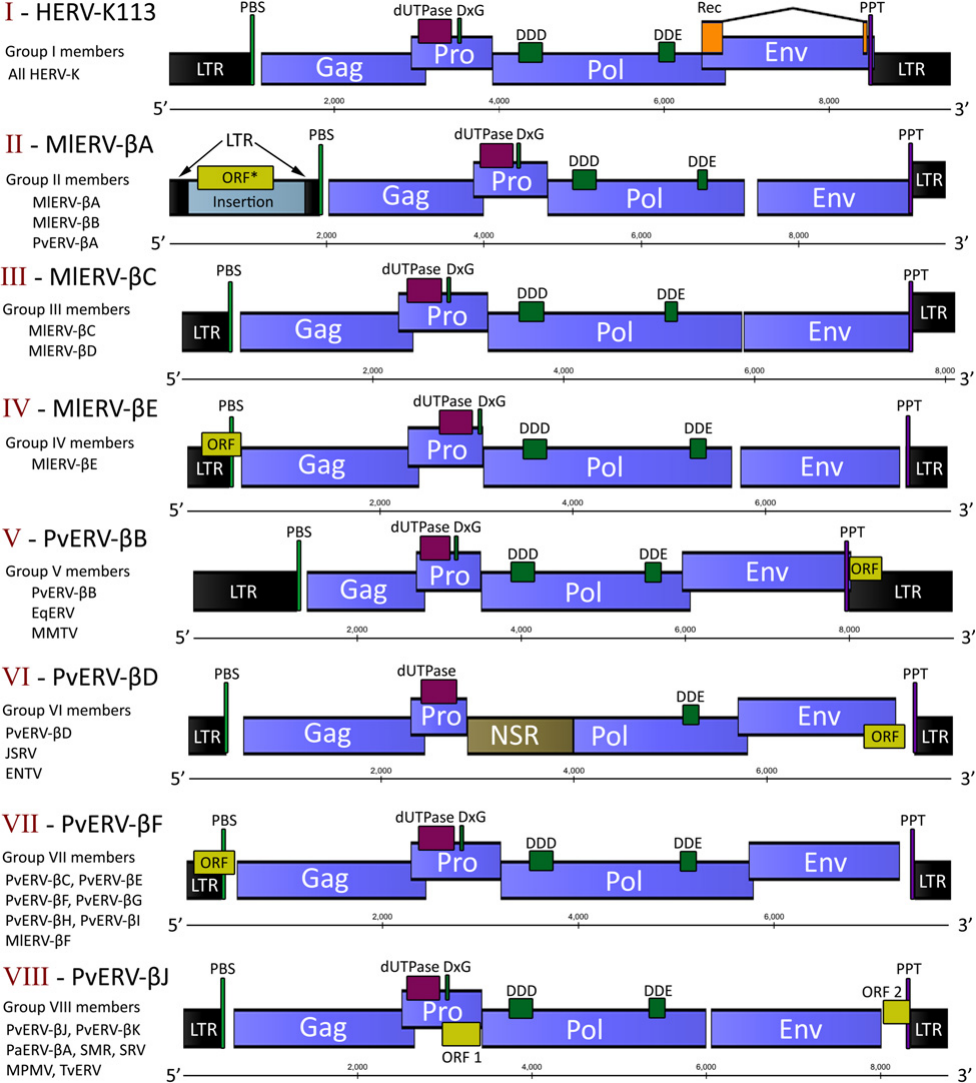
**Eight sub-groups of the *betaretrovirus *genus.** A schematic diagram for a single representative of each group is depicted. Core retroviral genes *gag, pro, pol,* and *env* are bordered by the proviral long terminal repeats (LTRs). Also shown are other major genetic features such as open reading frames (ORFs) greater than 300nt in length and the *rec* gene of HERV-K113, enzymatic active site motifs of protease (D×G), reverse transcriptase (DDD), and integrase (DDE); the primer binding site (PBS) and polypurine tracts (PPT); and the characteristic betaretroviral dUTPase domain. NSR: non-sequenced region. ORF* is part of foreign nucleotide insertion within MlERV-βA and does not appear to be a retroviral element.

Our analysis of the full-length bat βERVs revealed an unexpected diversity of genomic organizations, as a number were found to contain unique ORFs. Some of these ORFs were in alternative reading frames within the core element domains and others were either upstream of *gag*, or downstream of *env*. Furthermore, the differential use of tRNA Lys 1,2 and tRNA Lys 3 was not found to be restricted to either Type B or Type D betaretroviruses. Rather, it appears that a switch between the two has occurred multiple times throughout the history of the genus. This diversity of genomic organization was used in conjunction with the phylogenetic analyses of Gag, Pol, and Env (with prime consideration given to the highly conserved Pol phylogeny) and the tRNA usage to identify eight distinct groups within the *Betaretrovirus* genus (Figure 4). The eight betaretroviral subgroups that we propose are distinguished from each other by major evolutionary differences such as deep phylogenetic divergence with strong bootstrap support (>90% of trees resolving the clade), significant mutations in key genetic features such as a switch to the use of a different PBS, or the presence of retroviral genes from a different genus.

**Group I** (represented by HERV-K113) consists of the HERV-K group of endogenous betaretroviruses which contain a PBS similar to tRNA Lys 1,2 and have a deep phylogenetic divergence from other betaretroviruses. No known exogenous betaretroviruses or bat βERVs currently reside in Group I.

**Group II** (represented by MlERV-βA) consists of a phylogenetic cluster of endogenous bat βERVs that branched off from Group I early in betaretroviral history. Three bat βERVs are included in this group. The PBS of MlERV-βA and MlERV-βB are complementary to tRNA Lys 1,2 and Lys 3, respectively, while the tRNA usage of PvERV-βA is unknown as a 100 nucleotide NSR overlaps its PBS. MlERV-βA contains a large 1,493 nucleotide insertion within its 5′ LTR that contains a 323 codon ORF. This insertion presumably arose post-integration and the nature of this genetic element is unknown. A Pfam domain search and BLASTp analysis of the translation of the ORF against the NCBI non-redundant protein sequence database did not identify any known protein domains or similarity to any known protein.

**Group III** (Represented by MlERV-βC) consists of microbat ERVs that possess a phylogenetically divergent Pol (bootstrap support >90%) and a PBS complementary to tRNA Lys 3. Within this group is MlERV-βC, the first fully intact bat βERV to be identified, and which raises the possibility that exogenous members of Group III may yet exist as undiscovered infectious betaretroviruses.

**Group IV** (Represented by MlERV-βE) appears to have diverged as a part of the Type B betaretroviral lineage. However, the precise phylogenetic position of Group IV’s sole member, MlERV-βE, is not supported by high bootstrap support in any of the trees. Furthermore the precise identity of its PBS is uncertain. The PBS does not appear to be specifically complementary to either Lys 1,2 or Lys 3 tRNA, but rather it appears to be complementary to an alternative mammalian lysine tRNA. There are presently no known extra copies of MlERV-βE within the *M. lucifugus* genome.

MlERV-βE is distinguished by its possession of a unique ORF upstream of Gag. This ORF begins within the 5′ LTR and terminates three nucleotides upstream of the Gag start methionine, within the same reading frame. ORFs upstream of Gag may be relevant to Gag expression considering that murine gammaretroviruses encode an alternative N-terminally extended version of Gag, glyco-gag, that has a role in the promotion of viral replication [39,40]. No promoter elements or TATA boxes were predicted to exist upstream of the ORF, however a TATA box is predicted within the ORF coupled with a possible start methionine downstream, encoding a potential 84 amino acid protein.

**Group V** (Represented by PvERV-βB) consists of archetypically structured Type B betaretroviruses (MMTV-like) that contain long LTRs (~1,200 bases). It is possible that the extension of the 3′ LTR has facilitated the emergence of ORFs in this location as in the case of MMTV’s *sag* gene. In this regard, PvERV-βB has an ORF within its 3′ LTR. This ORF is 123 codons in length, much shorter than MMTV’s Sag protein, which is 320 amino acids long. While it is possible that the ORF was longer at integration and has simply been interrupted by stop codon mutations since that time, a tBLASTn analysis of MMTV’s Sag protein against the 3′ LTR of PvERV-βB did not reveal any significant sequence similarity. Also in this group is EqERV, an endogenous horse betaretrovirus, which does not contain a *sag* gene or *sag*-like ORF within its 3′ LTR [27].

**Group VI** (Represented by PvERV-βD) consists of JSRV-like Type D betaretroviruses that contain short LTRs (~300 bases) and Env protein sequences that do not phylogenetically cluster with those of the *gammaretrovirus* genus. Members of this group harbour a PBS complementary to tRNA Lys 1,2 and may or may not contain additional ORFs within their core element domains, as is the case for JSRV and ENTV’s ORF-x located within *pol*, and PvERV-βD, which has an ORF overlapping the 3′ end of the *env* gene.

**Group VII** (Represented by PvERV-βF) consists wholly of bat βERVs. Group VII members are phylogenetically Type D-like and are primarily distinguished by a PBS complementary to tRNA Lys 3 as opposed to tRNA Lys 1,2 which is the expected PBS complementarity for Type D betaretroviruses. Also, several bat βERVs in this group possess a unique ORF upstream of Gag that is distinct from that of group IV’s MlERV-βE. This ORF begins within the 5′ LTR and terminates 26 nucleotides upstream of the *gag* start codon. Promoter elements and TATA boxes are predicted to exist upstream of this ORF. As there were differences in the start position of this ORF in the various group VII bat βERVs (PvERV-βE - I), likely due to random mutation since integration, a nucleotide alignment of the region was generated (Additional file 1: Figure S2). The alignment demonstrated that the consensus ORF contained a possible start methionine that would code for a 101 amino acid protein. One member of this group, PvERV-βE, is almost fully intact as it does not appear to contain any frameshift mutations and only a single premature stop codon within the *pro* gene.

**Group VIII** (Represented by PvERV-βJ) consists of MPMV-like Type D betaretroviruses. The distinguishing feature of this group is the possession of an encoded Env polyprotein that phylogenetically clusters with those of gammaretroviruses rather than those of other betaretroviruses. The bat βERVs in this group have an additional feature which is an ORF beginning 40 bases downstream of the *env* stop codon and terminating 15 bases into the 3′ LTR. This is exemplified in PvERV-βJ. A nucleotide sequence alignment of the extreme 3′ region (Additional file 1: Figure S3) of the closely related PvERV-βJ, K, and PaERV-βA generated a consensus sequence that contained this ORF and revealed that the equivalent ORF sequences in PvERV-βK and PaERV-βA are respectively interrupted by a frameshifting deletion mutation and stop mutation. This ORF contains a possible start methionine that would generate a 90 amino acid protein. This alignment also indicated that the alternative ORF* in PaERV-βA (Figure 1) was likely to be an artifact as the U3 region contained an eight nucleotide insertion that disrupts a stop codon which, if the insertion did occur after integration, has generated an artificial ORF. The PaERV-βA genome was derived from Illumina based transcriptome sequencing while the PvERV-βJ and PvERV-βK genomes were derived through whole-genome shotgun/Sanger sequencing. Accordingly, each method can be used to orthogonally verify the other. A full alignment of the three proviruses (Additional file 3: Figure S4; demonstrating 96.66% nucleotide identity between PvERV-βJ and PvERV-βK and 93.77% between PvERV-βK and PaERV-βA) supports the veracity of these proviral sequences and provides further evidence that the group VIII βERVs are likely derived from a single integration event.

The unique ORFs identified in the bat βERVs of all groups were subjected to a BLASTp analysis against the NCBI non-redundant protein database and Pfam domain search. However, no BLAST hits or known protein domains were identified.

### Analysis of elements involved in nuclear export of intron-containing bat betaretroviral RNA

To determine if the groupings we had assigned were congruent with known functional differences between retroviruses with respect to betaretroviral RNA nuclear export strategies, we analyzed the bat βERVs, alongside known exogenous and endogenous betaretroviruses, for evidence of motifs indicative of the major export strategies (Additional file 2: Table S4). To this end we employed a computational analysis to search for the presence of nuclear localization signals (NLS) and nuclear export signals (NES) common to the retroviral Rev-like proteins used in the archetypal Rev/Rev-responsive element (RRE) equivalent export mechanism. We also searched for the presence of Tap-binding elements (TBE) within and downstream of the *env* gene, which would imply the utilization of the CTE export pathway, and for direct nucleotide repeats (DR) and inverted nucleotide repeats (IR) that might suggest the formation of stem-hairpin-loop structures known to be associated with the CTE [23].

While a number of βERVs were predicted to contain either an NLS or an NES, only MlERV-βB and PvERV-βB were found to contain both. These βERVs broadly cluster with HERV-K and MMTV, which respectively encode the Rev-like proteins Rec and Rem, and the presence of both NLS and NES points to the possibility that they encode Rev-like proteins and make use of the Crm1 nuclear RNA export pathway. The majority of the βERVs in group VII were found to contain TBE, indicating that the original exogenous forms of these retroviruses likely utilized the nuclear export pathway accessed by the CTE.

### Molecular clock analysis of LTRs

We used an analysis of the LTRs to estimate the time since integration of the bat βERVs. This analysis evaluated the extent of the difference between the nucleotide sequences of the 5′ and 3′ LTRs of each βERV, which are expected to be identical at the time of integration. The number of nucleotide differences between the 5′ and 3′ LTR is assumed to be proportional to the time since integration, although this may be compromised by such factors as gene conversion [41]. Under this assumption, all βERVs integrated into the genomes of the ancestors of modern bats within a wide time range of between 3.2 and 36.3 million years ago (mya), and hence long after the divergence of bats from other mammalian lineages (Table 3). This, in turn, suggests that (i) that the original exogenous forms of these βERVs targeted ancient bats, and (ii) there has been a continual integration of betaretroviruses into bat genomes during their evolutionary history.

**Table 3:** Estimation of time since integration

	**Divergence**^**a**^	**Age (mya)**^**b**^
***P. vampyrus***		
** PvERV-βA**	0.024	30
** PvERV-βB**	0.011	13.8
** PvERV-βC**	0.027	33.8
** PvERV-βD**	0.056	ND
** PvERV-βE**	0.011	13.8
** PvERV-βF**	0.043	ND
** PvERV-βG**	0.039	ND
** PvERV-βH**	0.006	7.5
** PvERV-βI**	0.029	36.3
** PvERV-βJ**	0.005	6.3
** PvERV-βK**	0.025	ND
***M. lucifugus***		
** MlERV-βA**	0.055	29
** MlERV-βB**	0.043	22.6
** MlERV-βC**	0.008	4.2
** MlERV-βD**	0.012	6.3
** MlERV-βE**	0.007	3.7
** MlERV-βF**	0.006	3.2

^a^ 5′ and 3′ LTR divergence: number of differences, per nucleotide, per site.

^b^ Molecular clock dating was used to estimate the time in millions of years (mya) since the integration of each betaretrovirus into the host genome, based on the number of nucleotide differences between the 5′ and 3′ LTRs of each betaretrovirus [25].

ND: Not dated; these βERVs could not be dated using this method. PvERV-βD and PvERV-βF contained non-sequenced regions within their 5′ LTR, while PvERV-βG and PvERV-βK contained bulk deletions within their 3′ LTRs.

### Betaretroviral evolution and diversification

**Figure 5:**
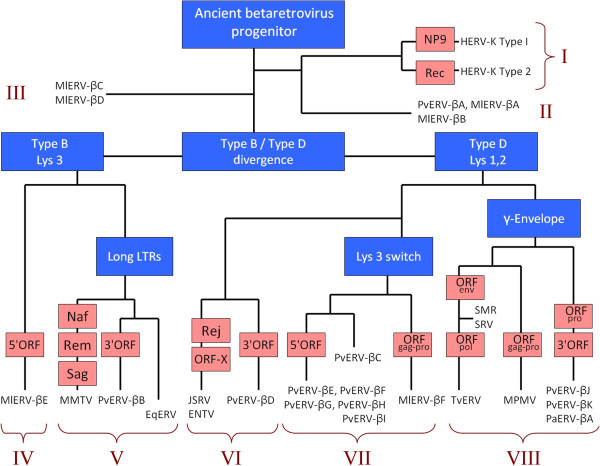
**A proposed series of events leading to the current diversity in the genus *Betaretrovirus.*** The proposed series of evolutionary events leading to eight distinct sub-groups of betaretroviruses based on a combination of the phylogenetic analyses of Gag, Pol, and Env protein sequences, and the genomic features and organizations of individual betaretroviruses.

We coupled our analysis of the genomic features of the bat βERVs with the phylogenetic patterns observed in the Gag, Pol, and Env trees (with primacy given to the phylogeny of the highly conserved polymerase sequences) to generate a hypothetical series of events that may have led to the current state of diversity in the genus *Betaretrovirus* (Figure 5).

Our analysis indicates that while the ancient progenitor betaretrovirus likely made use of a tRNA Lys PBS, its specific identity is uncertain. Groups I and II appear to have branched off together early in betaretroviral history. This has led, in the case of the HERV-K betaretroviruses, to the emergence of distinct genetic elements such as the NP9 and Rec proteins, whose current endogenized forms have possible roles in tumorgenesis [42,43]. Group III’s phylogenetic position places its point of divergence after the split of Groups I and II but prior to the split between the Type B and Type D lineages.

The divergence between Type D and Type B βERVs seems to have occurred as a result of their differential use of tRNA Lys 1,2 and tRNA Lys 3, respectively. Within the Type B lineage are groups IV and V which, although possibly splitting after the divergence of Type B and Type D, differ in the length of their LTRs, their tRNA usage, and their additional genetic elements. Within the Type D lineage an early event appears to have been a recombination between a betaretrovirus and a gammaretrovirus, which has caused a divergence between JSRV-like and MPMV-like Type D betaretroviruses. In this split, group VIII appears to have diverged from groups VI and VII through the acquisition of a gammaretroviral *env* gene. Group VII later diverged from group VI by a switch from the use of tRNA Lys 1,2 to tRNA Lys 3 and differentiation of their additional ORFs.

## Discussion

We searched for the expression of betaretroviral genes in the transcriptomes of the megabat *P. alecto* and the microbats *R. megaphyllus* and *R. ferrumequinum*. Through this analysis we determined that betaretroviral genes were being transcribed into mRNA within each species and we identified that a full-length genomic transcript of a betaretrovirus (PaERV-βA) was being expressed in *P. alecto*. As each of the genes of PaERV-βA were found to contain mutations that likely rendered them non-functional, it seems reasonable to conclude that the transcript was expressed from a defective βERV rather than a functional exogenous betaretrovirus. It is important to note that we cannot exclude the possibility that the reported PaERV-βA transcript was derived from multiple similar sequences during transcriptome assembly and due to recombination between similar transcripts during cDNA synthesis or PCR as published [44].

Our analysis of the genomes of the megabat *P. vampyrus* and the microbat *M. lucifugus* revealed that they contain a genetically diverse range of full-length βERVs. In the case of *M. lucifugus* this included an intact βERV (MlERV-βC) that did not contain any mutations that would clearly render the gene products non-functional. However, it should be noted that as revealed by the LTR analysis, nucleotide substitutions have occurred in the MlERV-βC sequence. While the critical enzymatic active site motifs are intact, whether or not the nucleotide substitutions that have occurred in the coding domains would have a detrimental effect on the functionality of the gene products is not known.

In analyzing the genetic content of the full-length βERVs for the presence of ORFs, aside from those coding for the core genes, we set a minimum cut-off of 100 codons to limit the amount of incidental non-coding ORFs that would be identified. However, many retroviral accessory and regulatory genes, such as *rec* and *np9* of HERV-K and *vpr* and *tat* of HIV-1, are shorter than 100 codons and are often encoded over the span of two exons. Despite the high minimum cut-off, it is striking that the bat βERVs possessed a diverse array of additional ORFs. While we cannot confirm that these are indeed protein coding domains, much less speculate on their function, the existence of similar elements is not without precedent among the betaretroviruses. One example is the ‘ORF x’ of JSRV, the function of which is unknown but it has been found to be broadly conserved amongst JSRV isolates [45]. Several of the ORFs we identified overlap the proviral LTRs, which consist of typically untranslated regions. This is also not unprecedented, with a prime example being the *sag* gene of the betaretrovirus MMTV, which is situated entirely within the U3 region of the 3′ LTR. The presence of unique ORFs in βERVs may indicate the evolution of novel retroviral genes whose products have regulatory or accessory functions required for the retroviral life-cycle and/or pathogenesis. In addition to the βERVs reported in this study we noted the presence in both mega- and microbats of betaretrovirus-like retroelements that resemble βERVs but lack the *env* gene; these were not investigated further (data not shown).

We reported each βERV as a distinct entity. Nevertheless it is reasonable that some of their number, particularly the βERVs within each of groups VII and VIII, represent a common progenitor infectious betaretrovirus that has undergone duplication events via retrotransposition or recombination since an original, single integration event. For example, the integration time of PvERV-βJ coupled with its similarity to PaERV-βA and PvERV-βK may mean that these βERVs originated from a single integration into the genome of the common ancestor of *P. vampyrus* and *P. alecto* and that at least a single duplication event has occurred within *P. vampyrus* (or the common ancestor). However, it is also arguable that multiple integrations of closely related infectious retroviruses separated from each other by perhaps a small number of infectivity cycles occurred. We attempted to address this question by a comparative analysis of the flanking genomic DNA located immediately up- and downstream of the proviruses and by identifying the TSD that border each provirus and arise as a by-product of the integration mechanism [46]. Unique TSD indicate distinct integration events. In the case of the group VIII βERVs a TSD for PvERV-βK could not be identified as its 3′ LTR appears to be truncated. This may indicate that it is a copy of PvERV-βJ that has arisen through a chromosomal duplication event. This appears to be confirmed by the identification of genomic DNA bordering PvERV-βJ that is homologous to genomic DNA flanking PvERV-βK. As PaERV-βA is a genomic transcript it does not contain TSD. In the case of group VII βERVs all of the identifiable TSD differ from one another, indicating separate integration events. Additionally, no flanking genomic DNA homology was identified amongst the members of the group.

Notably, both the phylogenetic and LTR analyses revealed a great diversity of βERVs in bat genomes. Our molecular clock dating suggested that the earliest viral incorporation event occurred at approximately 36 mya which is older than the separation of the megabats and microbats studied (around 20 mya) [28]. In addition, it is clear that some of the βERVs present in bat genomes were vertically transmitted from their ancestors; e.g. MlERV-βA and PvERV-βA are grouped together and are of similar age having been integrated approximately 30 mya. However, it is also the case that many of the bat βERVs formed via independent viral invasion and incorporation as they have different phylogenetic positions as well as different estimated ages of integration.

In addition to their genomic diversity, we observed that a number of phylogenetic clusters within the genus differed in their more fundamental aspects. Specifically, the use of tRNA Lys 1,2 or tRNA Lys 3 was not restricted to the divide between Type B and Type D betaretroviruses, and a clade that was distinct in both Gag and Pol trees possessed a gammaretroviral *env* gene. This prompted us to define eight sub-groups (Group I-VIII) within the genus that accounted for these fundamental differences in the context of phylogenetic divergences at the amino acid level of the core polyproteins. Our LTR analysis also revealed that bats have been infected with betaretroviruses for most of their evolutionary history. This supports the notion that bats are a potential reservoir for infectious betaretroviruses.

A previous study reported a short, partial retroviral sequence (CpERV-β5, AC138156) in the genome of the microbat *Carollia perspicillata* (Seba’s short-tailed bat) [25]. However, this sequence contained large deletions, was missing the entire *pro* and *pol* genes, and only fragments of the *gag* and *env* genes remained. The partial Env of CpERV-β5 most closely matched the Env of the betaretrovirus SMR and on that basis it was reported as a betaretroviral sequence. In this study, we report a series of complete βERVs in mega- and microbat genomes representing the breadth of the genus *Betaretrovirus*. Although CpERV-β5 does contain a lysine tRNA-specific PBS, without a *pol* gene to phylogenetically differentiate it or the presence of the characteristically betaretroviral dUTPase domain within *pro*, it cannot be known with certainty whether it is a group VIII betaretrovirus or a gammaretrovirus. The study by Ballie *et al.*[25] and a recent study by Anai *et al.*[47] both noted the similarity between the Env of Type C gammaretroviruses and some Type D betaretroviruses which was attributed to a likely recombination event. We have shown that the betaretroviruses, which possess a gammaretrovirus-like Env, form a single clade in both Gag and Pol phylogenies. This indicates that a single recombination event produced these group VIII betaretroviruses. Furthermore, the typical mammalian gammaretroviral use of tRNA proline and glycine-specific PBS and the absence of dUTPase domains from their *pro* genes [14] can be used to infer that the nature of the recombination event was the insertion of a Type C gammaretroviral *env* gene into a Type D betaretrovirus. Previous studies also determined a recombinatorial origin for the Type D *env*[12,38]. However, this conclusion was reached prior to the sequencing of the genome of JSRV [48], which does not possess a gammaretrovirus-like Env, and its subsequent classification as a Type D retrovirus. As such, it was hypothesized that it was this recombination event that gave rise to the Type D lineage of betaretroviruses [12,38]. Our analysis aimed to provide a clarification of the differences between, within, and outside of the Type B and D groups of betaretroviruses. Accordingly, we suggest that the fundamental feature giving rise to the division between the Type B and D lineages may have been the use of different primer binding sites, not the possession or not of a Type C *env* gene, which appears to be a more recent and more significant lineage divergence within the Type D group.

Ballie *et al.*[25] described seven groups within the genus *Betaretrovirus*. These groupings were made solely on the basis of *pol* gene nucleotide sequence similarity. While manually determining amino acid sequences from genes that contain frameshift mutations is difficult, when the manual reconstruction is closely informed by the alignment of each translated frame against known betaretroviral polymerases, amino acid sequence reconstruction is a viable option. As such, our phylogenetic analyses differ from those undertaken previously in that they are based on amino acid sequence alignments, and our groupings are based on differences in the fundamental genomic features in addition to phylogenetic clustering. Tristem [49] reported on the identification and classification of the highly diverse endogenous retroviruses present in the human genome (HERVs) and suggested that tRNA PBS specificity, in addition to the polymerase phylogeny of endogenous retroviruses, should inform their classification. This is because even if the ERVs of a given species cluster together in phylogenies, the use of different tRNA PBS may be evidence of separate origins. Indeed, that study made the assumption that HERVs with alternative PBS homologies were derived from cross-species transmissions. With this in mind, we analyzed the PBS sequences of the identified βERVs and used this information to aid and inform the delineation of our grouping scheme.

Mammalian cells restrict the export of intron containing mRNA from the nucleus to the cytoplasm, and betaretroviruses have been found to utilize two different mechanisms to circumvent this restriction and export unspliced genomic RNA and singly-spliced *env* mRNA. The Type B betaretrovirus MMTV, and the HERV-K endogenous retroviruses are known to use Rem and Rec, respectively, which are HIV Rev-like export proteins, that possess equivalent mechanisms of action [17,50-52]. The Type D betaretroviruses MPMV and SRV make use of the *cis*-acting CTE, which in the absence of a retroviral accessory protein, recruits cellular proteins to effect nuclear export of intron containing viral RNA [22,23]. This apparent dichotomy has been complicated by recent lines of investigation that have found that i) MMTV likely possesses a second, Rem-independent mechanism for the export of singly-splice *env* mRNA [52]; and ii) the Type D betaretrovirus JSRV contains both a CTE and a Rev-like protein, Rej, which while found to possess a primary function related to Gag synthesis, also enhances RNA export in some cell types [21,53]. This indicates that betaretroviruses may make use of multiple export mechanisms, possibly providing some measure of redundancy to promote productive replication in different contexts.

We conducted a computational analysis to predict the presence of RNA export motifs that would indicate which mechanism was utilized by each βERV. We found that bat βERVs, clustering with betaretroviruses known to utilize the Crm1 export pathway, typically contained one or both of the NLS and NES motifs, suggesting that they too encode a Rev-like protein. It was not surprising that some βERVs were predicted to contain one motif but not the other, as random mutation since integration is expected to interfere with sequence-based motif prediction. It is also possible that the NES of some betaretroviral Rev-like proteins (such as is the case for HERV-K Rec) are encoded at the exon boundary and/or within a frame different to that used by *env*, making the prediction of NES from the Env protein sequence challenging. A number of βERVs in group VII were found to contain retroviral Tap-binding motifs, defined as published [23], implicating their use of the CTE:Tap export pathway. The presence of putative NLS and NES in some group VII βERVs suggests that Rev-like elements may also be present.

As Rev-like proteins are encoded within the *env* gene, the recombination event that replaced the betaretroviral *env* with a gammaretroviral *env* and gave rise to group VIII would have caused the incidental loss of any encoded Rev-like protein. Such a lineage would only have remained viable if it either possessed an alternative mechanism for export, or never made use of a Rev/RRE equivalent export mechanism in the first place. That Rev-like proteins are widely distributed amongst the betaretroviruses suggests that it is not unreasonable that the progenitor of group VIII did possess a Rev-like protein. This possibility is supported by the existence of the Rej protein of JSRV, as JSRV clusters alongside group VIII in the Type D lineage. In addition, several bat βERVs in groups VI and VII contain putative NLS and NES motifs, suggesting that members of these groups contain Rev-like elements.

If group VIII did lose a Rev-like protein upon acquisition of a gammaretroviral *env*, then two explanations for the lineage’s survival are apparent: i) The recombination event was confined to *env* and the betaretroviral CTE possessed by MPMV and SRV, which is located immediately downstream of *env*, already existed as a redundant export mechanism and remained after the event, or ii) The recombination event included the nucleotide sequence downstream of the *env* gene, and a putative CTE-like element was acquired in the process. With regard to the second possibility it is important to note that the mRNA nuclear export mechanism of gammaretroviruses has not been elucidated and the proposal of a CTE-like element remains hypothetical. However, this notion is supported by the observation that accessory proteins have not been reported for gammaretroviruses, expression of unspliced and singly-spliced viral mRNA would require nuclear export, and that a CTE-like *cis*-acting nuclear export element would necessarily be located in singly-spliced *env* mRNA. In either event, our analysis leads to the surprising implication that the betaretroviruses are part of a fundamentally complex retroviral genus and that one lineage, group VIII, has evolved through gene replacement into a simple retrovirus sub-group that does not possess any distinct accessory proteins or virulence factors.

Using the phylogenetic analysis of retroviral Pol sequences we proposed a pathway through which the genus *Betaretrovirus* may have evolved from its progenitor. This hypothetical evolutionary history paints an interesting picture of a broad and diverse retroviral genus whose distribution may be largely unrestricted by host species barriers. The βERV members of a number of groups are represented in hosts who are distantly related, such as group VIII, which contains host species from bats, primates, rodents, and marsupials. This suggests that cross-species transmission of betaretroviruses is a likely and common occurrence, such that betaretroviruses may be particularly adept at evading host defences. This possibility is intriguing, particularly in light of the wide array of additional ORFs found within the genus that hint at the existence of as yet undiscovered betaretroviral accessory and virulence factors; these could, for example, act as countermeasures to circumvent the action of host intracellular restriction factors that are known to act as barriers to cross-species transmission [54]. The wide distribution of diverse βERVs in bats and rodents suggests that these two largest groups of mammals play a major role as both hosts and cross-species transmitters for betaretroviruses. Bats and rodents are globally distributed, appearing on all continents with the exception of Antarctica [30,55]. As such it appears reasonable to postulate that they have both played a large role in the global spread and evolution of betaretroviruses.

## Conclusions

We have demonstrated the presence of a range of βERVs in mega- and microbats that possess a diversity that cannot be confined to the classical Type B/Type D division. Among their number we identified an intact βERV that may be capable of producing infectious virions, and our LTR analysis indicates that betaretroviruses have been circulating in bat populations throughout their evolution and likely still do.

Our evidence that bats have carried a range of exogenous infectious betaretroviruses and that cross-species transmission has been commonplace has important implications for disease emergence. Indeed, the reported association between the betaretrovirus MMTV and human breast cancer and primary biliary cirrhosis may mean that betaretroviral zoonosis is already causing disease in humans [56-58]. Urban expansion into the natural habitats of bats is gradually increasing the amount of overlap between bat and human environments, and with it the amount of contact between bats and humans [59]. In many countries the practice of hunting bats as a source of consumable bushmeat is common [60]. These circumstances provide the opportunity for retroviral transmission between bats and humans. We propose that the transmission of a betaretroviral infection from bats into humans is possible. As such, it is imperative to continue to survey those viruses present in bats.

## Methods

### Generation of bat transcriptomes

Approval for the use of bat tissue was granted by the Australian Animal Health Laboratories Animal Ethics committee (Protocol AEC1281) and by the Animal Ethics Committee of East China Normal University (Approval Number 20110224). *P. alecto* transcriptome datasets were generated from the non-stimulated thymus tissue of a healthy male juvenile bat and the pooled total RNA obtained from mitogen-stimulated spleen, white blood cells, and lymph node and the unstimulated thymus and bone marrow obtained from one pregnant female and one adult male as described previously [61]. The *P. alecto* transcriptome is accessible through the NCBI Sequence Read Archive (http://www.ncbi.nlm.nih.gov/Traces/sra/) [SRA: SRP008674]. The *R. ferrumequinum* transcriptome was generated using whole brain tissue as published [37]. The *P. alecto* and *R. ferrumequinum* transcriptomes were sequenced using the Illumina next-generation sequencing (NGS) platform as described previously [37,61]. The *P. alecto* transcriptome was assembled using Velvet, Oases, and MIRA software packages as described previously [61]. The *R. ferrumequinum* transcriptome was assembled using the Brujin graph and SOAPdenovo software packages as described previously [37].

The generation of the *R. megaphyllus* transcriptome was conducted as follows: Four wild bats, (one female and 3 male) were caught in the Booloumba Creek caves in Queensland, Australia in November 2006 and tissues from brain, kidney, large and small intestines, liver, lung, spleen, heart, skin, bone and reproductive organs were pooled and stored in RNAlater (Ambion). Total RNA was isolated from the 12 pooled bat tissues using the Qiagen RNeasy kit. DNA was prepared from purified total RNA (2.5 μg per cDNA reaction) using the Evrogen MINT cDNA synthesis kit (CAT # SK001) but with a modified oligodT adapter primer containing the recognition sequence for *Gsu*I (5′ AGCAGTGGTATCAACGCAGAGT CTGGAG(T)_20_ VN). The cDNA was normalized with a duplex specific nuclease (DSN) using a modification of the protocol described in the Evrogen Trimmer cDNA normalization kit (Cat # NK001). After the second limited PCR amplification (12 cycles) with the M2 primer, PCR buffer, primers and enzyme were removed using the Machery Nagel Nucleospin II kit. DNA was then digested overnight with *Gsu*I to remove the 3′ polyA tail adapter sequence so as to remove stretches of homopolymer Ts and As which can effect the 454 sequencing run due to cross-talk (homopolymer flash). Five micrograms of normalized amplified double stranded cDNA was purified using the Machery Nagel Nucleopsin kit with the selective removal of the *Gsu*I digested 43 base pair (bp) 3′polyA adapter sequence using a modification of the binding conditions. Library preparation for Roche 454 sequencing for the GS FLX platform was performed by the Australian Genome Research Facility Ltd, St Lucia, Queensland with sequence output of 74 MB, 374,360 single-end reads with an average read length of 239 bp. CLC Genomics Workbench version 4.5.1 (CLC Bio, Aarhus, Denmark) was used to trim reads based on quality and to remove the Evrogen normalization primer sequence, Subsequent 337,805 reads were *de novo* assembled using CLC Genomics Workbench default settings and BLAST databases were prepared using either *de novo* assembled or trimmed unassembled reads.

### Analysis of bat transcriptomes

To search for evidence of betaretroviral gene expression within the bat transcriptomes we retrieved the genome sequences of extant betaretroviruses from GenBank (http://www.ncbi.nlm.nih.gov/genbank/), specifically: Mouse mammary tumor virus (MMTV) [GenBank: NC_001503.1], Mason-Pfizer monkey virus (MPMV) [GenBank: NC_001550.1], Jaagsiekte sheep retrovirus (JSRV) [GenBank: NC_001494.1], Simian retrovirus (SRV) [GenBank: NC_014474.1], and Squirrel monkey retrovirus (SMR) [GenBank: NC_001514.1]. The *gag*, *pol*, and *env* genes of each genome sequence were translated into protein sequences using the CLC Main Workbench 6.6 (CLC Bio). To identify the transcripts of interest we used the tBLASTn function of the CLC Main Workbench incorporating the following parameters: BLOSUM62 matrix, word size = 3, E-values < 1×10^-10^, gap costs of existence 11, extension 1, and low complexity filtered. To confirm that the transcripts identified were more similar to betaretroviruses than other retroviral genera we performed a reciprocal BLAST analysis of each transcript against the NCBI non-redundant protein database (http://blast.ncbi.nlm.nih.gov/Blast.cgi) using the BLASTx function of the CLC Main Workbench with the following parameters: BLOSUM80 matrix, word size = 3, E-values < 1 × 10^-10^, gap costs of existence 10, extension 1, low complexity filtered, and limit by entrez query = Viruses. Annotated sequences of the full-length betaretroviral sequences included in the phylogenetic analyses (PaERV-βA, PaPol-01, PaEnv-01, and RfEnv-01) are included as Additional file 4.

### Assembly of PaERV-βA

We generated the genomic sequence of PaERV-βA using two transcripts identified in the *P. alecto* transcriptome during the initial BLAST analysis which were aligned using the CLC Main Workbench and trimmed by 245 and 401 nucleotides at the 5′ and 3′ extremities of their overlapping region, respectively.

### Genomic mining

To determine the presence of full-length βERVs in mega- and microbats we retrieved the genomes of *P. vampyrus* and *M. lucifugus* from the Ensembl database (http://www.ensembl.org/index.html). We searched for genomic sequences with similarity to the aforementioned extant betaretroviral proteins by conducting a tBLASTn analysis of the genomes using the CLC Main Workbench with the following parameters: BLOSUM62 matrix, word size = 3, E-values < 1×10^-10^, gap costs of existence 11, extension 1, and low complexity filtered. We searched for genomic sequences with similarity to the betaretroviral transcripts identified in the bat transcriptomes by conducting a tBLASTx analysis of the genomes using the CLC Main Workbench with the following parameters: BLOSUM80 matrix, word size = 3, E-values < 1×10^-10^, low complexity filtered. To sort full-length from fragmented βERVs and various other retroelements within the BLAST output, a script was created using Microsoft Office Excel 2003 (Microsoft Corporation, Redmond, USA) that compared the BLAST data for the Gag, Pol, and Env analyses and identified scaffolds that emerged as a hit in each. The long terminal repeats (LTRs) which were used to delineate the full-length βERVs were identified by subjecting each identified gene scaffold to a BLASTn analysis in which the entire sequence was aligned with itself to identify repeated sequences using the following parameters: Word size = 11, Match score = 1, Mismatch score = −3, gap costs of existence 5, extension 2, and low complexity filtered.

### Annotation of bat βERVs

Transcription promoter elements within the 5′ LTRs of the βERVs were predicted using the online promoter predictor tool NNPP 2.2 [62] (http://www.fruitfly.org/seq_tools/promoter.html). TATA boxes were predicted using the Hamming-Clustering method through the online HCtata tool [63] (http://zeus2.itb.cnr.it/~webgene/wwwHC_tata.html). Poly(A) signal sites were predicted using the Hamming-Clustering method through the online HCpolya tool [63] (http://zeus2.itb.cnr.it/~webgene/wwwHC_polya.html). Primer binding sites were identified by an alignment of the genomic nucleotide sequence between the 5′ LTR and the beginning of the *gag* gene of each βERV against the University of Strasbourg’s online tRNA database [64] (http://trna.bioinf.uni-leipzig.de/DataOutput/Search) using the associated BLAST tool (default parameters). Open reading frames (ORFs) were identified within each βERV using the CLC Main Workbench. The dUTPase protein domains and nucleocapsid zinc knuckles were identified by subjecting the translated *gag* and *pro* genes to a protein family (Pfam) domain search [65] through the CLC Main Workbench using the publicly accessible Pfam database (http://pfam.sanger.ac.uk/). The conserved major homology region (MHR) of Gag and enzymatic active sites of the retroviral protease (DxG), reverse transcriptase (DDD), and integrase (DDE) were identified through a protein sequence alignment, using the Create Alignment function of the CLC Main Workbench, between the Gag, Pro, and Pol of each bat βERV against those of the aforementioned extant betaretroviruses.

### Prediction of RNA export elements

NLS and NES were predicted by analyzing the Env, or if known, the Rev-like protein sequence of each betaretrovirus. NLS were predicted using the online tool cNLS mapper [66] (http://nls-mapper.iab.keio.ac.jp/cgi-bin/NLS_Mapper_form.cgi) with a prediction score threshold of 3.0. NES were predicted using the online tool NetNES 1.1 [67] (http://www.cbs.dtu.dk/services/NetNES/). The strength of each NES prediction within the Env/Rev-like protein is defined as strong if the scores for the neural network model and hidden Markov model, together with the overall NES score, are above the algorithm-assigned threshold. The strength is weak if one of the scores is below the threshold. No NES is predicted for proteins in which more than one score is below the threshold. TBE, DR, and IR were identified by subjecting the nucleotide sequence within and downstream of *env* ending at the poly(A) signal site within the 3′ LTR of each betaretrovirus to a BLASTn analysis in which the sequence was aligned against itself to identify repetitive elements using the following parameters: Word size = 11, Match score = 1, Mismatch score = −3, gap costs of existence 5, extension 2, and low complexity not filtered.

### Sequence alignments

All nucleotide and protein alignments were conducted using the Create Alignment function of the CLC Main Workbench except where stated otherwise.

### Phylogenetic analyses

To determine the evolutionary relationships among the different bat betaretroviruses we inferred the phylogenetic relationships among the Gag, Pol and Env amino acid sequences. All of the reference sequences were downloaded from NCBI (Additional file 2: Table S5) and aligned with bat sequences using MUSCLE [68]. We employed the Gblocks program [69] to remove regions of high sequence diversity and hence uncertain alignment. Phylogenetic relationships were then inferred using the maximum likelihood (ML) method available in PhyML 3.0, employing SPR (subtree pruning and regrafting) branch-swapping [70] and incorporating 1,000 bootstrap replications to determine the robustness of each node. The ProtTest 2.4 program [71] was used to select the best-fit model of amino acid substitution, which was found to be LG+I+Г for all data sets.

### Molecular clock dating

A time-scale for βERV evolution was established as described previously [36] and employing the Bayesian Markov chain Monte Carlo method (MCMC) available in the BEAST v1.7 package [72]. We first acquired the genomic substitution rates (*R*) for mega- and microbats. For this, divergence times of mega- and microbats were taken from the fossil record [28] and used to calibrate date estimates for the rest of the species tree, assuming an uncorrelated lognormal relaxed molecular clock. All phylogenetic trees were inferred using the GTR substitution model and the Yule speciation prior, and the BEAST analyses were run until all relevant parameters converged, with 10% of the MCMC chains discarded as burn-in. The estimated substitution rates were then used to calculate the age of each βERV using the following formula: *T*=(*D*/*R*)/2, where *T* is the invasion time of each βERV (million years), *D* is the number of differences per site among the both 5′ and 3′ LTRs, and *R* is the genomic substitution rate (substitutions per site per year).

### Accession numbers

The GenBank accession numbers of the retroviruses used in this study are listed in Additional file 2: Table S5.

## Abbreviations

βERV: Endogenous betaretrovirus; BLAST: Basic local alignment search tool; cDNA: Complementary deoxyribonucleic acid; CTE: Constitutive transport element; DR: Direct nucleotide repeat; DNA: Deoxyribonucleic acid; DSN: Duplex specific nuclease; dUTPase: Deoxyuridine triphosphatase; Env: Envelope; ERV: Endogenous retrovirus; Gag: Group-specific antigen; HERV: Human endogenous retrovirus; IR: Inverted nucleotide repeat; LTR: Long terminal repeat; Lys 1,2: Lysine 1,2; Lys 3: Lysine 3; MHR: Major homology region; mRNA: Messenger ribonucleic acid; mya: Million years ago; NES: Nuclear export signal; NGS: Next-generation sequencing; NLS: Nuclear localization signal; NSR: Non-sequenced region; nt: Nucleotide; ORF: Open reading frame; PBS: Primer binding site; PCR: Polymerase chain reaction; Pfam: Protein family; Pol: Polymerase; PPT: Polypurine tract; Pro: Protease; RT: Reverse transcriptase; Sag: Super-antigen; TBE: Tap-binding element; tRNA: Transfer ribonucleic acid; TSD: Target site duplication; U3: Unique 3′

## Competing interests

The authors declare that they have no competing interest.

## Authors’ contributions

MT, JAH, JC, and GT conceived the study. JAH, JC and MT performed the analyses, MT generated the *R. megaphyllus* transcriptome, HF collected bats from which tissue was obtained to generate the transcriptome data. All authors contributed to the writing of the paper. All authors have read and approved the submission of the manuscript.

## Supplementary Material

Additional file 1: Figure S1 Alignment of extant and bat betaretroviral primer binding sites (PBS). The PBS of bat endogenous betaretroviruses and those of known extant and exogenous betaretroviruses are aligned and grouped according to the specific lysine tRNA complementary to the PBS. *The PBS complementarity of MlERV-βE is uncertain. **Figure S2.**Alignment of the ORF present in the group VII endogenous betaretroviruses (βERVs) of bats. The region from the beginning of the 5′ LTR to the beginning of the *gag* gene of each group VII bat βERV was aligned and a consensus sequence generated. The annotations belong to the consensus sequence and depict the 5′ LTR, predicted promoter element and TATA boxes, the PBS complementary to tRNA Lys3 (Lys 3 PBS), and an open reading frame (ORF). **Figure S3.** Annotated alignment of the group VIII endogenous betaretroviruses (βERVs) of bats. The region from the end of the *env* gene to the 3′ long terminal repeat (LTR) of each group VIII bat βERV was aligned and a consensus sequence generated. The annotations belong to the consensus sequence and depict an open reading frame (ORF), the beginning of the 3′ LTR, and mutations in PaERV-βA and PvERV-βK that influence the presence of ORFs. Click here for file

Additional file 2: Table S1 Comparison of βERV polymerase sequences to those of known betaretroviruses. **Table S2.** Identification of the target site duplications (TSD) flanking endogenous betaretroviruses. **Table S3.** Comparison of the 5′ and 3′ flanking regions of phylogenetically clustered βERVs. **Table S4.** Analysis of betaretroviral RNA export motifs. **Table S5.** GenBank accession numbers and Ensembl database locations of the retroviruses used in this study. Click here for file

Additional file 3: Figure S4 Unannotated alignment of the full proviral genomes of the group VIII endogenous betaretroviruses (βERVs) of bats. Click here for file

Additional file 4 Annotated sequences of PaERV- βA, PaPol-01, PaEnv-01, and RfEnv-01. Click here for file
