# Supplementary figures and images for: Identification of diverse full-length endogenous betaretroviruses in megabats and microbats
Source: Retrovirology. 2013 Mar 27;10:35. doi: 10.1186/1742-4690-10-35 (PMC3621094; doi:10.1186/1742-4690-10-35)

## Figure S1

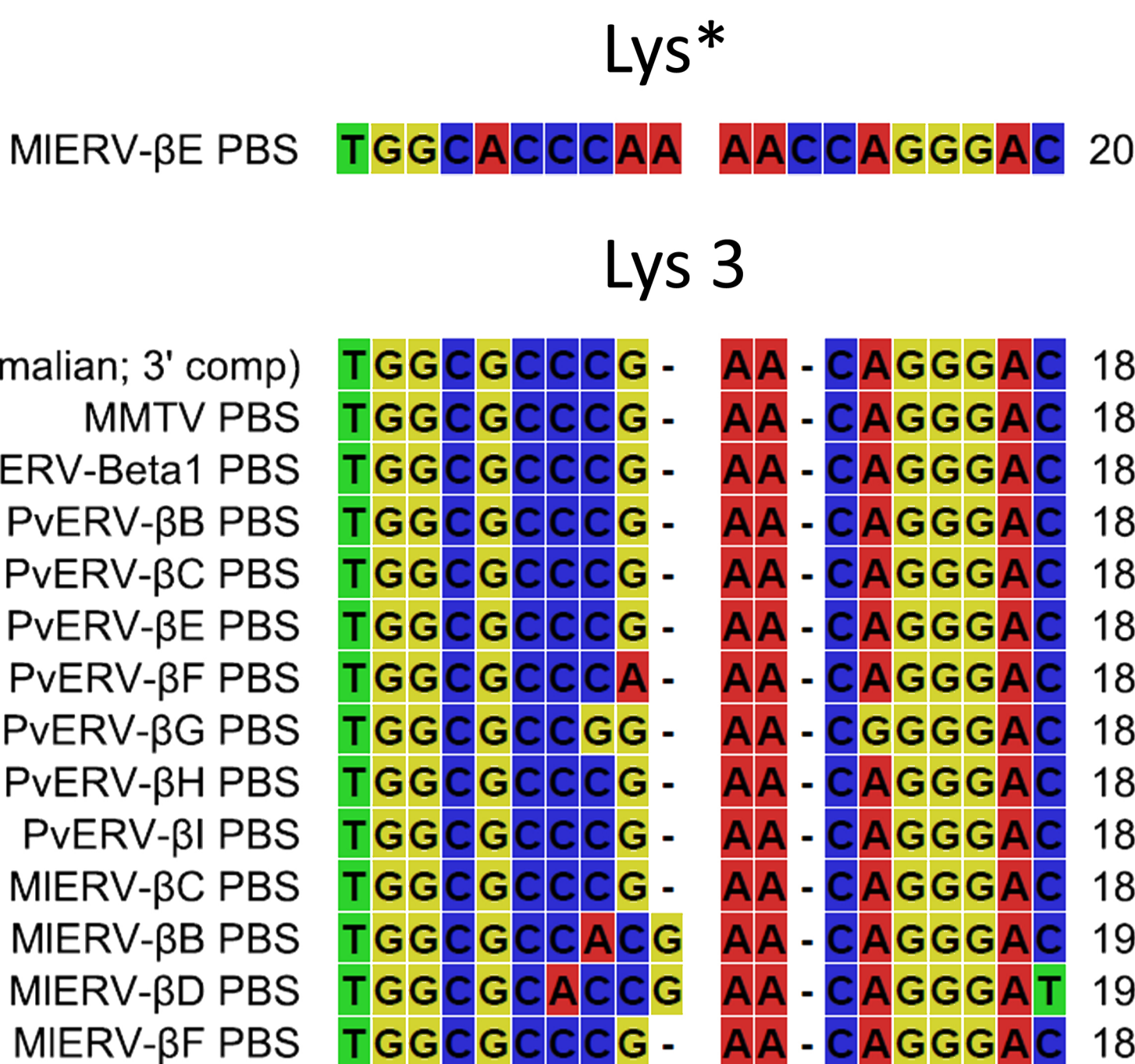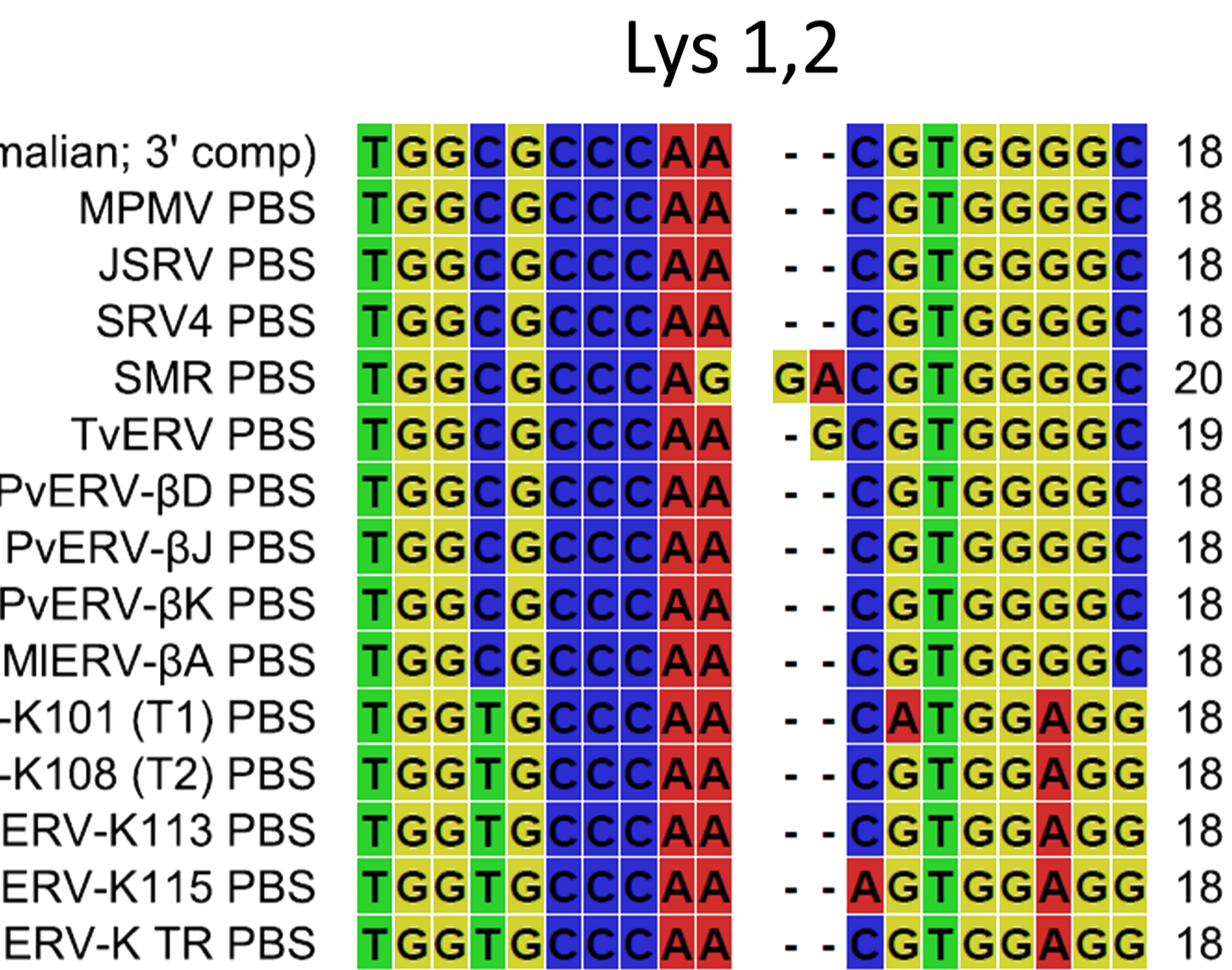

## Figure S2

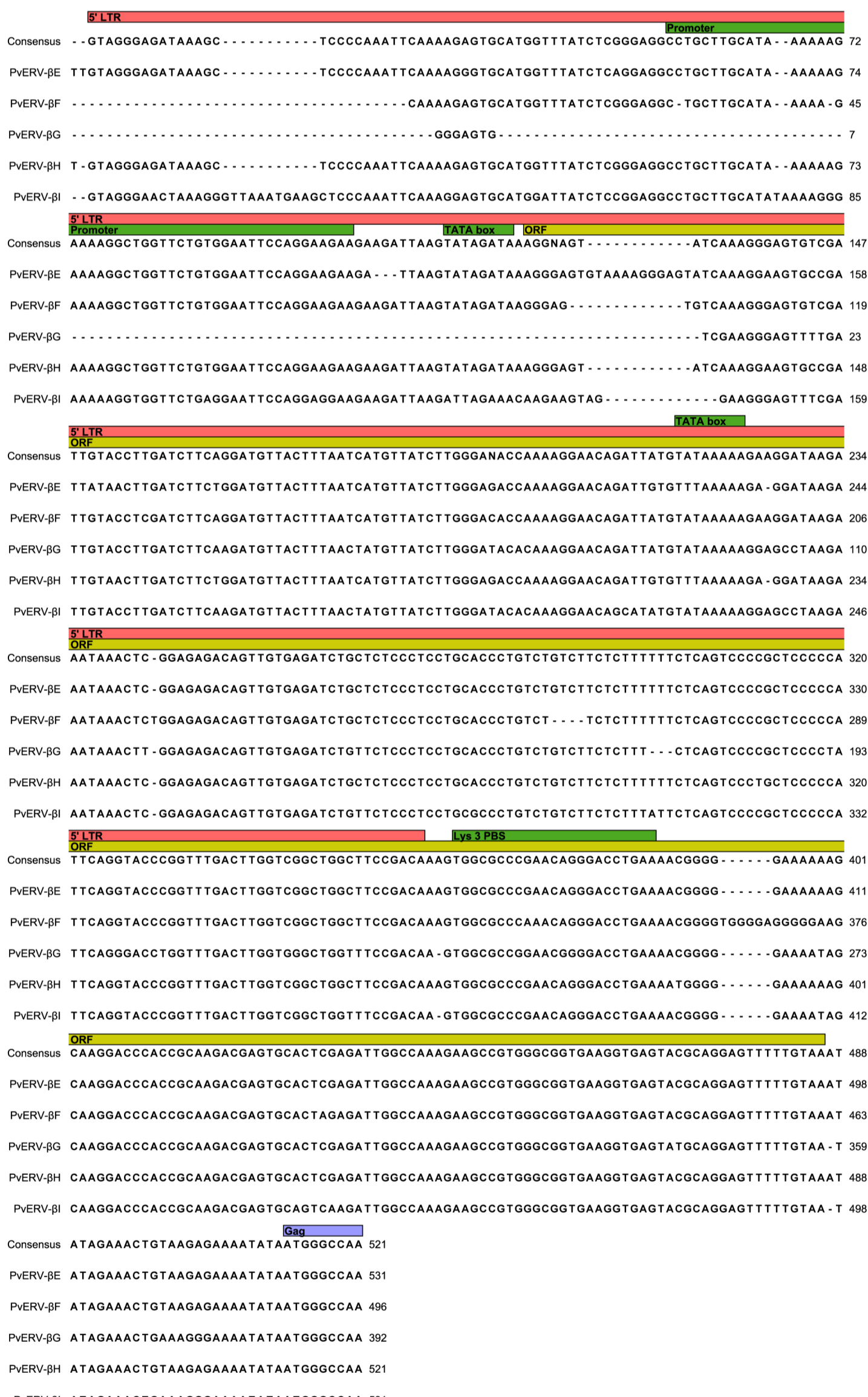

### Figure S3

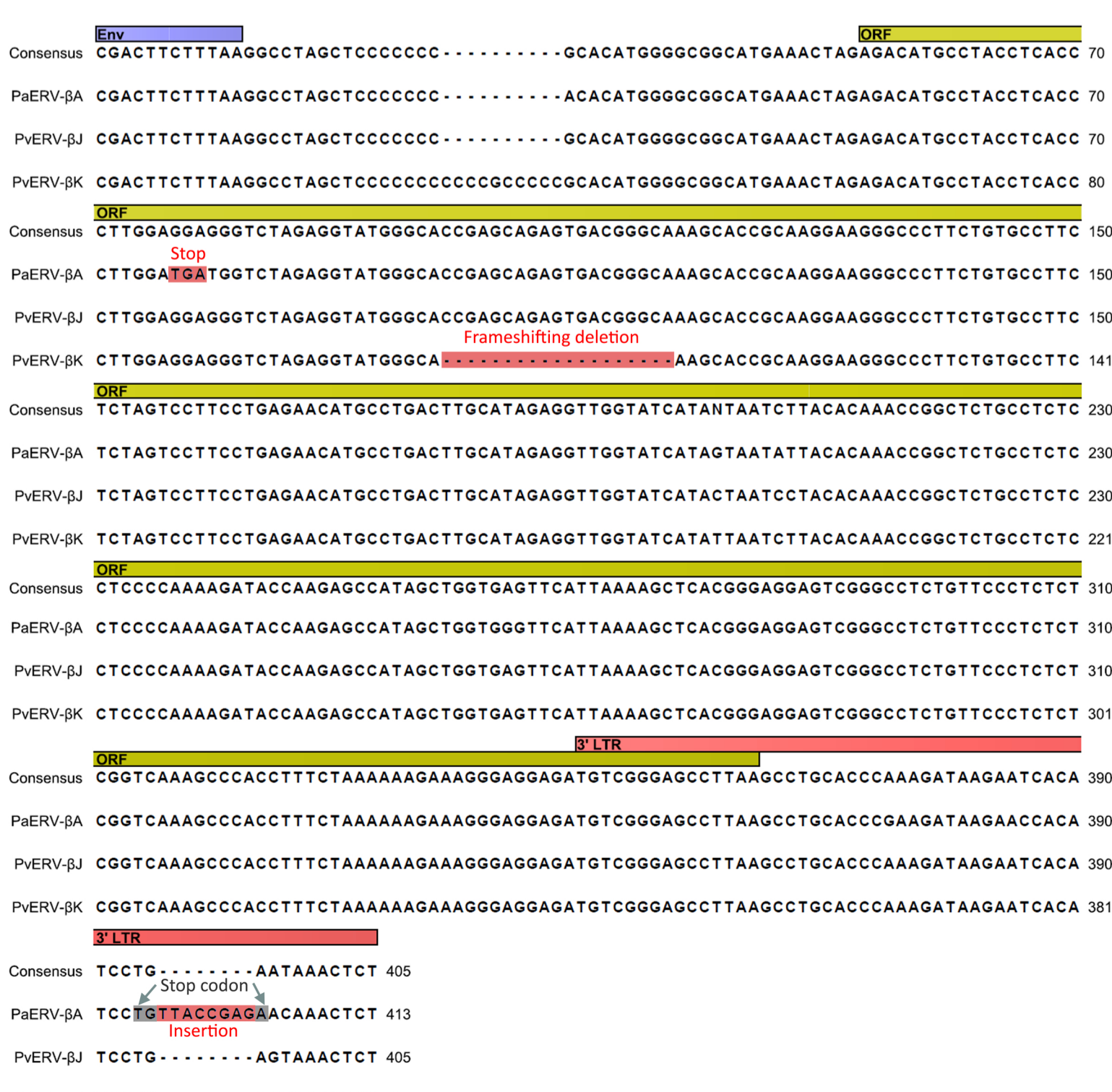

Supplement: Additional file 1: Figure S1 — Alignment of extant and bat betaretroviral primer binding sites (PBS). The PBS of bat endogenous betaretroviruses and those of known extant and exogenous betaretroviruses are aligned and grouped according to the specific lysine tRNA complementary to the PBS. *The PBS complementarity of MlERV-βE is uncertain. Figure S2.Alignment of the ORF present in the group VII endogenous betaretroviruses (βERVs) of bats. The region from the beginning of the 5′ LTR to the beginning of the gag gene of each group VII bat βERV was aligned and a consensus sequence generated. The annotations belong to the consensus sequence and depict the 5′ LTR, predicted promoter element and TATA boxes, the PBS complementary to tRNA Lys3 (Lys 3 PBS), and an open reading frame (ORF). Figure S3. Annotated alignment of the group VIII endogenous betaretroviruses (βERVs) of bats. The region from the end of the env gene to the 3′ long terminal repeat (LTR) of each group VIII bat βERV was aligned and a consensus sequence generated. The annotations belong to the consensus sequence and depict an open reading frame (ORF), the beginning of the 3′ LTR, and mutations in PaERV-βA and PvERV-βK that influence the presence of ORFs. [file 1742-4690-10-35-S1.pdf]
