# Supplementary material for: Identification of diverse full-length endogenous betaretroviruses in megabats and microbats
Source: Retrovirology. 2013 Mar 27;10:35. doi: 10.1186/1742-4690-10-35 (PMC3621094; doi:10.1186/1742-4690-10-35)
Supplement: Additional file 3: Figure S4 — Unannotated alignment of the full proviral genomes of the group VIII endogenous betaretroviruses (βERVs) of bats. [file 1742-4690-10-35-S3.pdf]

```
                                      20                    40                    60                    80
PvERV-βK TGTCGGGAGC CTTAAGCCTG CACCCAAAGA TAAGAATCAC ATCCTGAATA TACTCTGCTA TCTGTGACCA TTTGGGCAGC 80
PvERV-βJ TGTCGGGAGC CTTAAGCCTG CACCCAAAGA TAAGAATCAC ATCCTGAGTA AACTCTGCTA TCTGTGACCA TTTGGACAGC 80
PaERV-βA ---------- ---------- ---------- ---------- ---------- ---------- ---------- ---------- -
                                     100                   120                   140                   160
PvERV-βK CAGGGCACTG GGAAGCGCCG GCCTTGTCTC GTGTCTTTGA TCCCCATTCC CC-TGCCTGC AGAGCGGGAG CCTGCAA-CT 158
PvERV-βJ CAGGGCACTG GGAAGCGCCG GCCTTATCTC GTGTCTTTGA TCCCCATTCC CCCTGCCTGC AGAGAGAGAG CCTGCAAACT 160
PaERV-βA ---------- ---------- ---------- ---------- ---------- ---------- ---------- ---------- -
                                     180                   200                   220                   240
PvERV-βK TCTCACACAG CCCATCTGTT TCTGACCAGC CAGGCAGAAA TCACCTTCTC TGGTGGTCGC CAATAAGCTT GTAAGGAAAT 238
PvERV-βJ TCTCACACAG CCCATCTGTT TCTGACCAGC CAGGCAGAAA TCACCTCCTC TGGTGGTCGC CAATAAGCTT GTAACGAAAT 240
PaERV-βA ---------- ---------- ---------- ---------- ---------- ---------- ---------- ---------- -
                                     260                   280                   300                   320
PvERV-βK ACTCTCTGAT GAGTCCCGCC CCTGCCCTCT CCACCTGAGT GTATAAATAT AACCACTTGA AAATAAAATT TTGAGGCTTG 318
PvERV-βJ GCTCTCTGAT CAGTCCCGCC CCTGCCCTCT CCACCTGAGT GTATAAATAT AACCACTTGA AAATAAAATT TTGAGGCTTG 320
PaERV-βA ---------- ---------- ---------- ---------- ---------- ---------- ---------- ---------- -
                                     340                   360                   380                   400
PvERV-βK ATCAGAACAC TGTCTTGCCT CCACTCTTTT CTCCCGCCCA TGTTTTCTTC AAGGTGCGGT CCTCCTCGGG TTCTACGCAA 398
PvERV-βJ ATCAGAACAC TGTCTTGCCT CCACTCTTTT CTCCCGCCCA TGTTCTCTTC AAGGTGCGGT CCTCCTCGGG TTCTACGCAA 400
PaERV-βA ---------- ---------- ---------- ---------- ---------- ---------- ---------- ---------- -
                                     420                   440                   460                   480
PvERV-βK TAACTAGGTC CTGCGGGTCG GGACAAGTGG CGCCCAACGT GGGGCTCGAG GCACGGACCT TCACCGGACA GCGCAACAAG 478
PvERV-βJ TAACTAGGTC CTGCGGGTCG GGACAAGTGG CGCCCAACGT GGGGCTCGAG GCACGGACCT TCACCGGACA GCGCAACAAG 480
PaERV-βA ---------- ---------- ---------- ---------- ---------- ---------- ---------- ---------- -
                                     500                   520                   540                   560
PvERV-βK TAAAAGGCGT GCACGCAACT AGGAGTGCCT TCCCGGCGAC TGCGACCCCC AGACGTCGGA ACGGCGGGTA AGTCATTGCT 558
PvERV-βJ TAAAAGGCGT GCACGCAACT AGGAGTGCCT TCCCGGCGAC TGCGACCCCC AGACGTCGGA ACGGCGGGTA AGTCATTGCT 560
PaERV-βA ---------- ---------- ---------- ---------- ---------- ---------- ---------A AGTCATTGCT 11
                                     580                   600                   620                   640
PvERV-βK GTCCCATTTT TAA-CATGAG CCATTCAATC TCCAGAGAGG AGACCTTTAT CAAAGAGATA AAGAAGTCGT TAAGGGAGAG 637
PvERV-βJ GTCCCATTTT TAAACATGGG CCATTCAATC TCCAGAGAGG AGACCTTTAT CAAAGAGATA AAGAAGTCGT TAAGGGAGAG 640
PaERV-βA GTCCCATTTT TAA-CATGGG CCAATCTATC TCCAGAGAGG AGTCTTTTGT CAAGGAGATC AAACGGTCTC TAAGGGAGAG 90
                                     660                   680                   700                   720
PvERV-βK AGGAGTTAGG GTTAAGAAAA A-GGATTTGG TTAGTTTTTT CTGTTTTGTT GATGAAAAAT GTCCGTGGTT CATTCTTAGC 716
PvERV-βJ AGGAGTTAGG GTTAAGAAAA A-GGATTTGG TTAGTTTTTT CTGTTTTGTT GATGAAAAAT GTCCGTGGTT CATTCTTAGC 719
PaERV-βA AGGAGTAAGG GTTAAAAAAA AAGGATTTGG TTAGTTTTTT CTGTTTTGTT GATGAAAAAT GTCCGTGGTT CATTCTTAGC 170
                                     740                   760                   780                   800
PvERV-βK GGCCCAGATA TTCATCCCTT GAGTTGGCAA AA-GTAGGAA AAGATTTAAA TAGATTACTA GAAAAAGAGG GACCAAATGC 795
PvERV-βJ GGCCCAGATA TTCATCCCTT GAGTTGGCAA AAAGTAGGAA AAGATTTAAA TAGATTACTA GAAAAAGAGG GACCAAATGC 799
PaERV-βA GGCCCAGATA TTCATCCCTT GAGTTGGCAA AAAGTAGGAA AAGATTTAAA TAGATTACTG GAAAAAGAGG GACCAAATGC 250
                                     820                   840                   860                   880
PvERV-βK TGTCCCCGTT AGTTGTCTCA GTTATTGGGG ACTCATTCGA GATATCATTG AAGGGGCCGA AACTGATAGT AATAAGAGAC 875
PvERV-βJ TGTCCCTGTT AGTTGTCTCA GTTATTGGGG ACTCATTCGA GATATCATTG AAGGGGCCGA AACTGATAGT AATAAGAGAC 879
PaERV-βA TGTGTCCGTT AGTTGTCTCA GTTATTGGGG ACTCATTCGA GACATCATTG AAGGGGCCGA AACTGATAGT AATAAGAGAC 330
                                     900                   920                   940                   960
PvERV-βK AGCTGCTATC GGTAGCCACT GAAACGCTCA AACAGCTCTC TCGCCCCCCA TCCGTTAAAG GTGGAGCTAC CTCTTCCCCC 955
PvERV-βJ AGCTGCTATC GGTAGCCACT GAAACGCTCA AACAGCTCTC TCGCCCCCCA TCCGTTAAAG GTGGAGCTAC CTCTTCCCCC 959
PaERV-βA AGCTGCTATC GGTAGCCACT GAAACGCTCA AACAGCTCTC TCGCCCCCCA TCCGTTAAAG GTGGAGCTAC CTCTTCCCCC 410
                                     980                 1,000                 1,020                 1,040
PvERV-βK TTTCCTTCTG TAGTCGTGGA CATAGAGCCC TCGCCTCCGT TCGGAGACTC TCTTCCCCAA ATATACCCCA TAATACCAAA 1035
PvERV-βJ GT-CCTTCTG TAGTCGTGGA CATAGAGCCC TCGTCTCCGT CTGGAGGCCC TTATTCTCAA ATATACCCCA TAATCCCAAA 1038
PaERV-βA TGTCCTTCTG TAGTCGTGGA CGTAGAGCCC TCGCCTCCGT TCGGAGACTC TCTTCCCCAA ATATACCCCA TAATACCAAA 490
                                   1,060                 1,080                 1,100                 1,120
PvERV-βK ACCCGGCCCT GAACCCCTTG ATCCCGGAGA TGCAACTGTT CTGGAGGACG AGGCCGCCAA ATATCATGAG CCTGTTTGGC 1115
PvERV-βJ ACCCAGCCCT GAACCCCTTG ATCCCGGAGA TGCAACTGTT CTGGAGGACG AGGCCGCCAA ATATCATGAG CCTGTTTGGC 1118
PaERV-βA ACCCGGCCCT GAACCCCTTG ATCCCGGAGA TGCAACTGTT CTGGAGGACG AGGCCGCCAA ATATCATGAG CCTGTTTGGC 570
                                   1,140                 1,160                 1,180                 1,200
PvERV-βK CTTCATCTCA TACCTTTTTT GCTGCAGGGA AGCCTGCCAC GTGCCCCCCT CCTTGCAACC CCCTTCATAT CCCTCAGGCG 1195
PvERV-βJ CTTCATCTCA TACCCTTTTT GCTGCAGGGA AGCCTGCCAC GTGCCCCCCT CCTTACAACC CCCTCCATAT CCCTCAGGCG 1198
PaERV-βA CTTCATCTCA TACCTTTTTT GCTGCAGGGA GGCCTGCCAC ACGCCCCCCT CCTTACAACC CCCTCCATAT CCCTCAGGCG 650
                                   1,220                 1,240                 1,260                 1,280
PvERV-βK GTTCTTGACC CCTTTTTGGA GACAAAGAAA CAACTGCAAC AACAAATTCA AAGCCTCAAA GGCGTCTTTA CTTTACAGAG 1275
PvERV-βJ GTTCTTGACC CCTTTTTGGA GACAAAGAAA CAACTGCAAC AACAAATTCA AAGCCTCAAA GGCGTCTTTA CTTTACAGAG 1278
PaERV-βA GTTCTTGACC CCTTTTTGGA GACAAAGAAA CAACTGCAAC AACAAATTCA AAGCCTCAAA AGCGTCTTTA CTTTACAGAG 730
                                   1,300                 1,320                 1,340                 1,360
PvERV-βK TGAATTGGCT AATCTACAAT TAGAAGTCTC CGCTCTACAA AATTCTATTT TTTCGGGGCC TCAATCTAAG CCCCCTAAGG 1355
PvERV-βJ TGAATTGGCT AATTTACAAC TAGAAGTCTC CGCTCTACAA AATTCTATTT TTTCGGGGCC TCAATCTAAG CCCCCTAAGG 1358
PaERV-βA TGAATTGGCT AATTTACAAT TAGAAGTCTC CGCTCTACAA AATTCTATTT TTTCGGGGCC TCAATCTAAG CCCCCTAAGG 810
                                   1,380                 1,400                 1,420                 1,440
PvERV-βK GACCCCGGGC CAAACCCTTT GTTTTCCCCA TCATGACTCG ATCCGGTACC AGACCCTCAC AAGACCCGGA TACAACTCAG 1435
PvERV-βJ GACCCCGGGC CAAACCCTTT GTTTTCCCCA TCATGACTCG ATCCGGTACC AAACCCTCAC AGGATCCAGA TACAACTCAG 1438
PaERV-βA GACCCCGGGC CAAACCTCTT GCTTTCCCTA TTCTGACTCG ATCAGGTACC AGACCCTCAC AGGACCCGGA TACAACTCAG 890
                                   1,460                 1,480                 1,500                 1,520
PvERV-βK GAGGCGTCTA CTGACGCCCA AGAGGCCTCT AGAGCCAGCA CACCTGATGA CCCAGCTTGT GAGGATGTGG CACAATCTGA 1515
PvERV-βJ AAGGCGTCTA CTGACGCCCA AGAGGCCTCT AGAGCCAGCA CACCTGATGA CCCAGCTTGT GAGGATGTGG CACAATCTGA 1518
PaERV-βA GAGGCGTCTA CTGACGCCCA AGAGGCCCCT AGAGCCAGCA CACCTGATGA CCCAGCTTGT GAGGATGTGG CACAATCTGA 970
                                   1,540                 1,560                 1,580                 1,600
PvERV-βK GGAAGAAGGG AAAAGCCCAC ATAACGATTC TGAAAATGAG GGAGACTATC AGAACCTTTT GCCTACTGTA GAACGGATAA 1595
PvERV-βJ GGAAGAAGGG AAAAGCCCAC ATAACGATTC TGAAAATGAG GGAGACTATC AGAACCTTTT GCCTACTGTA GAACGGATAA 1598
PaERV-βA GGAAGAAGGG AAAAACCCAC ATAACGATTC TGAAAATGAG GGAGACTATC AGAACCTTTT GCCTACTGTA GAACGGATAA 1050
```

```
                   1,620                          1,640                          1,660                          1,680
PvERV-βK AATTTAAAAC TGTCAAAGAT TTACACAGTG CTATAAAGAG CTATGGCCTC ACAGCTCCCT TTACCTTGTC CATACTTGAA 1675
PvERV-βJ AATTTAAAAC TGTCAAAGAT TTACACAGTG CTATAAAGAG CTATGGCCTC ACAGCTCCCT TTACCTTGTC CATACTTGAA 1678
PaERV-βA AATTTAAAAC TGTCAAAGAT TTACACAGTG CTATAAAGAG CTATGGCCTC ACAGCTCCCT TTACCTTGTC CATACTTGAA 1130
                   1,700                          1,720                          1,740                          1,760
PvERV-βK AGTTTGCCTG GCGATGGCTT TCTGTTGCCG GGAGAATGGA TAAAAGTGGC CCAATCTGTG CTGAGTAGGG GAGAATTTTT 1755
PvERV-βJ AGTTTGCCTG GCGATGGCTT TCTGTTGCCG GGAGAATGGA TAAAAGTGGC CCAATCTGTG CTGAGTAGGG GAGAATTTTT 1758
PaERV-βA AGTTTGCCTG GCGATGGCTT TCTGTTGCCG GGAGAATGGA CAAGAGTGGC CCAATCTGTG CTGAGTAGGG GAGAATTTTT 1210
                   1,780                          1,800                          1,820                          1,840
PvERV-βK AACTTGGAAA GCAGAATTTC TTGACAGGGG GGAAACTCAG GCAGTGCGCA ACCAGAAAAA TCCCCGGTCT CCCATGGCCA 1835
PvERV-βJ AACTTGGAAA GCAGAATTTC TTGACAGGGG GGAAACTCAG GCAATGCGCA ACCAGAAAAA TCCCCGGTCT CCCATGGCCA 1838
PaERV-βA AACTTGGAAA GCAGAATTTT TTGACAGGGG GGAAACTCAG GCAATGCGCA ACCAGAAAAA TCCCCGGTCT CCCATGGCCA 1290
                   1,860                          1,880                          1,900                          1,920
PvERV-βK CCTGGACTGC TGATAAGATA TGTGGGAGGG GAGACTTTGC CTCTGAGCGT AAACAACGCA GCTTGTCCAC TGGGATCCTT 1915
PvERV-βJ CCTGGACTGC TGATAAGATA TGTGGGAGGG GAGACTTTGC CTCTGAGCGT AAACAACGCA GCTTGTCCGC TGGGATCCTT 1918
PaERV-βA CCTGGACTGC TGATAAGATA TGTGGGAGGG GAGACTTTGC CTCTGAGCGT AAACAACGCA GTTTGTCTGC TGGGATCCTT 1370
                   1,940                          1,960                          1,980                          2,000
PvERV-βK AGTCAAACAG CGGCCGCCGC TATCGGGGCC TGGAGAGCCA TTCCTCCAAA AGGTTCAGTC ACCACGCCCT TGAGCAAAAT 1995
PvERV-βJ AGTCAAACAG CGGCCGCCGC TATCGGGGCC TGGAGAGCCA TTCCTCCAAA AGGTTCGGTC ACCACGCCCT TGAGCAAAAT 1998
PaERV-βA AGTCAAACAG CGGCCGCTGC TATCGGGGCC TGGAGAGCCA TTCCTCAAAA AGGTTCGGTC ACCACGCCCT TGAGCAAAAT 1450
                   2,020                          2,040                          2,060                          2,080
PvERV-βK AGTTCAGGGG ACCCATGAAC CTTTCTCCCA GTTCGTTGCA CGTTTGCAGG AAACTGCGGA GCGCGTCCTA GGGCCAGAAG 2075
PvERV-βJ AGTTCAGGGG ACCCATGAAC CTTTCTCCCA GTTCGTTGCA CGTTTGCAGG AAACTGTGGA GCGCGTCCTA GGGCCAGAAG 2078
PaERV-βA AGTTCAGGGG CCCCATGAAC CTTTCTCCCA GTTCGTTGCA CGTTTACAGG AAACTGCGGA GCGCGTCCTA GGGCCAGAAG 1530
                   2,100                          2,120                          2,140                          2,160
PvERV-βK ACGGGGAAGG GAAACTTGTA AAACAGCTTG CTTATGAAAA TGCTAATGCT GCCTGTAAAG CGGCTTTAAA GGGGCACATC 2155
PvERV-βJ ACGGGGAAGG GAAACTTGTA AAACAGCTTG CTTATGAAAA TGCTAATGCT GCCTGTAAAG CGGCTTTAAA GGGGCACATC 2158
PaERV-βA ACGGGGAAGG GAAACTTGTA AAACAGCTTG CTTATGAAAA TGCTAATGCT GCCTGTAAAG CGGCTTTAAA GGGGCACCTC 1610
                   2,180                          2,200                          2,220                          2,240
PvERV-βK CGAAATCTCG ATATTCATGG GATGATTCGC TTGTGCAATG ATGTCGATTC CACCGCCCAT CAAATAAAGT TGGCCATCGG 2235
PvERV-βJ CGAAATCTCG ATATTCATGG GATGATTCGC TTGTGCAATG ATGTCGATTC CACCGCCCAT CAAATAAAGT TGACCATCGG 2238
PaERV-βA CGAAATCTCG ATATTCATGG GATGATTCGC TTGTGCAATG ATGTCGATTC CACCGCCCAT CAAATAAAGT TGGCCATTGG 1690
                   2,260                          2,280                          2,300                          2,320
PvERV-βK GGCTGTAATG CAACATGCTA ATAGTAACAC TCCTGTTCCC CAAAATACCC CCATTGCCGC TCGGTGTTGC TTCCGCTGCG 2315
PvERV-βJ GGCTGTAATG CAACATGCTA ATAGTAACAC TCCTGTTCCC CAAAATACCC CCATTGCCGC TCGGTGTTGC TTCCGCTGCG 2318
PaERV-βA GGCTGTAATA CAACATGCTA ATAGTAACAC TCCTGTTCCC CAAAATACCC CCGTTGCCGC TCGGTGTTGC TTCCGCTGCG 1770
                   2,340                          2,360                          2,380                          2,400
PvERV-βK GACAACCTGG ACATGTTGCC CGACAATGCT CACTTCCCTG TACCGGGCAG ATCTTAGCTC CCCCTACAGT TCCTGTTCCC 2395
PvERV-βJ GACAACCTGG ACATGTTGCC CGACAATGCT CACTTCCCTG TACCGGGCAG ATCTTAGCTC CCCCTACCAT TCCTGTTCCC 2398
PaERV-βA GACAACCTGG ACATGTTGCC CGACAATGCT CACTTCCCCG TACCGGGCAG ATCTTAGCTC CCCCTACAGT TCCTGTTCCC 1850
                   2,420                          2,440                          2,460                          2,480
PvERV-βK GTACTCCGCG CTCCGGGATT TTGCCCGCGC TGTATGAAAG GGAAGCATTG AGCTAGTGAG TGCCGCTCTA AAACTGATGT 2475
PvERV-βJ GTACTCCGCG CTCCGGGATT TTGCCCGCGC TGTAAGAGAG GGAAGCATTG GGCTAGTGAG TGCCGCTCTA AAACTGATGT 2478
PaERV-βA GTACCCCGCG CTCCGGGACT TTGCCCGCGT TGTAAGAAAG GGAAGCATTG GGCTAATGAG TGCCGCTCTA AAACTGATGT 1930
                   2,500                          2,520                          2,540                          2,560
PvERV-βK CGCGGGCAAC CCACTTCTTC CCTTGTCGGG AAACGGGAAG AGGGCCCAGC CCCGGGGCCC AACGCCCATC CAATTCCTCC 2555
PvERV-βJ CGCAGGCAAC CCACTTCTTC CCTTGTCGGG AAACGGGAAG AGGGCCCAGC CCCGGGGCCC AACGCCCATC CAATTCCTCC 2558
PaERV-βA CGCGGGCAAC CCACTTCTTC CCTTGTCGGG AAACGGGAAA AGGGCCCAGC CCCGGGGCCC AACGCCCATC CAATTCCTCC 2010
                   2,580                          2,600                          2,620                          2,640
PvERV-βK CGGCTACGGG GCCCCGACAG CCAAACAACC AGGCACCCTC CAACGAGCCA CCCCAGGCAG CGCAGGATTG GACCTCTGTG 2635
PvERV-βJ CGGCTACGGG GCCCCGACAG CCAAACAACC AGGCACCCTC CAACGAGCCA CCCCAGGCAG CGCAGGATTG GACCTCTGTG 2638
PaERV-βA CGGCTACGGG GCCCCGACAG CCGAACAACC AGGCACCCTC CAACGAGCCA CCCCAGGCAG CACAGGATTG GACCTCTGTG 2090
                   2,660                          2,680                          2,700                          2,720
PvERV-βK CCACCTCCGG TACAATATTA ATACCTGAGG ATGGGCCCCA AGTCATTAAT ACTGGGGTCT TCGGTCCTCC CCCTTCTGGA 2715
PvERV-βJ CCACCTCCGG TACAATATTA ATACCTGAGG ATGGGCCCCA AGTCATTAAT ACTGGGGTCT TCGGTCCTCC CCCTTCTGGA 2718
PaERV-βA CCACCTCCGG TATAATATTA ATACCTGAGG ATGGGCCCCA AGTCATTAAT ACTGGGGTCT TCGGTCCTCC CCCTTCTGGA 2170
                   2,740                          2,760                          2,780                          2,800
PvERV-βK ACATACTTCC TAATTCTGGG AC-GGCCTCA GCCACTTTAT CTGGTATTAC CATCTTTCCC TCGGTAGTAG ATGCTGACTA 2794
PvERV-βJ ACATACTTCC TAATTCTGGG ACGGGCCTCA GCCACTTTAT CTGGTATTAC CATCTTTCCC TCGGTAGTAG ATGCTGACTA 2798
PaERV-βA ACATACTTTC TAATTCTGGG ACGGGCCTCA GCCACTTTAT CTGGTATTAC CAACTTTCCC TCGGTAGTAG ATGCTGACTA 2250
                   2,820                          2,840                          2,860                          2,880
PvERV-βK TGAGGGGGAA ATAAAGGTCC TGGCGGCGGC CACGAAAGGG CCTTTAAATA TTTCTCCCGG CCAAAGAATT GCTCAAGCAC 2874
PvERV-βJ TGAGGGGGAA ATAAAGGTCC TGGCGGCGGC CATGAAAGGG CCTTTAAATA TTTCTCCCGG CCAAAGAATT GCTCAAGCAC 2878
PaERV-βA TGAGGGGGAA ATAAAGGTCC TGGCGGCGGC CACGAAAGGG CCTTTAAATA TTTCTCCCGG CCAAAGAATT GCTCAAGCAC 2330
                   2,900                          2,920                          2,940                          2,960
PvERV-βK TCCCTGTGCC TTTTACAAAG GGGCTCCCTC ATAAGGGGGG -TCACCGCGG TGCTTCAAAT CCCGGGTCCT CGGATGCATA 2953
PvERV-βJ TCCCTGTGCC TTTTACAAAG GGACTCCCTC ATAAGGGGGG -TCACCGCGG TGCTTCAAAT CCTGGGTCCT CGGATGCATA 2957
PaERV-βA TCCCTGTGCC TTTTACAAAG GGGCTCCCTC ATAAGGGGGG GTCGCCGCGG TGCTTCAAAT CCCGGGTCCT CGGATGCATA 2410
                   2,980                          3,000                          3,020                          3,040
PvERV-βK CTGGGTCCAA CCCCTTACCA AGGAGCGACC CCTTCTCATG CTGTCTATAG ATGGAAAGTC TTTTGAGGGC TTACTTGATT 3033
PvERV-βJ CTGGGTCCAA CCCCTTACCA AGGAGCGACC CCTTCTCATG CTGTCTATAG ATGGAAAGTC TTTTGAGGGC TTACTTGATT 3037
PaERV-βA CTGGGTCCAA CCCCTTACCA AGGAGCGACC CCTTCTCATG CTGTCTATAG ATGGAAGGTC TTTTGAGGGC TTACTTGATT 2490
                   3,060                          3,080                          3,100                          3,120
PvERV-βK CGGGTGCAGA TTCCACTGTT CTTTCCCAAG AACATTGGCC CTCCTCTTGG CTGTTACAGC CCTCCTCCAC CCACCTTCAA 3113
PvERV-βJ CGGGTGCAGA TTCCACTGTT CTTTCCCAAG AACATTGGCC CTCCTCTTGG CCGTTACAGC CCTCCTCCAC CCACCTTCAA 3117
PaERV-βA CGGGTGCAGA TTCCACCGTT CTTTCCCAAG AACATTGGCC CTCCTCTTGG CCGTTACAGC CCTCCTCCAC CCACCTTCAA 2570
                   3,140                          3,160                          3,180                          3,200
PvERV-βK GGTATTGGCC AATCTCATAA TACCCTACAG AGTAGCAAGA TTCTTACGTG GTCAGATGGA GAAGGAAACT CTGGTCAAAT 3193
PvERV-βJ GGTATTGGCC AATCTCATAA TACCCTACAG AGTAGCAAGA TTCTTACGTG GTCAGATGGA GAAGGAAACT CTGGTCAAAT 3197
PaERV-βA GGTATTGGCC AATCTCATAA TACCCTACAG AGTAGCAAGA TTCTCACGTG GTCAAATGGA GAGGGAAACT CTGGTCAAAT 2650
```

```
                          3,220                     3,240                     3,260                     3,280
PvERV-βK CCAACCCTTT GTGGTCCCAG GTCTACCTGT TAACTTGTGG GGGAGAGATA TTTTGGCACA AATGGGGGCA ATATTATGCA 3273
PvERV-βJ CCAACCCTTT GTGGTCCCAG GTCTACCTGT TAACTTGTGG GGGAGAGATA TTTTGGCACA AATGGGGGCA ATATTATGCA 3277
PaERV-βA CCAACCCTTT GTGGTCCCAG GTCTACCTGT TAACTTGTGG GGGAGAGATA TTCTGGCACA AATGGGGGCA ATATTATGCA 2730
                          3,300                     3,320                     3,340                     3,360
PvERV-βK GCCCTAACGA GGTGGTCACC AAACAAATGT TACAATCTGG GTTCATACCT GGTAAAGGAC TTGGAAAATC TAATCAGGGA 3353
PvERV-βJ GCCCTAACGA GGTGGTCACC AAACAAATGT TACAATCTGG GTTCATACCT GGTAAAGGAC TTGGAAAATC TAATCAGGGA 3357
PaERV-βA GCCCTAACGA GGTGGTCACC AAACAAATGT TACAACCTGG GTTCATACCT GGTAAAGGGC TTGGAAAATC TAATCAGGGA 2810
                          3,380                     3,400                     3,420                     3,440
PvERV-βK ATTAACTCAC CCATTGAGAC CACCCCAAAA ACAAACCGTC ATGGTCTAGG GTATAAGGAG CATTTTTCAT AAGGGTTACT 3433
PvERV-βJ ATTAACTCAC CCATTGAGAC CACCTCAAAA ACAAACCGTC ATGGTCTAGG GTATAAGGAG CATTTTTCAT AAGGGTTACT 3437
PaERV-βA ATTAACTCAC CCATTGAGAC CACCCCAAAA ACAAACTGTC ATGGTCTAGG GTATAAGGAG CATTTTTCAT AAGGGTTACT 2890
                          3,460                     3,480                     3,500                     3,520
PvERV-βK GATCCTCCTG CACCCCAGGC GGATAAGATC CATTGGAAAT CAGGGGATCC TGTCTGGGTT GATCAGTGGC CCCTCCCCTC 3513
PvERV-βJ GATCCTCCTG CACCCCAGGC GGATAAGATC CATTGGAAAT CAGGGGATCC TGTCTGGGTT GATCAGTGGC CCCTCCCTTC 3517
PaERV-βA GATCCTCCTG CACCCCAGGC GGATAAGATC CATTGGAAAT CAGGGGATCC TGTCTGGGTT GATCAGTGGC CCCTCCCCTC 2970
                          3,540                     3,560                     3,580                     3,600
PvERV-βK TTATAAGATT TCGGCCGCCC TCCGTTTAGT GCAGGAACAA TTAAAGGCTG GCCATTTAGA GCCCTCTACC TCTCCTTGGA 3593
PvERV-βJ TTATAAGATT TCGGCCGCCC TCCGTTTAGT GCAGGAACAA TTACAGGCTG GCCATTTAGA GCCCTCTACC TCTCCTTGGA 3597
PaERV-βA TTATAAGATT TCGGCCGCCC TCCGTTTAGT GCAGGAACAA TTAGGGGCTG GCCGTTTGGG GCCCTCTTTT TCC------A 3044
                          3,620                     3,640                     3,660                     3,680
PvERV-βK ATACCCCCAT TTTCGTTATT CAAAAAAAGA ATGGTACCTG GAGGCTTTTA CAGGACCTAA GAGAAGTTAA TAAAACAATG 3673
PvERV-βJ ATACCCCCAT TTTCGTTATT CAAAAAAAGA ATGGTACCTG GAGGCTTTTA CAGGACCTAA GAGAAGTTAA TAAAACAATG 3677
PaERV-βA GTAATCTCA- ---------- -------AGA ATGGTACCTG AAGGCTTTTA CAGGACCTAA GAAAAGTTAA TAAAACAATG 3106
                          3,700                     3,720                     3,740                     3,760
PvERV-βK CAAATCATGG GGCCCTTACA GCCTGGGCTG CCTTCCCCCG TTGCTATTCC CAACAAGTTC TTTAAAATTG TCATTGATCT 3753
PvERV-βJ CAAATCATGG GGCCCTTACA GCCTGGGCTG CCTTCCCCCG TTGCTATTCC CAACAAGTTC TTTAAAATTG TCATTGATCT 3757
PaERV-βA CAAATCATGG GGCCCTTACA GCCTGGGCTG CCTTCCCCCG TTGCTATTCC CAACAAGTTC TTTAAAATTG TCATTGATCT 3186
                          3,780                     3,800                     3,820                     3,840
PvERV-βK CAAGGATTGC TTTTTCTCTA TACCTTTGCA CCCTGATGAT CGCCCACGTT TTGCTTTTAG TATCCCCATT ACCAATCATG 3833
PvERV-βJ CAAGGATTGC TTTTTCTCTA TACCTTTGCA CCCTGATGAT CGCCCACGTT TTGCTTTTAG TATCCCCATT ACCAATCATG 3837
PaERV-βA CAAGGATTGC TTTTTCTCTA TACCTTTACA CCCTGATGAT CGCCCACGTT TTGCTTTTAG TATCCCCATT ACCAATCATG 3266
                          3,860                     3,880                     3,900                     3,920
PvERV-βK TTGGACCAAC ACCCCGCTTT CAGTGGCGGG TTCTCCCCCA GGGGATGGCA AACAGCCCTA CTTTGTGTCA AAAATACGTG 3913
PvERV-βJ TTGGACCAAC ACCCCGCTTT CAGTGGCGGG TTCTCCCCCA GGGGATGGCA AACAGCCCTA CTTTGTGTCA AAAATACGTG 3917
PaERV-βA TTGGACCAAC ACCCCGCTTT CAGTGGCGGG TTCTACCCCA GGGGATGGCA AACAGCCCTA CTTTGTGTCA AAAATACGTG 3346
                          3,940                     3,960                     3,980                     4,000
PvERV-βK GCCCAAACAA TAGACCCATT CAGGGTCCGT TACTCTACTC TTTATATTGT ACATTACATG GATGATATTC TTGTAGCAGG 3993
PvERV-βJ GCCCAAACAA TAGACCCATT CAGGGTCCGT TACTCTACTC TTTATATTGT ACATTACATG GATGATATTC TTGTAGCAGG 3997
PaERV-βA GCCCAAACAA TAGACCCATT CAGGGTCCGT TACTCTACTC TTTATATTGT ACATTACATG GATGATATTC TTGTAGCAGG 3426
                          4,020                     4,040                     4,060                     4,080
PvERV-βK TCCCTCCCCA CAATTGACTC ATCAGGTCAC CCAGGAACTA ATTGCTGCAT TGAGCAAAAG AGGGTTTGTT ATTGCCCCTG 4073
PvERV-βJ TCCCTCCCCA CAATTGACTC ATCAGGTCAC CCAGGAACTA ATTGCTGCAT TGAGCAAAAG AGGGTTTGTT GTTGCCCCTG 4077
PaERV-βA TCCCTCCCCA CAATTGACTC ATCAGGTCAC CCAGGAACTA ATTGCTGCAT TGAGCAAAAG AGGGTTTGTT ATTGCCCCTG 3506
                          4,100                     4,120                     4,140                     4,160
PvERV-βK AAAAGGTACA AACTCAACCC CCCTTACTTT TTTTGGGGTT TGAACTGCAC GCCAACCGGG TAATGTCACA AAAAACTCAA 4153
PvERV-βJ AAAAGGTACA AACTCAACCC CCCTTACTTT TTTTGGGGTT TGAACTGCAC GCCAACCGGG TAATGTCACA AAAAACTCAA 4157
PaERV-βA AAAAGGTACA AACCCAACCC CCCTTACTTT TTTGGGGGTT TGAACTGCAC GCCAACTGGG TAATGTCACG AAAAACTCAA 3586
                          4,180                     4,200                     4,220                     4,240
PvERV-βK TTCAAAACTT CATCTCTAAA AACATTGAAT GATTTTCAAA AGCTGTTGGG AGACATTAAC TGGCTTCGCC CTTACTTAAA 4233
PvERV-βJ TTCAAAACTT CATCTCTAAA AACATTGAAT GATTTTCAAA AGCTGTTGGG AGACATTAAC TGGCTTCGCC CTTACTTAAA 4237
PaERV-βA TTCAGAACTT CATCTCTAAA AACATTGAAT GATTTTCAAA AACTGTTGGG AGACATTAAC TGGCTTCGCC CTTACTTAAA 3666
                          4,260                     4,280                     4,300                     4,320
PvERV-βK ATTAACCACA GGGGATCTTA AACCGTTGTT TGAGATTTTA CAAGGAGACC CTAATCCCGC TTCCCTACGT TCCCTAACAA 4313
PvERV-βJ ATTAACCACA GGGGATCTTA AACCGTTGTT TGAGATTTTA CAAGGAGACC CTAATCCCGC TTCCCTACGT TCCCTAACAA 4317
PaERV-βA ATTGACCACA GGGGATCTTA AACCGTTGTT TGAGATTTTA CAAGGAGACC CCAATCCCGC TTCCCTACGT TCCCTAACGA 3746
                          4,340                     4,360                     4,380                     4,400
PvERV-βK AAGAAGCTTC ACAGGCTCTC TCCCTGGTAG AGGAGGCTAT CGAACAACAG TTCTCGGGGT ACTTTGATCC CCTACAATCA 4393
PvERV-βJ AAGAAGCTTC ACAGGCTCTC TCCCTGGTAG AGGAGGCTAT CGAACAACAG TTCTCAGGGT ACTTTGATCC CCTACAATCA 4397
PaERV-βA AAGAAGCTTC ACAGGCTCTC TCCCTGGTAG AGGAGGCTAT CGAACAACAG TTCTTGGGGT ACTTTGATCC CCTACAATCG 3826
                          4,420                     4,440                     4,460                     4,480
PvERV-βK CTGTGGTTTC TTGTTCTGCC CACCGCCTTC ACGCCCACTG GCCTACTCTG GCAGGGTGAC CCCTTATTCT GGGTACATCT 4473
PvERV-βJ CTGTGGTTTC TTGTTCTGCC CACCGCCTTC ACGCCCACTG GCCTACTCTG GCAGGGTGAC CCCTTATTCT GGGTACATCT 4477
PaERV-βA CTGTGGTTTC TTGTTCTGCC CACCGCCTTC ATACCCACTG GCCTACTCTG GCAGGGTGAC CCCTTATTCT GGGTACATCT 3906
                          4,500                     4,520                     4,540                     4,560
PvERV-βK CTCTGCTACC CCCTCAAAGG TCCTACCGTC TTATCCTTCT TTGGTTTGCA GTTTAATTCG CCTAGGACGG CTCTCAGCAA 4553
PvERV-βJ CTCTGCTACC CCCTCAAAGG TCCTACCGTC TTATCCTTCC TTGGTTTGCA GTTTAATTCG CCTAGGACGG CTCTCAGCAA 4557
PaERV-βA CTCTGCTACC CCCTCAAAGG TCCTACCATC TTATCCTTCT TTGGTTTGCA GTTTAATTCG CCTAGGATGG CTCTCAGCAA 3986
                          4,580                     4,600                     4,620                     4,640
PvERV-βK TAAGAATATT TGGCAAAGAA CCTGACATTA TTGTGACCCC CTATGATAAA GCCCAGAGAC ATTGGCTAGA ACAGTTTGAC 4633
PvERV-βJ TAAGAATATT TGGCAAAGAA CCTGACATTA TTGTGACCCC CTATGATAAA GCCCAGAGAC ATTGGCTAGA ACAGTTTGAC 4637
PaERV-βA TAAGAATATT TGGCAAAGAA CCTGACATTA TTGTGATCCC CTATGATAAA GCCCAAAGAC ATTGGCTAGA ACAGTTTGAC 4066
                          4,660                     4,680                     4,700                     4,720
PvERV-βK CCGGAATGGG CTTTAACGTG CACCTCATAT CCTGGGAAGT TTGATAACCA CTACCCTGCC AATAGACTGG TTCAGTTCTT 4713
PvERV-βJ CCAGAATGGG CTTTAACGTG CACCTCATAT CCTGGGAAGT TTGATAACCA CTACCCTGCC AATAGACTGG TTCAGTTCTT 4717
PaERV-βA CCGGAATGGG CTTTAACGTG CACCTCATAT CCTGGGAAGT TTGATAACCA CTACCCTGCC AATAGACTGG TTCAGTTCTT 4146
                          4,740                     4,760                     4,780                     4,800
PvERV-βK AACTACCACA GCAGTGGTTT TCCCCCAAGT TACAAAGAAC CAACCATTGC CCCAGGCTAC TCTAGTTTTT GTCGACGGCT 4793
PvERV-βJ AACTACCACA GCAGTGGTTT TCCCCCAAGT TACAAAGAAC CAACCATTGC CCCAGGCTAC TCTAGTTTTT GTCGACGGCT 4797
PaERV-βA AACTACCACT GCAGTGGTTT TCCCCCAAGT TACAAAGAAC CAACCATTGC CCCAGGCTAC TCTAGTTTTT GTCGATGGCT 4226
```

```
                              4,820                  4,840                  4,860                  4,880
                                  |                      |                      |                      |
PvERV-βK CCTCAAATGG CCGGGCCGCC TTCGACATCA ATGGGGAGAT TAGCTGCTTT CAAACAACTT ATACCTCTGC GCAATTGGTG 4873
PvERV-βJ CCTCAAATGG CCGGGCCGCC TTCGACATCA ATGGGGAGAT TAGCTGCTTT CAAACAACTT ATACCTCTGC ACAACTGGTG 4877
PaERV-βA CCTCAAATGG TCGGGCCGCC TTCGACATCG ATGGGGAGAT TAGCTGCTTT CAAACAACTT ATACCTCTGC ACAATTGGTG 4306
                              4,900                  4,920                  4,940                  4,960
                                  |                      |                      |                      |
PvERV-βK GAATTACAAG CTGTAATTGC TGTTTTTTCC AAGCTGCCTA CTACTCCCTT TAACCTGTAT TCTGACAGTG CTTACGTAGT 4953
PvERV-βJ GAATTACAAG CTGTAATTGC TGTTTTTTCC AAGCTGCCTA CTACTCCCTT TAACCTGTAT TCTGACAGTG CTTACGTAGT 4957
PaERV-βA GAATTACAAG CTGTAATCGC TGTTTTTTCT AAGCTGCCTA CTACTCCCTT TAACCTGTAT TCTGACAGCG CTTACGTAGT 4386
                              4,980                  5,000                  5,020                  5,040
                                  |                      |                      |                      |
PvERV-βK AAATGCTGTT CGTACCTTAG AAACAGTTCC CTTAATCAAG CCATCCTCCG CCGCTTCTCA ACTTTTTGCC ACCCTACAGC 5033
PvERV-βJ AAATGCTGTT CGTACCTTAG AAACAGTTCC CTTAATCAAG CCATCCTCCG CCGCTTCTCA ACTTTTTGCC ACCCTACAGC 5037
PaERV-βA AAATGCTGTT CGTACCTTAG AAACAGTTCC CTTAATCAAG CCATCCTCCG CCGCTTCTCA ACTTTTTGCC ACCCTACAGC 4466
                              5,060                  5,080                  5,100                  5,120
                                  |                      |                      |                      |
PvERV-βK AGCTAATTGT GAACAGAAAA CATCCATTCT TTATTGGACA CATTCGCGCT CACTCAGGTC TACCCGGTCC TTTGTCAAAA 5113
PvERV-βJ AGCTAATTGT GAACAGAAAA CATCCATTCT TTATTGGACA CATTCGCGCT CACTCAGGTC TACCCGGTCC TTTGTCAAAA 5117
PaERV-βA AGCTAATTGT GAACAGAAAA CATCCATTCT TTATTGGACA CATTCGCGCT CACTCAGGTC TACCCGGTCC TTTGTCGAAA 4546
                              5,140                  5,160                  5,180                  5,200
                                  |                      |                      |                      |
PvERV-βK GGCAATGATC GGGTGGACCA GGCTACTCGC CTAGTGGCCA TTTCATTTAC AGATCCCCTG GCTGAAGCAA AGAAAGCCCA 5193
PvERV-βJ GGCAATGATC GGGTGGACCA GGCTACTCGC CTAGTGGCCA TTTCATTTAC AGATCCCCTG GCTGAAGCAA AGAAAGCCCA 5197
PaERV-βA GGCAATAATC GAGTGGACCA GGCTACTCGC CTAGTGGCCA TCTCACTTAC AGATCCCCTG GCTGAAGCAA AGAAAGCCCA 4626
                              5,220                  5,240                  5,260                  5,280
                                  |                      |                      |                      |
PvERV-βK TACTCTGCAT CACCTCAATG CTAATACCCT GAGACACATG TTTAAAATTA CTAGGGAACA GGCCAGAGAC ATTGTAAAAG 5273
PvERV-βJ TACTCTGCAT CACCTCAATG CTAATACCCT GAGACACATG TTTAAAATTA CTAGGGAACA GGCCAGAGAC ATTGTAAAAG 5277
PaERV-βA TACTCTACAT CACCTCAATG CTAATACCCT GAGACACATG TTTAAAATTA CTAGGGAACA GGCCAGAGAC GTTGTAAAAG 4706
                              5,300                  5,320                  5,340                  5,360
                                  |                      |                      |                      |
PvERV-βK GTTGTAAAAC TTGTGTCACC TTCCTTCCGG AACCACATCT TGGGTTTAAT CCCAGGGGCC TAATTCCTGG AGAACTGTGG 5353
PvERV-βJ GTTGTAAAAC TTGTGTCACC TTCCTTCCGG AACCACATCT TGGGGTTAAT CCCAGGGGCC TAATTCCTGG AGAACTGTGG 5357
PaERV-βA GTTGTAAAAC TTGTGTCACC TTCCTTTCGG AACCACACCT TGGGGTTAAT CCCAGGGGCC TAATTCCTGG AGAACTGTGG 4786
                              5,380                  5,400                  5,420                  5,440
                                  |                      |                      |                      |
PvERV-βK CAAATGGACG TGACCCATTA CCCTTCCTTT GGTACGTTGA AATACCTCCA TGTAACTATA GATACCTTTA GTGGCTACCT 5433
PvERV-βJ CAAATGGACG TGACCCATTA CCCTTCCTTT GGTACGTTGA AATACCTCCA TGTAACTATA GATACCTTTA GTGGCTACCT 5437
PaERV-βA CAAATGGACG TAACCCATTA CCCTTCCTTT GGTACGTTGA AATACCTCCA TGTAACTATA GATACCTTTA GTGGCTACCT 4866
                              5,460                  5,480                  5,500                  5,520
                                  |                      |                      |                      |
PvERV-βK GTTTGCTACT GCCCAAACAG GTGAGGCTAC TAAACATGTT GTGTCCCATC TTATAGCCTG CTTTGCTCAC CTGGGGGTAC 5513
PvERV-βJ GTTTGCTACT GCCCAAACAG GTGAGGCTAC TAAACATGTT GTGTCCCATC TTATAGCCTG CTTTGCTCAC CTGGGGGTAC 5517
PaERV-βA GTTTGCTACT GCCCAAACAG GCGAAGCTAC TAAACATGTT GTGTCCCATC TTATAGCCTG CTTTGCTCAC CTGGGGGTAC 4946
                              5,540                  5,560                  5,580                  5,600
                                  |                      |                      |                      |
PvERV-βK CTAAGATTAT AAAAACAGAC AATGGCCCAG GGTATACTAG TTCCTCTTTA AAACAATTTT GTGCTCAAAT GCAGGTCAAA 5593
PvERV-βJ CTAAGATTAT AAAAACAGAC AATGGCCCAG GGTATACTAG TTCCTCTTTA AAACAATTTT GTGCTCAAAT GCAGGTCAAA 5597
PaERV-βA CTAAGATTAT AAAAACAGAC AATGGCCCAG G-TATACCAG TTCCTCTTTA AAACAATTTT GTGCTCAAAT GCAGGTCAAA 5025
                              5,620                  5,640                  5,660                  5,680
                                  |                      |                      |                      |
PvERV-βK CATGTTACAG GTATTCCCTA TAACCCACAG GGGCAGGGTA TAGTAGAGAG AGCTCACCTC ACCCTTAAGA CCATGCTAAC 5673
PvERV-βJ CATGTTACAG GTATTCCCTA TAACCCACAG GGGCAGGGTA TAGTAGAGAG AGCTCACCTC ACCCTTAAGA CCATGCTAAC 5677
PaERV-βA CATGTTACAG GTATTCCCTA TAACCCACAG GGACAGGGTA TAGTAGAGAG AGCTCACCTC ACCCTTAAGA CCATGCTAAC 5105
                              5,700                  5,720                  5,740                  5,760
                                  |                      |                      |                      |
PvERV-βK CAAACTTGCC ACATCAGGGG GATTACTATA TCCCCAAAAA GGAACACAAA AAACCCTTCT TAATCATGCC CTGTTTGTTT 5753
PvERV-βJ CAAACTTGCC ACATCAGGGG GATTACTATA TCCCCAAAAA GGAACACAAA AAACCCTTCT TAATCATGCC CTGTTTGTTT 5757
PaERV-βA CAAACTTGCC ACATCAGGGG GATTACTATA TCCCCAAAAA GGAACACAAA AAACCCTTCT TAATCATGCC CTGTTTGTTT 5185
                              5,780                  5,800                  5,820                  5,840
                                  |                      |                      |                      |
PvERV-βK TAAATTTCTT GTCTCTTGAC AATGCGGGTC GCTCCGCTGC AGACCGCTTC TGGCACCCCC AAACCGCGTC AAACTTCGTT 5833
PvERV-βJ TAAATTTCTT GTCTCTTGAC AATGCGGGTC GCTCCGCTGC AGACCGCTTC TGGCACCCCC AAACCGCGTC AAACTTTGTT 5837
PaERV-βA TAAATTTCTT GTCTCTTGAC AATGCGGGCC GCTCCGCTGC AGACCGCTTC TGGCACCCCC AGACCGCGTC AAACTTCGCT 5265
                              5,860                  5,880                  5,900                  5,920
                                  |                      |                      |                      |
PvERV-βK ACAGCCCTGT GGAAGGACCC ACTTACCGCT CAATGGCATG GGCCCCACCC AGTATTAATC TGGGGAAAAG GACATGCTTG 5913
PvERV-βJ ACAGCCCTGT GGAAGGACCC ACTTACCGCT CAATGGCATG GGCCCCACCC AGTATTAATC TGGGGAAAAG GACATGCTTG 5917
PaERV-βA ACAGCCCTGT GGAAGGACCC ACTTACCGCT CAATGGCGCG GGCCCGACCC AATATTAATC TGGGGAAAAG GACATGCTTG 5345
                              5,940                  5,960                  5,980                  6,000
                                  |                      |                      |                      |
PvERV-βK TATTTATGAT TCAGCCGCAC AGAACGCGCG CTGGTTACCC GACAGGCTAA TTAAGCCATT TAACCCTACC CAGGGTGGCC 5993
PvERV-βJ TATTTATGAT TCAGCTGCAC AGAACGCGCG CTGGTTACCC GACAGGCTAA TTAAGCCATT TAACCCTACC CAGGGTGGCC 5997
PaERV-βA TATTTATGAT TCAGCCGCAC AGAACGCGCG CTGGTTACCC GACAGGCTAA TTAAGCCATT TAACCGTACC CAGGGTGGCC 5425
                              6,020                  6,040                  6,060                  6,080
                                  |                      |                      |                      |
PvERV-βK CCTGAGAAGC TTTCTCTGCT TAATTTCAGA ATGATGTTCC TTCTGCTCAT GTTTCTGCTG CCCTCCTGCA TCGCTCATAG 6073
PvERV-βJ CCTGAGAAGC TTTCTCTGCT TAATTTCAGA ATGATGTTCC TTCTGCTCGT GTTTCTGCTG CCCTCCTGCA TCGCGCATAG 6077
PaERV-βA CCTGAGAAGC TTTCTCTGCT TAATTTCAGA ATGATGTTCC TCCTGCTCAT GTTTCTGCTG CCCTCCTGCA TCGCTCATAG 5505
                              6,100                  6,120                  6,140                  6,160
                                  |                      |                      |                      |
PvERV-βK AATATTCAAC TTTACTTGGA CTGTCGTCAA TGAAGCGGGT GACATTGCTT ATACTACCTC AACTTTGGCA AGTACCACCC 6153
PvERV-βJ AATATTCAAC TTTACTTGGA CTGTCGTCAA TGAAGCGGGT GACATTGCTT ATACTACCTC AACTTTGGCA AGTACAACCC 6157
PaERV-βA GATATTCAAC TTTACTTGGA CTGTCGTCAA TGAAGCGGGT GACATTGCTT ATACTACCTC AACCTTGGCA AGTACAACCC 5585
                              6,180                  6,200                  6,220                  6,240
                                  |                      |                      |                      |
PvERV-βK CATGGCCTAC ACTCACTCCA GACCTATGCA GCCTAGCTGC CGGAGCTGCA CCAGCCTGGG GACTCCCGGA TACTTTTCTC 6233
PvERV-βJ CATGGCCTAC ACTTACTCCA GACCTATGCG GCCTAGCTGC CGGAGCCGCA CCAGCCTGGG GACTCCCGGA TACTTTTCTC 6237
PaERV-βA CATGGCCTAC ACTCACTCCA GACTTATGCA GCCTAGCTGC CAGAGCATCA CCAGCCTGGG GACTTCCAGA TGTTTTTCTC 5665
                              6,260                  6,280                  6,300                  6,320
                                  |                      |                      |                      |
PvERV-βK CCCCTTTC-- -CAAGGCACC AAGTAGCCCC CA-------- -CCAGAGAGA TCAATTTTTT GCCCCTGCAG GGTGTAATAC 6301
PvERV-βJ CCCCTTTC-- -CAAGGCACC TAGTAGCCCC CA-------- -CCAGAGAGA TCAATTTTTT GCCCCTGCAG GGTGTAATAC 6305
PaERV-βA CCCTTGCCTG ACAAGCCGCC CAATAACCCC TCTGTAGCAG CCCGCTGGAA TGGGGGCTGT GGTTC-ACAA TCCGGGAGAC 5744
                              6,340                  6,360                  6,380                  6,400
                                  |                      |                      |                      |
PvERV-βK ACCCCGTAGG AGGACCTTT- ---------- --CTTAGAGA ATCTGATTTT TATGTCTGCC CAGGCGGGCA CAGAAGTAGA 6368
PvERV-βJ ACCCCGTAGG AGGACCTTT- ---------- --CTTAGAGA ATCTGATTTT TATGTCTGCC CAGGCGGGCA CAGAAGTAGA 6372
PaERV-βA GCTTCTTTAA GGGGCCCTTA GGGAAAAACG AACCTACAGG CCCTGCTCTT TATGTTTGCC CAGGCGGGCA CAGAAGCCGG 5824
```

```
                                 6,420                 6,440                 6,460                 6,480
PvERV-βK AACCTTAATT ATCGATGTGG ATTCAGAGAT TCCTCCTTTT GTGCCTCCTG GGGATGTGAA ACTACTGGCG ACGCCTACTG 6448
PvERV-βJ AACCTTAATT ATCGATGTGG ATTCAGAGAT TCCTCCTTTT GTGCCTCCTG GGGATGTGAA ACTACTGGCG ACGCCTACTG 6452
PaERV-βA ACCCTTAATC ACCAGTGTGG GTTTAGAGAA TCTTACTATT GTGCCTCCTG GGGGTGTGAA ACCACGGGCG ATGCCTACTG 5904
                                 6,500                 6,520                 6,540                 6,560
PvERV-βK GCACCCTGTC TCTACATGGG ATTATATAAC CGTTAAAAAA GGTTGGAGTA ACTCCCGGCG TAACAATACA CCCACCCCTG 6528
PvERV-βJ GCACCCTGTC TCTACATGGG ATTATATAAC CGTTAAAAAG GGTTGGAGTA ACTCCCGGCG TAACGATACA CCCACCCCTG 6532
PaERV-βA GCACCCTGTC TCGACTTGGG ATTATATAAT AGTCAAAAAG GGCTGGAATA ATTCCCAGGC TGA------- --------TG 5969
                                 6,580                 6,600                 6,620                 6,640
PvERV-βK AATGCCAGAG TGCACATACA ACCCAAGGAT GGTGTACTCC CCTTCTTATT TCCTTTACCG ATAAAGGAAA AAGAGCACCC 6608
PvERV-βJ AATGCCAGAG TGCACATACA ACCCAAGGAT GGTGCACTCC CCTTCTTATT TCCTTTACCG ATAAAGGGAA AAGAGCACCC 6612
PaERV-βA AGAACGATTG TGGACA---- -----AAAGT GGTGTACTCC CCTCCTCATT TCCTTTACTG ATGAAGGGAA AAGAGCGCCC 6040
                                 6,660                 6,680                 6,700                 6,720
PvERV-βK CTTGTGGGAT GGACAAGGGG ATATGAATGG GGCCTGAGAC TTGATGCAAT TGGAATGGAT CCAGGGTTCA CATTTAAGGT 6688
PvERV-βJ CTTGAGGGAT GGACAAGGGG ACATGAATGG GGCCTGAGAG CCTATGTAAG TGGAACAGAT CCAGGGTTCA CATTCAAGGT 6692
PaERV-βA CTCGTGGGAT GGACAAGGGG ACATGAATGG GGCCTAAGAC TTTATGTAAC TGGAACAGAT CCAGGGTTCA CATTCAAGGT 6120
                                 6,740                 6,760                 6,780                 6,800
PvERV-βK GAAGCTAATT CGCTCCTCAC CTAATACTGA ACCAAAAATT GTTTTAGGCC CAAATAAAGC CCTGTCTCGG CCGCAAAGCC 6768
PvERV-βJ GAAGCTAATT CGCTCCTCAC CTAATACTGA ACCAAAAATT GTTTTAGGCC CAAATAAAGC CCTGTCTCGG CCGCAAAGCC 6772
PaERV-βA AAAGTTAATT CTCTCCACAC CTAATACT-A ACCAAAAATT GTTTTAGGCC CAAATAAAGC CCTGTCTCGG CCGCAAAGCC 6199
                                 6,820                 6,840                 6,860                 6,880
PvERV-βK CCCAAACACC CCTCCGGGAG AATACTCCTC TCCCTCCCCA AAGCACTGCC TATCAAGCCC GTTTGCCCCC CGTTAGGCTT 6848
PvERV-βJ CCCAAACACC CCTCCAAGAG AATACTCCTC TCCCTCCCCA AAGCACTGCC TATCAAGCCC GTTTGCCCCC CGTTAGGCTT 6852
PaERV-βA CCCAAACACC CCTCCGGGAG AATACTCCTC TCCCTCCCCA GAGCACTGCC TATCAAGCCC GTTTGCCCCC CGTTAGGCCT 6279
                                 6,900                 6,920                 6,940                 6,960
PvERV-βK TCCACAGGTG AGGCCCTAAA GGCTCTAGCT AATGCCACTG CACAGTCCCT TAACACCTCG ACCTATGAGG ATTGCTGGAT 6928
PvERV-βJ TCCACAGGTG AGGCCCTAAA GGCCCTAGCT AATGCCACTG CACAGTCCCT TAACACCTCG ACCTATGAGG ATTGCTGGAT 6932
PaERV-βA TCCACAGGTG AGGCCCTAAA GGCCCTAGCT AATGCCACTG CACAGTCCCT TAACACCTCG ACCT--GAGG ATTGTTGGAT 6357
                                 6,980                 7,000                 7,020                 7,040
PvERV-βK GTGTTTTTCC CCTGTTCCCC CTCTTTATGA AGGCATTGCA ACTGTCTCGC CAGACAAGAC CTATACCAAT GATTCACGGC 7008
PvERV-βJ GTGTTTTTCC CCTGTTCCCC CTCTTTATGA AGGCATTGCA ACTGTCTCGC CAGACATGAC CTATACCAAT GATTCACGGC 7012
PaERV-βA GTGTTTTTCC CCTGTTCCCC CTTTTTATGA AGGCATTGCA ACTGTCTCGC CAGACATGAC CTATACCAAT GATTCACGGC 6437
                                 7,060                 7,080                 7,100                 7,120
PvERV-βK AGACACGCTG GGTGGACTCT CCTTTTTCTG AGTCCTCTCC CGGGCTCACA TTGGCCCAGT TATCAGGAAT TGGCCTCTGC 7088
PvERV-βJ AGACACGCTG GGTGGACTCT CCTTTTTCTG AGTCCTCTCC CGGGCTTACA TTGGCCCAAT TATCAGGAAT TGGCCTCTGC 7092
PaERV-βA AGACACGCTG GGTGGACTCT CCTTTTTCTG AGTCCTCTCC CGGGCTCACA TTGGCCCAGT TGTCAGGAAT TGGCCTCTGC 6517
                                 7,140                 7,160                 7,180                 7,200
PvERV-βK ATTCATAGTA CCTCATTGCT TCTTCCCCCT GAATTATTGC CTATTTGCAA TACCTCCCAT ACGCCTCTAA CACAACACCA 7168
PvERV-βJ ATTCATAGTA CCTCATTGCT TCTTCCCCCT GAATTGTTGC CTATTTGCAA TACCTCCCAT ACGCCTCTAA CACAACACCA 7172
PaERV-βA ATTCATAGTA CCTCATTGCT TCTTCCCCCT GAATTGCTAC CTATTTGCAA TACTTCCCAT ACACCTCTAA CTCAACACCA 6597
                                 7,220                 7,240                 7,260                 7,280
PvERV-βK TTTCCTTGTT GCTCCCCAAG GAACGTATTT TGCTTGCTCT TTTGGAATAA CCCCTTCTAT TGTCCCCCAG TTATTGGTAG 7248
PvERV-βJ TTTCCTTGTA GCTCCCCAAG GAATGTATTT TGCTTGCTCT TTTGGAATAA CCCCATCTAT TGTCCCCCAG TTATTGGTAG 7252
PaERV-βA TTTCCTTGTT GCTCCCCAAG GCATGTATTT TGCTTGCTCT TTTGGAATAA CCCCTTCTAT TGTCCCTCAG TTATTGGTAG 6677
                                 7,300                 7,320                 7,340                 7,360
PvERV-βK AAAATCATGA ATATTGTGTC CTAGTTGTGC TGCTACCTAA GGTCTCAATA CATCCTCCTG AAGATTTGAT TCCCTTTTAT 7328
PvERV-βJ AAAATCATGA ATATTGTGTC TTAGTTGTGC TGCTACCTAA GGTCTCAATA CATCCTCCTG AAGATTTGAT TCCCTTTTAT 7332
PaERV-βA AAAATCATGA ATATTGTGTC CTAGTTGTGC TAATACCTAA GGTCTCAATA CATCCTCCTG AAGATTTGAT TCCCTTTTAT 6757
                                 7,380                 7,400                 7,420                 7,440
PvERV-βK CATAGTGCCC CGCGTGTCAA AAGGGAACCT GTAACTGCCA TCACTCTGGC AGTCCTCTTG GGGTTGGGGG CCACCGGAGC 7408
PvERV-βJ CATAGTGCCC CGCGTGTCAA AAGGGAACCT GTAACTGCCA TCACTCTGGC AGTCCTCTTG GGGTTGGGGG CCACCGGAGC 7412
PaERV-βA CATAGCGCCC CGCGTGTCAA AAGGGAACCT GTAACTGCCA TCACTCTGGC AGTCCTCTTG GGGTTGGGGG CCACCAGAGC 6837
                                 7,460                 7,480                 7,500                 7,520
PvERV-βK AGGGACCGGC ATTGCCTCTA TAATTACTAC AAATCAACAA TTCCATACCC TTAGCTTGGC CATAGATAAA GACATTCAAA 7488
PvERV-βJ AGGGACCGGC ATTGCCTCTA TAATTACTAC AAATCAACAA TTCCATACCC TTAGCTTGGC CATAGATAAA GACATTCAAA 7492
PaERV-βA CGGGACCGGC ATTGCCTCTA TAATCACAAC AAATCAACAA TTCCATACTC TTAGTTTGGC TATAGATAAG GACATTCAAA 6917
                                 7,540                 7,560                 7,580                 7,600
PvERV-βK ATCTGCAAGA AGGCCTTGAT AACCTTAAAG AATCTGTTGT TTCACTTTCT GAGGTAGTTC TTCAAAATCG CCGCGGTCTT 7568
PvERV-βJ ATCTGCAAGA AGGCCTTGAT AACCTTAAAG AATCTGTTGT TTCACTTTCT GAGGTAGTTC TTCAAAATCG CCGCGGTCTT 7572
PaERV-βA ATCTGCAAGA AGGCCTTGAT AACCTTAAAG AATCTGTTGT TTCACTTTCT GAGGTAGTTC TTCAAAATCG CCGCGGTCTT 6997
                                 7,620                 7,640                 7,660                 7,680
PvERV-βK GACCTTCTAT TTCTTAAAGA AGGCGGTCTG TGCGCTGCCC TTAAAGAAGA ATGTTGTTTT TACAAAGATA AAACTGGGTT 7648
PvERV-βJ GACCTTCTAT TTCTTAAAGA AGGCGGTCTG TGCGCTGCCC TTAAAGAAGA ATGTTGTTTT TACAAAGATA AAACTGGGTT 7652
PaERV-βA GACCTTCTAT TTCTTAAAGA AGGCGGTCTG TGCGCTGCCC TTAAAGAAGA ATGTTGTTTT TACAAAGATA AAACTGGGTT 7077
                                 7,700                 7,720                 7,740                 7,760
PvERV-βK AGTCCAAGAC AGTATTGAAA AAATAAAGAA ATATCTGGAG ACCCGGCAAA AACAAAGAGA AAAGGACGAA GCCTGGTATA 7728
PvERV-βJ AGTCCAAGAC AGTATTGAAA AAATAAAGAA ATATCTGGAG ACCCGGCAAA AACAAAGAGA AAAGGACGAA GCCTGGTATA 7732
PaERV-βA AGTCCAAAAC AGTATTGAAA AAATAAAGAA AAATCTGGAG ACCCGGCAAA AACAAAGAGA AAAGGACGAA GCCTGGTATA 7157
                                 7,780                 7,800                 7,820                 7,840
PvERV-βK AAAGTTGGGT CTCTAACA-C CCCCTGGCTG TCCACCCTAC TTCCTACTAT TCTCGGACCT CTAGCAGGGC TCCTCCTTTT 7807
PvERV-βJ AAAGTTGGGT CTCTAACA-C CCCCTGGCTG TCCACCCTAC TTCCTACTAT TCTCGGACCT CTAGCAGGGC TCCTCCTTTT 7811
PaERV-βA AAAGTTGGGC CTCTAAAAAC CCCCTAGCTG TCCACCCTAC TTCCTACTAT TCTCGGACCT CTAGCAGGGT TCCTCCTTTT 7237
                                 7,860                 7,880                 7,900                 7,920
PvERV-βK ACTGTCGATT GGCCCCTGGG CAGTGCAAAA ACTAACTGCT TTTATTAAAG CACAGGTTGA TCAACTAGTT AAACCAGCTG 7887
PvERV-βJ ACTGTCGATT GGCCCCTGGG CAGTGCAAAA ACTAACTGCT TTTATTAAAG CACAGGTTGA TCAACTAGTT AAACCAGCTG 7891
PaERV-βA ACTGTCGATT GGCCCCTGGG CAGTGCAAAA ACTAACTGCT TTTATTAAAG CACAGGTTAA TCAACTAACT AAACCAGCTG 7317
                                 7,940                 7,960                 7,980                 8,000
PvERV-βK TTGCCGTCCA CTACCACCAC TTGACAACTC AGGATGATGA CGACGTGGAG CAAGATCCCC GGCACCCTAG AAATCTTAAC 7967
PvERV-βJ TTGCCGTCCA CTACCACCAC TTGACAACTC AGGATGATGA CGACGTGGAG CAAGATCCCC GGCACCCTAG AAATCTTAAC 7971
PaERV-βA TTGCCGTCCA CTACCACCAT TTGACAACTC AGGATGATGA CGATGTGGAA CAAGATCCCC GGCACCCTAG AAATCTTAAC 7397
```

```
                                        8,020                               8,040                               8,060                               8,080
                                            |                                   |                                   |                                   |
PvERV-βK  CCCTCTAATA CACCCCTGCG CCTGCATCGA CTTCTTTAAG GCCTAGCTCC CCCCCCCCCG CCCCCGCACA TGGGGCGGCA 8047
PvERV-βJ  CCCTCTAATA CACCCCTGCG CCTGCATCGA CTTCTTTAAG GCCTAGCTCC CCCCC----- -----GCACA TGGGGCGGCA 8041
PaERV-βA  CCCTCTAATA CACCCCTGCG CCTGCATCGA CTTCTTTAAG GCCTAGCTCC CCCCC----- -----ACACA TGGGGCGGCA 7467
                                        8,100                               8,120                               8,140                               8,160
                                            |                                   |                                   |                                   |
PvERV-βK  TGAAACTAGA GACATGCCTA CCTCACCCTT GGAGGAGGGT CTAGAGGTAT GGGCA----- ---------- ----AAGCAC 8108
PvERV-βJ  TGAAACTAGA GACATGCCTA CCTCACCCTT GGAGGAGGGT CTAGAGGTAT GGGCACCGAG CAGAGTGACG GGCAAAGCAC 8121
PaERV-βA  TGAAACTAGA GACATGCCTA CCTCACCCTT GGATGATGGT CTAGAGGTAT GGGCACCGAG CAGAGTGACG GGCAAAGCAC 7547
                                        8,180                               8,200                               8,220                               8,240
                                            |                                   |                                   |                                   |
PvERV-βK  CGCAAGGAAG GGCCCTTCTG TGCCTTCTCT AGTCCTTCCT GAGAACATGC CTGACTTGCA TAGAGGTTGG TATCATATTA 8188
PvERV-βJ  CGCAAGGAAG GGCCCTTCTG TGCCTTCTCT AGTCCTTCCT GAGAACATGC CTGACTTGCA TAGAGGTTGG TATCATACTA 8201
PaERV-βA  CGCAAGGAAG GGCCCTTCTG TGCCTTCTCT AGTCCTTCCT GAGAACATGC CTGACTTGCA TAGAGGTTGG TATCATAGTA 7627
                                        8,260                               8,280                               8,300                               8,320
                                            |                                   |                                   |                                   |
PvERV-βK  ATCTTACACA AACCGGCTCT GCCTCTCCTC CCCAAAAGAT ACCAAGAGCC ATAGCTGGTG AGTTCATTAA AAGCTCACGG 8268
PvERV-βJ  ATCCTACACA AACCGGCTCT GCCTCTCCTC CCCAAAAGAT ACCAAGAGCC ATAGCTGGTG AGTTCATTAA AAGCTCACGG 8281
PaERV-βA  ATATTACACA AACCGGCTCT GCCTCTCCTC CCCAAAAGAT ACCAAGAGCC ATAGCTGGTG GGTTCATTAA AAGCTCACGG 7707
                                        8,340                               8,360                               8,380                               8,400
                                            |                                   |                                   |                                   |
PvERV-βK  GAGGAGTCGG GCCTCTGTTC CCTCTCTCGG TCAAAGCCCA CCTTTCTAAA AAAGAAAGGG AGGAGATGTC GGGAGCCTTA 8348
PvERV-βJ  GAGGAGTCGG GCCTCTGTTC CCTCTCTCGG TCAAAGCCCA CCTTTCTAAA AAAGAAAGGG AGGAGATGTC GGGAGCCTTA 8361
PaERV-βA  GAGGAGTCGG GCCTCTGTTC CCTCTCTCGG TCAAAGCCCA CCTTTCTAAA AAAGAAAGGG AGGAGATGTC GGGAGCCTTA 7787
                                        8,420                               8,440                               8,460                               8,480
                                            |                                   |                                   |                                   |
PvERV-βK  AGCCTGCACC CAAAGATAAG AATCACATCC TG-------- AATAAACTCT GCTATCTGTG ACCATTTGGG CAGCCAGGGC 8420
PvERV-βJ  AGCCTGCACC CAAAGATAAG AATCACATCC TG-------- AGTAAACTCT GCTATCTGTG ACCATTTGGA CAGCCAGGGC 8433
PaERV-βA  AGCCTGCACC CGAAGATAAG AACCACATCC TGTTACCGAG AACAAACTCT GCTATCTGTG AC--TTTGGA CAGCCACGGC 7865
                                        8,500                               8,520                               8,540                               8,560
                                            |                                   |                                   |                                   |
PvERV-βK  ACTGGGAAGC GCCGGCCTTA TCTCGTGTCT TTGATCCCCA TTCCCC-TGC CTGCAGAGCG GGAGCCTGCA ACTTCTCACA 8499
PvERV-βJ  ACTGGGAAGC ACCGGCCTTA TCTCGTGTCT TTGATCCCCA TTCCCCCTGC CTGCAGAGAG AGAGCCTGCA ACTTCTCACA 8513
PaERV-βA  ACTGTGAAGC ACCCGCCATT TCCCGTGTCT TTGTTCCCCA TTCCGCC-AC CTGCAGAGTG GGAGCCTGCA ACTGCTGGCA 7944
                                        8,580                               8,600                               8,620                               8,640
                                            |                                   |                                   |                                   |
PvERV-βK  CAGCCCATCT GTTTCTGACC AGCCAGGCAG AAATCACCTT CTCTGGTGGT CGCCAATAAG CTTGTAACGA AATACTCTCT 8579
PvERV-βJ  CAGCCCATCT GTTTCTGACC AGCCAGGCAG AAATCACCTC CTCTGGTGGT CGCCAATAAG CTTGTAACGA AATGCTCTCT 8593
PaERV-βA  CAGCCCATCT GTTTCTGACT AGCCAAGTAG AAATCACCTC ATCTGGTGGT CGCCAATAAG CTTGTAACGA A-TACTCTCT 8023
                                        8,660                               8,680                               8,700                               8,720
                                            |                                   |                                   |                                   |
PvERV-βK  GATCAGTCCC GCCCCTGCCC TCTCCACCTG AG-------- ---------- ---------- ---------- ---------- 8611
PvERV-βJ  GATCAGTCCC GCCCCTGCCC TCTCCACCTG AGTGTATAAA TATAACCACT TGATAATAAA ATTTTGAGGC TTGATCAGAA 8673
PaERV-βA  GATCAGTCCC TCCCCTGCCC TCTCCACTTG AGTTTATAAA TATAACCGCT CGAAAATAAA ATTTTGAGGC TTGATCAGAA 8103
                                        8,740                               8,760                               8,780                               8,800
                                            |                                   |                                   |                                   |
PvERV-βK  ---------- ---------- ---------- ---------- ---------- ---------- ---------- ---------- 8611
PvERV-βJ  CACTGTCTTG CCTCCACTCT TTTCTCCCGC CCATGTTCTC TTCAAGGTGC GGTCCTCCTC GGGTTCTACG CAATAACTAG 8753
PaERV-βA  ---------- ---------- ---------- ---------- ---------- ---------- ---------- ---------- 8103
                                        8,820
                                            |
PvERV-βK  ---------- ---------- 8611
PvERV-βJ  GTCCTGCGGG TCGGGACAAG 8773
PaERV-βA  ---------- ---------- 8103
```
