# Supplementary material for: Identification of diverse full-length endogenous betaretroviruses in megabats and microbats
Source: Retrovirology. 2013 Mar 27;10:35. doi: 10.1186/1742-4690-10-35 (PMC3621094; doi:10.1186/1742-4690-10-35)
Supplement: Additional file 4 — Annotated sequences of PaERV- βA, PaPol-01, PaEnv-01, and RfEnv-01. [file 1742-4690-10-35-S4.doc]

**Pteropus alecto Endogenous Retrovirus – Betaretrovirus A (PaERV-βA)**

LOCUS PaERV-BetaA 8103 bp DNA linear UNA

DEFINITION PaERV-BetaA = Pteropus alecto Endogenous Retrovirus - Betaretrovirus A

Viruses; Retro-transcribing viruses; Retroviridae

COMMENT Assembled from two overlapping transcripts identified within the Pteropus alecto transcriptome; Locus23_188 & Locus27_401.

After trimming by 245 and 401 nt at the 5' and 3' extremities of their overlapping region, the two transcripts overlapped by 3,156 nucleotides with 100% identity.

FEATURES Location/Qualifiers

Protein_region 26..2105

/note="Compiled from Gag CDS regions"

/Translation=MGQSISREESFVKEIKRSLRERGVRVKKKDLVSFFCFVDEKCPWFI

LSGPDIHPLSWQKVGKDLNRLLEKEGPNAVSVSCLSYWGLIRDIIEGAETDSNKRQLLS

VATETLKQLSRPPSVKGGATSSPCPSVVVDVEPSPPFGDSLPQIYPIIPKPGPEPLDPG

DATVLEDEAAKYHEPVWPSSHTFFAAGRPATRPPPYNPLHIPQAVLDPFLETKKQLQQQ

IQSLKSVFTLQSELANLQLEVSALQNSIFSGPQSKPPKGPRAKPLAFPILTRSGTRPSQ

DPDTTQEASTDAQEAPRASTPDDPACEDVAQSEEEGKNPHNDSENEGDYQNLLPTVERI

KFKTVKDLHSAIKSYGLTAPFTLSILESLPGDGFLLPGEWTRVAQSVLSRGEFLTWKAE

FFDRGETQAMRNQKNPRSPMATWTADKICGRGDFASERKQRSLSAGILSQTAAAAIGAW

RAIPQKGSVTTPLSKIVQGPHEPFSQFVARLQETAERVLGPEDGEGKLVKQLAYENANA

ACKAALKGHLRNLDIHGMIRLCNDVDSTAHQIKLAIGAVIQHANSNTPVPQNTPVAARC

CFRCGQPGHVARQCSLPRTGQILAPPTVPVPVPRAPGLCPRCKKGKHWANECRSKTDVA

GNPLLPLSGNGKRAQPRGPTPIQFLPATGPRQPNNQAPSNEPPQAAQDWTSVPPPV*

/label=Gag

CDS 26..106

/label="Gag (Frame 2)"

Mutation 107

/label="Frameshift (Insertion)"

CDS 108..2105

/label="Gag (Frame 3)"

MHR join(1456..1459,1467..1469,1473..1475,1491..1496)

/note="P24 major homology region"

Zinc_knuckle 1752..1805

/note="Nucleocapsid zinc knuckle"

/label=Zk-1

Zinc_knuckle 1866..1919

/label=Zk-2

Slippage_site 1958

/label="Gag / Gag-Pro Slippage site"

Protein_region 1958..2882

/note="Compiled from Protease CDS regions"

/Translation=GKREKGPAPGPNAHPIPPGYGAPTAEQPGTLQRATPGSTGLDLCAT

SGIILIPEDGPQVINTGVFGPPPSGTYFLILGRASATLSGITNFPSVVDADYEGEIKVL

AAATKGPLNISPGQRIAQALPVPFTKGLPHKGGRRGASNPGSSDAYWVQPLTKERPLLM

LSIDGRSFEGLLDSGADSTVLSQEHWPSSWPLQPSSTHLQGIGQSHNTLQSSKILTWSN

GEGNSGQIQPFVVPGLPVNLWGRDILAQMGAILCSPNEVVTKQMLQPGFIPGKGLGKSN

QGINSPIETTPKTNCHGLGYKEHFS*

/label=Protease

CDS 1958..2368

/label="Protease (Frame 2)"

Protein_domain 2036..2405

/label=dUTPase

Mutation 2369

/label="Frameshift (Insertion)"

CDS 2370..2882

/label="Protease (Frame 3)"

ORF 2461..2859

/Translation=MEGLLRAYLIRVQIPPFFPKNIGPPLGRYSPPPPTFKVLANLIIPY

RVARFSRGQMERETLVKSNPLWSQVYLLTCGGEIFWHKWGQYYAALTRWSPNKCYNLGS

YLVKGLENLIRELTHPLRPPQKQTVMV*

/label="Hypothetical Protein ORF 1"

Motif 2487..2495

/label=DxG

Slippage_site 2879

/label="Gag-Pro / Gag-Pro-Pol Slippage site"

Protein_region 2879..5430

/note="Compiled from Polymerase CDS regions"

/note="Contains a Stop mutation"

/Translation=IRVTDPPAPQADKIHWKSGDPVWVDQWPLPSYKISAALRLVQEQLG

AGRLGPSFSSNLKNGT*RLLQDLRKVNKTMQIMGPLQPGLPSPVAIPNKFFKIVIDLKD

CFFSIPLHPDDRPRFAFSIPITNHVGPTPRFQWRVLPQGMANSPTLCQKYVAQTIDPFR

VRYSTLYIVHYMDDILVAGPSPQLTHQVTQELIAALSKRGFVIAPEKVQTQPPLLFWGF

ELHANWVMSRKTQFRTSSLKTLNDFQKLLGDINWLRPYLKLTTGDLKPLFEILQGDPNP

ASLRSLTKEASQALSLVEEAIEQQFLGYFDPLQSLWFLVLPTAFIPTGLLWQGDPLFWV

HLSATPSKVLPSYPSLVCSLIRLGWLSAIRIFGKEPDIIVIPYDKAQRHWLEQFDPEWA

LTCTSYPGKFDNHYPANRLVQFLTTTAVVFPQVTKNQPLPQATLVFVDGSSNGRAAFDI

DGEISCFQTTYTSAQLVELQAVIAVFSKLPTTPFNLYSDSAYVVNAVRTLETVPLIKPS

SAASQLFATLQQLIVNRKHPFFIGHIRAHSGLPGPLSKGNNRVDQATRLVAISLTDPLA

EAKKAHTLHHLNANTLRHMFKITREQARDVVKGCKTCVTFLSEPHLGVNPRGLIPGELW

QMDVTHYPSFGTLKYLHVTIDTFSGYLFATAQTGEATKHVVSHLIACFAHLGVPKIIKT

DNGPGYTSSSLKQFCAQMQVKHVTGIPYNPQGQGIVERAHLTLKTMLTKLATSGGLLYP

QKGTQKTLLNHALFVLNFLSLDNAGRSAADRFWHPQTASNFATALWKDPLTAQWRGPDP

ILIWGKGHACIYDSAAQNARWLPDRLIKPFNRTQGGP*

/label=Polymerase

CDS 2879..4978

/label="Polymerase (Frame 2)"

Mutation 3065..3067

/label=Stop

Motif join(3191..3193,3407..3412)

/label=DDD

Motif join(4793..4795,4847..4849,4889..4891)

/label=DDD

CDS 4978..5430

/label="Polymerase (Frame 1)"

Mutation 4978

/label="Frameshift (Deletion)"

Protein_region 5486..7436

/note="Compiled from Envelope CDS regions"

/note="Contains Stop mutations"

/Translation=PSCIAHRIFNFTWTVVNEAGDIAYTTSTLASTTPWPTLTPDLCSLA

ARASPAWGLPDVFLPLPDKPPNNPSVAARWNGGCGSQSGRRFFKGPLGKNEPTGPALYV

CPGGHRSRTLNHQCGFRESYYCASWGCETTGDAYWHPVSTWDYIIVKKGWNNSQADEND

CGQKWCTPLLISFTDEGKRAPLVGWTRGHEWGLRLYVTGTDPGFTFKVKLILSTPNTNQ

KLF*AQIKPCLGRKAPKHPSGRILLSLPRALPIKPVCPPLGLPQVRP*RP*LMPLHSPL

TPRPEDCWMCFSPVPPFYEGIATVSPDMTYTNDSRQTRWVDSPFSESSPGLTLAQLSGI

GLCIHSTSLLLPPELLPICNTSHTPLTQHHFLVAPQGMYFACSFGITPSIVPQLLVENH

EYCVLVVLIPKVSIHPPEDLIPFYHSAPRVKREPVTAITLAVLLGLGATRAGTGIASII

TTNQQFHTLSLAIDKDIQNLQEGLDNLKESVVSLSEVVLQNRRGLDLLFLKEGGLCAAL

KEECCFYKDKTGLVQNSIEKIKKNLETRQKQREKDEAWYKSWASKNPLLSTLLPTILGP

LAGFLLLLSIGPWAVQKLTAFIKAQVNQLTKPAVAVHYHHLTTQDDDDVEQDPRHPRNL

NPSNTPLRLHRLL*

/label=Envelope

CDS 5486..7183

/label="Envelope (Frame 2)"

Mutation 6164..6166

/label=Stop

Mutation 6296..6298

/label=Stop

Mutation 6305..6307

/label=Stop

Mutation 7184

/label="Frameshift (Insertion)"

CDS 7185..7436

/label="Envelope (Frame 3)"

ORF 7698..8003

/note=KLTGGVGPLFPLSVKAHLSKKEREEMSGALSLHPKIRTTSCYREQTLLSVTLD

SHGTVKHPPFPVSLFPIPPPAEWEPATAGTAHLFLTSQVEITSSGGRQ*

/label="Hypothetical Protein ORF 2"

PPT 7755..7773

/note="Poly-purine tract"

LTR 7774..8103

/label="3' LTR (U3-R)"

ORIGIN

1 AAGTCATTGC TGTCCCATTT TTAACATGGG CCAATCTATC TCCAGAGAGG AGTCTTTTGT

61 CAAGGAGATC AAACGGTCTC TAAGGGAGAG AGGAGTAAGG GTTAAAAAAA AAGGATTTGG

121 TTAGTTTTTT CTGTTTTGTT GATGAAAAAT GTCCGTGGTT CATTCTTAGC GGCCCAGATA

181 TTCATCCCTT GAGTTGGCAA AAAGTAGGAA AAGATTTAAA TAGATTACTG GAAAAAGAGG

241 GACCAAATGC TGTGTCCGTT AGTTGTCTCA GTTATTGGGG ACTCATTCGA GACATCATTG

301 AAGGGGCCGA AACTGATAGT AATAAGAGAC AGCTGCTATC GGTAGCCACT GAAACGCTCA

361 AACAGCTCTC TCGCCCCCCA TCCGTTAAAG GTGGAGCTAC CTCTTCCCCC TGTCCTTCTG

421 TAGTCGTGGA CGTAGAGCCC TCGCCTCCGT TCGGAGACTC TCTTCCCCAA ATATACCCCA

481 TAATACCAAA ACCCGGCCCT GAACCCCTTG ATCCCGGAGA TGCAACTGTT CTGGAGGACG

541 AGGCCGCCAA ATATCATGAG CCTGTTTGGC CTTCATCTCA TACCTTTTTT GCTGCAGGGA

601 GGCCTGCCAC ACGCCCCCCT CCTTACAACC CCCTCCATAT CCCTCAGGCG GTTCTTGACC

661 CCTTTTTGGA GACAAAGAAA CAACTGCAAC AACAAATTCA AAGCCTCAAA AGCGTCTTTA

721 CTTTACAGAG TGAATTGGCT AATTTACAAT TAGAAGTCTC CGCTCTACAA AATTCTATTT

781 TTTCGGGGCC TCAATCTAAG CCCCCTAAGG GACCCCGGGC CAAACCTCTT GCTTTCCCTA

841 TTCTGACTCG ATCAGGTACC AGACCCTCAC AGGACCCGGA TACAACTCAG GAGGCGTCTA

901 CTGACGCCCA AGAGGCCCCT AGAGCCAGCA CACCTGATGA CCCAGCTTGT GAGGATGTGG

961 CACAATCTGA GGAAGAAGGG AAAAACCCAC ATAACGATTC TGAAAATGAG GGAGACTATC

1021 AGAACCTTTT GCCTACTGTA GAACGGATAA AATTTAAAAC TGTCAAAGAT TTACACAGTG

1081 CTATAAAGAG CTATGGCCTC ACAGCTCCCT TTACCTTGTC CATACTTGAA AGTTTGCCTG

1141 GCGATGGCTT TCTGTTGCCG GGAGAATGGA CAAGAGTGGC CCAATCTGTG CTGAGTAGGG

1201 GAGAATTTTT AACTTGGAAA GCAGAATTTT TTGACAGGGG GGAAACTCAG GCAATGCGCA

1261 ACCAGAAAAA TCCCCGGTCT CCCATGGCCA CCTGGACTGC TGATAAGATA TGTGGGAGGG

1321 GAGACTTTGC CTCTGAGCGT AAACAACGCA GTTTGTCTGC TGGGATCCTT AGTCAAACAG

1381 CGGCCGCTGC TATCGGGGCC TGGAGAGCCA TTCCTCAAAA AGGTTCGGTC ACCACGCCCT

1441 TGAGCAAAAT AGTTCAGGGG CCCCATGAAC CTTTCTCCCA GTTCGTTGCA CGTTTACAGG

1501 AAACTGCGGA GCGCGTCCTA GGGCCAGAAG ACGGGGAAGG GAAACTTGTA AAACAGCTTG

1561 CTTATGAAAA TGCTAATGCT GCCTGTAAAG CGGCTTTAAA GGGGCACCTC CGAAATCTCG

1621 ATATTCATGG GATGATTCGC TTGTGCAATG ATGTCGATTC CACCGCCCAT CAAATAAAGT

1681 TGGCCATTGG GGCTGTAATA CAACATGCTA ATAGTAACAC TCCTGTTCCC CAAAATACCC

1741 CCGTTGCCGC TCGGTGTTGC TTCCGCTGCG GACAACCTGG ACATGTTGCC CGACAATGCT

1801 CACTTCCCCG TACCGGGCAG ATCTTAGCTC CCCCTACAGT TCCTGTTCCC GTACCCCGCG

1861 CTCCGGGACT TTGCCCGCGT TGTAAGAAAG GGAAGCATTG GGCTAATGAG TGCCGCTCTA

1921 AAACTGATGT CGCGGGCAAC CCACTTCTTC CCTTGTCGGG AAACGGGAAA AGGGCCCAGC

1981 CCCGGGGCCC AACGCCCATC CAATTCCTCC CGGCTACGGG GCCCCGACAG CCGAACAACC

2041 AGGCACCCTC CAACGAGCCA CCCCAGGCAG CACAGGATTG GACCTCTGTG CCACCTCCGG

2101 TATAATATTA ATACCTGAGG ATGGGCCCCA AGTCATTAAT ACTGGGGTCT TCGGTCCTCC

2161 CCCTTCTGGA ACATACTTTC TAATTCTGGG ACGGGCCTCA GCCACTTTAT CTGGTATTAC

2221 CAACTTTCCC TCGGTAGTAG ATGCTGACTA TGAGGGGGAA ATAAAGGTCC TGGCGGCGGC

2281 CACGAAAGGG CCTTTAAATA TTTCTCCCGG CCAAAGAATT GCTCAAGCAC TCCCTGTGCC

2341 TTTTACAAAG GGGCTCCCTC ATAAGGGGGG GTCGCCGCGG TGCTTCAAAT CCCGGGTCCT

2401 CGGATGCATA CTGGGTCCAA CCCCTTACCA AGGAGCGACC CCTTCTCATG CTGTCTATAG

2461 ATGGAAGGTC TTTTGAGGGC TTACTTGATT CGGGTGCAGA TTCCACCGTT CTTTCCCAAG

2521 AACATTGGCC CTCCTCTTGG CCGTTACAGC CCTCCTCCAC CCACCTTCAA GGTATTGGCC

2581 AATCTCATAA TACCCTACAG AGTAGCAAGA TTCTCACGTG GTCAAATGGA GAGGGAAACT

2641 CTGGTCAAAT CCAACCCTTT GTGGTCCCAG GTCTACCTGT TAACTTGTGG GGGAGAGATA

2701 TTCTGGCACA AATGGGGGCA ATATTATGCA GCCCTAACGA GGTGGTCACC AAACAAATGT

2761 TACAACCTGG GTTCATACCT GGTAAAGGGC TTGGAAAATC TAATCAGGGA ATTAACTCAC

2821 CCATTGAGAC CACCCCAAAA ACAAACTGTC ATGGTCTAGG GTATAAGGAG CATTTTTCAT

2881 AAGGGTTACT GATCCTCCTG CACCCCAGGC GGATAAGATC CATTGGAAAT CAGGGGATCC

2941 TGTCTGGGTT GATCAGTGGC CCCTCCCCTC TTATAAGATT TCGGCCGCCC TCCGTTTAGT

3001 GCAGGAACAA TTAGGGGCTG GCCGTTTGGG GCCCTCTTTT TCCAGTAATC TCAAGAATGG

3061 TACCTGAAGG CTTTTACAGG ACCTAAGAAA AGTTAATAAA ACAATGCAAA TCATGGGGCC

3121 CTTACAGCCT GGGCTGCCTT CCCCCGTTGC TATTCCCAAC AAGTTCTTTA AAATTGTCAT

3181 TGATCTCAAG GATTGCTTTT TCTCTATACC TTTACACCCT GATGATCGCC CACGTTTTGC

3241 TTTTAGTATC CCCATTACCA ATCATGTTGG ACCAACACCC CGCTTTCAGT GGCGGGTTCT

3301 ACCCCAGGGG ATGGCAAACA GCCCTACTTT GTGTCAAAAA TACGTGGCCC AAACAATAGA

3361 CCCATTCAGG GTCCGTTACT CTACTCTTTA TATTGTACAT TACATGGATG ATATTCTTGT

3421 AGCAGGTCCC TCCCCACAAT TGACTCATCA GGTCACCCAG GAACTAATTG CTGCATTGAG

3481 CAAAAGAGGG TTTGTTATTG CCCCTGAAAA GGTACAAACC CAACCCCCCT TACTTTTTTG

3541 GGGGTTTGAA CTGCACGCCA ACTGGGTAAT GTCACGAAAA ACTCAATTCA GAACTTCATC

3601 TCTAAAAACA TTGAATGATT TTCAAAAACT GTTGGGAGAC ATTAACTGGC TTCGCCCTTA

3661 CTTAAAATTG ACCACAGGGG ATCTTAAACC GTTGTTTGAG ATTTTACAAG GAGACCCCAA

3721 TCCCGCTTCC CTACGTTCCC TAACGAAAGA AGCTTCACAG GCTCTCTCCC TGGTAGAGGA

3781 GGCTATCGAA CAACAGTTCT TGGGGTACTT TGATCCCCTA CAATCGCTGT GGTTTCTTGT

3841 TCTGCCCACC GCCTTCATAC CCACTGGCCT ACTCTGGCAG GGTGACCCCT TATTCTGGGT

3901 ACATCTCTCT GCTACCCCCT CAAAGGTCCT ACCATCTTAT CCTTCTTTGG TTTGCAGTTT

3961 AATTCGCCTA GGATGGCTCT CAGCAATAAG AATATTTGGC AAAGAACCTG ACATTATTGT

4021 GATCCCCTAT GATAAAGCCC AAAGACATTG GCTAGAACAG TTTGACCCGG AATGGGCTTT

4081 AACGTGCACC TCATATCCTG GGAAGTTTGA TAACCACTAC CCTGCCAATA GACTGGTTCA

4141 GTTCTTAACT ACCACTGCAG TGGTTTTCCC CCAAGTTACA AAGAACCAAC CATTGCCCCA

4201 GGCTACTCTA GTTTTTGTCG ATGGCTCCTC AAATGGTCGG GCCGCCTTCG ACATCGATGG

4261 GGAGATTAGC TGCTTTCAAA CAACTTATAC CTCTGCACAA TTGGTGGAAT TACAAGCTGT

4321 AATCGCTGTT TTTTCTAAGC TGCCTACTAC TCCCTTTAAC CTGTATTCTG ACAGCGCTTA

4381 CGTAGTAAAT GCTGTTCGTA CCTTAGAAAC AGTTCCCTTA ATCAAGCCAT CCTCCGCCGC

4441 TTCTCAACTT TTTGCCACCC TACAGCAGCT AATTGTGAAC AGAAAACATC CATTCTTTAT

4501 TGGACACATT CGCGCTCACT CAGGTCTACC CGGTCCTTTG TCGAAAGGCA ATAATCGAGT

4561 GGACCAGGCT ACTCGCCTAG TGGCCATCTC ACTTACAGAT CCCCTGGCTG AAGCAAAGAA

4621 AGCCCATACT CTACATCACC TCAATGCTAA TACCCTGAGA CACATGTTTA AAATTACTAG

4681 GGAACAGGCC AGAGACGTTG TAAAAGGTTG TAAAACTTGT GTCACCTTCC TTTCGGAACC

4741 ACACCTTGGG GTTAATCCCA GGGGCCTAAT TCCTGGAGAA CTGTGGCAAA TGGACGTAAC

4801 CCATTACCCT TCCTTTGGTA CGTTGAAATA CCTCCATGTA ACTATAGATA CCTTTAGTGG

4861 CTACCTGTTT GCTACTGCCC AAACAGGCGA AGCTACTAAA CATGTTGTGT CCCATCTTAT

4921 AGCCTGCTTT GCTCACCTGG GGGTACCTAA GATTATAAAA ACAGACAATG GCCCAGGTAT

4981 ACCAGTTCCT CTTTAAAACA ATTTTGTGCT CAAATGCAGG TCAAACATGT TACAGGTATT

5041 CCCTATAACC CACAGGGACA GGGTATAGTA GAGAGAGCTC ACCTCACCCT TAAGACCATG

5101 CTAACCAAAC TTGCCACATC AGGGGGATTA CTATATCCCC AAAAAGGAAC ACAAAAAACC

5161 CTTCTTAATC ATGCCCTGTT TGTTTTAAAT TTCTTGTCTC TTGACAATGC GGGCCGCTCC

5221 GCTGCAGACC GCTTCTGGCA CCCCCAGACC GCGTCAAACT TCGCTACAGC CCTGTGGAAG

5281 GACCCACTTA CCGCTCAATG GCGCGGGCCC GACCCAATAT TAATCTGGGG AAAAGGACAT

5341 GCTTGTATTT ATGATTCAGC CGCACAGAAC GCGCGCTGGT TACCCGACAG GCTAATTAAG

5401 CCATTTAACC GTACCCAGGG TGGCCCCTGA GAAGCTTTCT CTGCTTAATT TCAGAATGAT

5461 GTTCCTCCTG CTCATGTTTC TGCTGCCCTC CTGCATCGCT CATAGGATAT TCAACTTTAC

5521 TTGGACTGTC GTCAATGAAG CGGGTGACAT TGCTTATACT ACCTCAACCT TGGCAAGTAC

5581 AACCCCATGG CCTACACTCA CTCCAGACTT ATGCAGCCTA GCTGCCAGAG CATCACCAGC

5641 CTGGGGACTT CCAGATGTTT TTCTCCCCTT GCCTGACAAG CCGCCCAATA ACCCCTCTGT

5701 AGCAGCCCGC TGGAATGGGG GCTGTGGTTC ACAATCCGGG AGACGCTTCT TTAAGGGGCC

5761 CTTAGGGAAA AACGAACCTA CAGGCCCTGC TCTTTATGTT TGCCCAGGCG GGCACAGAAG

5821 CCGGACCCTT AATCACCAGT GTGGGTTTAG AGAATCTTAC TATTGTGCCT CCTGGGGGTG

5881 TGAAACCACG GGCGATGCCT ACTGGCACCC TGTCTCGACT TGGGATTATA TAATAGTCAA

5941 AAAGGGCTGG AATAATTCCC AGGCTGATGA GAACGATTGT GGACAAAAGT GGTGTACTCC

6001 CCTCCTCATT TCCTTTACTG ATGAAGGGAA AAGAGCGCCC CTCGTGGGAT GGACAAGGGG

6061 ACATGAATGG GGCCTAAGAC TTTATGTAAC TGGAACAGAT CCAGGGTTCA CATTCAAGGT

6121 AAAGTTAATT CTCTCCACAC CTAATACTAA CCAAAAATTG TTTTAGGCCC AAATAAAGCC

6181 CTGTCTCGGC CGCAAAGCCC CCAAACACCC CTCCGGGAGA ATACTCCTCT CCCTCCCCAG

6241 AGCACTGCCT ATCAAGCCCG TTTGCCCCCC GTTAGGCCTT CCACAGGTGA GGCCCTAAAG

6301 GCCCTAGCTA ATGCCACTGC ACAGTCCCTT AACACCTCGA CCTGAGGATT GTTGGATGTG

6361 TTTTTCCCCT GTTCCCCCTT TTTATGAAGG CATTGCAACT GTCTCGCCAG ACATGACCTA

6421 TACCAATGAT TCACGGCAGA CACGCTGGGT GGACTCTCCT TTTTCTGAGT CCTCTCCCGG

6481 GCTCACATTG GCCCAGTTGT CAGGAATTGG CCTCTGCATT CATAGTACCT CATTGCTTCT

6541 TCCCCCTGAA TTGCTACCTA TTTGCAATAC TTCCCATACA CCTCTAACTC AACACCATTT

6601 CCTTGTTGCT CCCCAAGGCA TGTATTTTGC TTGCTCTTTT GGAATAACCC CTTCTATTGT

6661 CCCTCAGTTA TTGGTAGAAA ATCATGAATA TTGTGTCCTA GTTGTGCTAA TACCTAAGGT

6721 CTCAATACAT CCTCCTGAAG ATTTGATTCC CTTTTATCAT AGCGCCCCGC GTGTCAAAAG

6781 GGAACCTGTA ACTGCCATCA CTCTGGCAGT CCTCTTGGGG TTGGGGGCCA CCAGAGCCGG

6841 GACCGGCATT GCCTCTATAA TCACAACAAA TCAACAATTC CATACTCTTA GTTTGGCTAT

6901 AGATAAGGAC ATTCAAAATC TGCAAGAAGG CCTTGATAAC CTTAAAGAAT CTGTTGTTTC

6961 ACTTTCTGAG GTAGTTCTTC AAAATCGCCG CGGTCTTGAC CTTCTATTTC TTAAAGAAGG

7021 CGGTCTGTGC GCTGCCCTTA AAGAAGAATG TTGTTTTTAC AAAGATAAAA CTGGGTTAGT

7081 CCAAAACAGT ATTGAAAAAA TAAAGAAAAA TCTGGAGACC CGGCAAAAAC AAAGAGAAAA

7141 GGACGAAGCC TGGTATAAAA GTTGGGCCTC TAAAAACCCC CTAGCTGTCC ACCCTACTTC

7201 CTACTATTCT CGGACCTCTA GCAGGGTTCC TCCTTTTACT GTCGATTGGC CCCTGGGCAG

7261 TGCAAAAACT AACTGCTTTT ATTAAAGCAC AGGTTAATCA ACTAACTAAA CCAGCTGTTG

7321 CCGTCCACTA CCACCATTTG ACAACTCAGG ATGATGACGA TGTGGAACAA GATCCCCGGC

7381 ACCCTAGAAA TCTTAACCCC TCTAATACAC CCCTGCGCCT GCATCGACTT CTTTAAGGCC

7441 TAGCTCCCCC CCACACATGG GGCGGCATGA AACTAGAGAC ATGCCTACCT CACCCTTGGA

7501 TGATGGTCTA GAGGTATGGG CACCGAGCAG AGTGACGGGC AAAGCACCGC AAGGAAGGGC

7561 CCTTCTGTGC CTTCTCTAGT CCTTCCTGAG AACATGCCTG ACTTGCATAG AGGTTGGTAT

7621 CATAGTAATA TTACACAAAC CGGCTCTGCC TCTCCTCCCC AAAAGATACC AAGAGCCATA

7681 GCTGGTGGGT TCATTAAAAG CTCACGGGAG GAGTCGGGCC TCTGTTCCCT CTCTCGGTCA

7741 AAGCCCACCT TTCTAAAAAA GAAAGGGAGG AGATGTCGGG AGCCTTAAGC CTGCACCCGA

7801 AGATAAGAAC CACATCCTGT TACCGAGAAC AAACTCTGCT ATCTGTGACT TTGGACAGCC

7861 ACGGCACTGT GAAGCACCCG CCATTTCCCG TGTCTTTGTT CCCCATTCCG CCACCTGCAG

7921 AGTGGGAGCC TGCAACTGCT GGCACAGCCC ATCTGTTTCT GACTAGCCAA GTAGAAATCA

7981 CCTCATCTGG TGGTCGCCAA TAAGCTTGTA ACGAATACTC TCTGATCAGT CCCTCCCCTG

8041 CCCTCTCCAC TTGAGTTTAT AAATATAACC GCTCGAAAAT AAAATTTTGA GGCTTGATCA

8101 GAA

//

**Pteropus alecto Retroviral Envelope protein 01 (PaEnv-01)**

LOCUS PaEnv-01_Locus31mer_326_Transcript_2525_Confidence_0390 4299 bp DNA linear UNA

FEATURES Location/Qualifiers

Protein_region 1728..3678

/note="Compiled from Env CDS regions"

/Translation=PSCIAHRIFNFTWTVVNEAGDIAYTTSTLASTTPWPTLTPDLCSLA

ARASPAWGLPDVFLPLPDKPPNNPSVAARWNGGCGSQSGRRFFKGPLGKNEPTGPALYV

CPGGHRSRTLNHQCGFRESYYCASWGCETTGDAYWHPVSTWDYIIVKKGWNNSQADEND

CGQKWCTPLLISFTDEGKRAPLVGWTRGHEWGLRLYVTGTDPGFTFKVKLILSTPNTEP

KIVLGPNKALSRPQSPQTPLRENTPLPPQSTAYQARLPPVRPSTGEALKALANATAQSL

NTSTEDCWMCFSPVPPFYEGIATVSPDMTYTNDSRQTRWVDSPFSESSPGLTLAQLSGI

GLCIHSTSLLLPPELLPICNTSHTPLTQHHFLVAPQGMYFACSFGITPSIVPQLLVENH

EYCVLVVLIPKVSIHPPEDLIPFYHSAPRVKREPVTAITLAVLLGLGATRAGTGIASII

TTNQQFHTLSLAIDKDIQNLQEGLDNLKESVVSLSEVVLQNRRGLDLLFLKEGGLCAAL

KEECCFYKDKTGLVQNSIEKIKKNLETRQKQREKDEAWYKSWASKTP*LSTLLPTILGP

LAGFLLLLSIGPWAVQKLTAFIKAQVNQLTKPAVAVHYHHLTTQDDDDVEQDPRHPRNL

NPSNTPLRLHRLL*

/label=Env

CDS 1728..2585

/label=Env

Mutation 2586

/label="Frameshift (Insertion)"

CDS 2587..3678

/label=Env

ORIGIN

1 AGGCTCTCTC CCTGGTAGAG GAGGCTATCG AACAACAGTT CTTGGGGTAC TTTGATCCCC

61 TACAATCGCT GTGGTTTCTT GTTCTGCCCA CCGCCTTCAT ACCCACTGGC CTACTCTGGC

121 AGGGTGACCC CTTATTCTGG GTACATCTCT CTGCTACCCC CTCAAAGGTC CTACCATCTT

181 ATCCTTCTTT GGTTTGCAGT TTAATTCGCC TAGGATGGCT CTCAGCAATA AGAATATTTG

241 GCAAAGAACC TGACATTATT GTGATCCCCT ATGATAAAGC CCAAAGACAT TGGCTAGAAC

301 AGTTTGACCC GGAATGGGCT TTAACGTGCA CCTCATATCC TGGGAAGTTT GATAACCACT

361 ACCCTGCCAA TAGACTGGTT CAGTTCTTAA CTACCACTGC AGTGGTTTTC CCCCAAGTTA

421 CAAAGAACCA ACCATTGCCC CAGGCTACTC TAGTTTTTGT CGATGGCTCC TCAAATGGTC

481 GGGCCGCCTT CGACATCGAT GGGGAGATTA GCTGCTTTCA AACAACTTAT ACCTCTGCAC

541 AATTGGTGGA ATTACAAGCT GTAATCGCTG TTTTTTCTAA GCTGCCTACT ACTCCCTTTA

601 ACCTGTATTC TGACAGCGCT TACGTAGTAA ATGCTGTTCG TACCTTAGAA ACAGTTCCCT

661 TAATCAAGCC ATCCTCCGCC GCTTCTCAAC TTTTTGCCAC CCTACAGCAG CTAATTGTGA

721 ACAGAAAACA TCCATTCTTT ATTGGACACA TTCGCGCTCA CTCAGGTCTA CCCGGTCCTT

781 TGTCGAAAGG CAATAATCGA GTGGACCAGG CTACTCGCCT AGTGGCCATC TCACTTACAG

841 ATCCCCTGGC TGAAGCAAAG AAAGCCCATA CTCTACATCA CCTCAATGCT AATACCCTGA

901 GACACATGTT TAAAATTACT AGGGAACAGG CCAGAGACGT TGTAAAAGGT TGTAAAACTT

961 GTGTCACCTT CCTTTCGGAA CCACACCTTG GGGTTAATCC CAGGGGCCTA ATTCCTGGAG

1021 AACTGTGGCA AATGGACGTA ACCCATTACC CTTCCTTTGG TACGTTGAAA TACCTCCATG

1081 TAACTATAGA TACCTTTAGT GGCTACCTGT TTGCTACTGC CCAAACAGGC GAAGCTACTA

1141 AACATGTTGT GTCCCATCTT ATAGCCTGCT TTGCTCACCT GGGGGTACCT AAGATTATAA

1201 AAACAGACAA TGGCCCAGGT ATACCAGTTC CTCTTTAAAA CAATTTTGTG CTCAAATGCA

1261 GGTCAAACAT GTTACAGGTA TTCCCTATAA CCCACAGGGA CAGGGTATAG TAGAGAGAGC

1321 TCACCTCACC CTTAAGACCA TGCTAACCAA ACTTGCCACA TCAGGGGGAT TACTATATCC

1381 CCAAAAAGGA ACACAAAAAA CCCTTCTTAA TCATGCCCTG TTTGTTTTAA ATTTCTTGTC

1441 TCTTGACAAT GCGGGCCGCT CCGCTGCAGA CCGCTTCTGG CACCCCCAGA CCGCGTCAAA

1501 CTTCGCTACA GCCCTGTGGA AGGACCCACT TACCGCTCAA TGGCGCGGGC CCGACCCAAT

1561 ATTAATCTGG GGAAAAGGAC ATGCTTGTAT TTATGATTCA GCCGCACAGA ACGCGCGCTG

1621 GTTACCCGAC AGGCTAATTA AGCCATTTAA CCGTACCCAG GGTGGCCCCT GAGAAGTTTT

1681 CTCTGCTTAC TTTCAGAATG ATGTTCCTCC TGCTCATGTT TCTGCTGCCC TCCTGCATCG

1741 CTCATAGGAT ATTCAACTTT ACTTGGACTG TCGTCAATGA AGCGGGTGAC ATTGCTTATA

1801 CTACCTCAAC CTTGGCAAGT ACAACCCCAT GGCCTACACT CACTCCAGAC TTATGCAGCC

1861 TAGCTGCCAG AGCATCACCA GCCTGGGGAC TTCCAGATGT TTTTCTCCCC TTGCCTGACA

1921 AGCCGCCCAA TAACCCCTCT GTAGCAGCCC GCTGGAATGG GGGCTGTGGT TCACAATCCG

1981 GGAGACGCTT CTTTAAGGGG CCCTTAGGGA AAAACGAACC TACAGGCCCT GCTCTTTATG

2041 TTTGCCCAGG CGGGCACAGA AGCCGGACCC TTAATCACCA GTGTGGGTTT AGAGAATCAT

2101 ACTATTGTGC CTCCTGGGGG TGTGAAACCA CGGGCGATGC CTACTGGCAC CCTGTCTCTA

2161 CTTGGGATTA TATAATAGTC AAAAAGGGCT GGAATAATTC CCAGGCTGAT GAGAACGATT

2221 GTGGACAAAA GTGGTGTACT CCCCTCCTCA TTTCCTTTAC TGATGAAGGG AAAAGAGCGC

2281 CCCTCGTGGG ATGGACAAGG GGACATGAAT GGGGCCTAAG ACTTTATGTA ACTGGAACAG

2341 ATCCAGGGTT CACATTCAAG GTAAAGTTAA TTCTCTCCAC ACCTAATACT GAACCAAAAA

2401 TTGTTTTAGG CCCAAATAAA GCCCTGTCTC GGCCGCAAAG CCCCCAAACA CCCCTCCGGG

2461 AGAATACTCC TCTCCCTCCC CAGAGCACTG CCTATCAAGC CCGTTTGCCC CCCGTTAGGC

2521 CTTCCACAGG TGAGGCCCTA AAGGCCCTAG CTAATGCCAC TGCACAATCC CTTAACACCT

2581 CGACCTGAGG ATTGTTGGAT GTGTTTTTCC CCTGTTCCCC CTTTTTATGA AGGCATTGCA

2641 ACTGTCTCGC CAGACATGAC CTATACCAAT GATTCACGGC AGACACGCTG GGTGGACTCT

2701 CCTTTTTCTG AGTCCTCTCC CGGGCTCACA TTGGCCCAGT TGTCAGGAAT TGGCCTCTGC

2761 ATTCATAGTA CCTCATTGCT TCTTCCCCCT GAATTGCTAC CTATTTGCAA TACTTCCCAT

2821 ACACCTCTAA CTCAACACCA TTTCCTTGTT GCTCCCCAAG GCATGTATTT TGCTTGCTCT

2881 TTTGGAATAA CCCCTTCTAT TGTCCCTCAG TTATTGGTAG AAAATCATGA ATATTGTGTC

2941 CTAGTTGTGC TAATACCTAA GGTCTCAATA CATCCTCCTG AAGATTTGAT TCCCTTTTAT

3001 CATAGCGCCC CGCGTGTCAA AAGGGAACCT GTAACTGCCA TCACTCTGGC AGTCCTCTTG

3061 GGGTTGGGGG CCACCAGAGC CGGGACCGGC ATTGCCTCTA TAATCACAAC AAATCAACAA

3121 TTCCATACTC TTAGTTTGGC TATAGATAAG GACATTCAAA ATCTGCAAGA AGGCCTTGAT

3181 AACCTTAAAG AATCTGTTGT TTCACTTTCT GAGGTAGTTC TTCAAAATCG CCGCGGTCTT

3241 GACCTTCTAT TTCTTAAAGA AGGCGGTCTG TGCGCTGCCC TTAAAGAAGA ATGTTGTTTT

3301 TACAAAGATA AAACTGGGTT AGTCCAAAAC AGTATTGAAA AAATAAAGAA AAATCTGGAG

3361 ACCCGGCAAA AACAAAGAGA AAAGGACGAA GCCTGGTATA AAAGTTGGGC CTCTAAAACC

3421 CCCTAGCTGT CCACCCTACT TCCTACTATT CTCGGACCTC TAGCAGGGTT CCTCCTTTTA

3481 CTGTCGATTG GCCCCTGGGC AGTGCAAAAA CTAACTGCTT TTATTAAAGC ACAGGTTAAT

3541 CAACTAACTA AACCAGCTGT TGCCGTCCAC TACCACCACT TGACAACTCA GGATGATGAC

3601 GATGTGGAAC AAGATCCCCG GCACCCTAGA AATCTTAACC CCTCTAATAC ACCCCTGCGC

3661 CTGCATCGAC TTCTTTAAGG CCTAGCTCCC CCCCACACAT GGGGCGGCAT GAAACTAGAG

3721 ACATGCCTAC CTCACCCTTG GATGAGGGTC TAGAGGTATG GGCACCGAGC AGAGTGACGG

3781 GCAAAGCACC GCAAGGAAGG GCCCTTCTGT GCCTTCTCTA GTCCTTCCTG AGAACATGCC

3841 TGACTTGCAT AGAGGTTGGT ATCATAGTAA TATTACACAA ACTGGCTCTG CCTCTCCTCC

3901 CCAAAAGATA CCAAGAGCCA TAGCTGGTGG GTTCATTAAA AGCTCACGGG AGGAGTCGGG

3961 CCTCTGTTCC CTCTCTCGGT CAAAGCCCAC CTTTCTAAAA AAGAAAGGGA GGAGATGTCG

4021 GGAGCCTTAA GCCTGCACCC GAAGATAAGA ACCACATCCT GTTATCGAGA ACAGACTCTG

4081 CTAGCTGTGA CTTTGGACAG CCACGGCACT GTGAAGCACC CGCCATTTCC CGTGTCTTTG

4141 TTCCCCATTC CGCCACCTGC AGAGTGGGAG CCTGCAACTG CTGGCACAGC CCATCTGTTT

4201 CTGACTAGCC AAGTAGAAAT CACCTCATCT GGTGGACACC AATGAGCTTG TAACGAATAC

4261 TCTCTGATCA GTCCCGCAGA TCGGAAGAGC GGTTCAGCA

//

**Rhinolophus ferrumequinum Retroviral Envelope protein 01 (RfEnv-01)**

LOCUS RfEnv-01 1149 bp DNA linear UNA

FEATURES Location/Qualifiers

Protein_region 1..1149

/Translation=KKFAEIQKTLNPKFTYHPLAHPKKPGHVDIDPQTFDILSSTHKLLL

SVNSSYATDCWLCLLQGTPLPLAIPYPFVTSTTNNSCNIALPFLVQPLGFNNTPCVLCP

TQNDTTEVNLGSLTFTICSSFINVSSPMCAPNGSVYICGNNYAYTYLPQNWTGVCTLGS

LLPDVSIIPGDEPVPIPTFEHIAGRTKRAVHFIPLLAGLGITTALATRSAGIGHSLVQY

HKLSGQLISDVQALSETIQDLQDQVDSLAEVVLQNRRGLDLLTAEKGGICLALGEKCCF

YANKSGIVRDRVKKLQEDLEKRRRDLLSNPLWTGFNGLLPYLLPLLGPILGCFILLSLG

PILLNKLTRFLRQQIEALQAKPIQVHYTRLEMQERGDPYLQ*

/label=Env

ORIGIN

1 AAAAAATTTG CTGAAATACA AAAGACCCTT AACCCTAAAT TTACCTATCA CCCTTTGGCC

61 CACCCTAAAA AACCGGGTCA CGTGGACATT GATCCTCAGA CTTTTGACAT TCTTAGTTCT

121 ACCCACAAGT TATTGCTTTC TGTTAATTCA TCCTACGCCA CAGACTGCTG GCTGTGTTTA

181 CTACAAGGCA CCCCTTTACC ATTAGCTATA CCCTATCCCT TTGTCACCTC TACTACCAAT

241 AATTCATGCA ACATAGCTCT CCCTTTTTTA GTCCAACCCC TTGGCTTTAA CAATACCCCG

301 TGCGTCCTCT GTCCCACTCA AAACGATACT ACAGAGGTTA ATTTAGGAAG TCTCACCTTT

361 ACAATTTGCT CCTCCTTCAT TAATGTATCC TCTCCTATGT GTGCACCCAA TGGATCGGTA

421 TATATTTGTG GAAATAATTA TGCCTACACC TATTTACCAC AAAACTGGAC AGGAGTTTGT

481 ACCCTAGGCT CCCTCCTCCC AGATGTATCC ATCATTCCAG GAGATGAGCC AGTCCCTATC

541 CCGACTTTCG AACATATTGC AGGACGCACT AAACGTGCAG TCCATTTTAT TCCCTTATTA

601 GCGGGTCTAG GCATCACCAC CGCACTTGCC ACCAGGTCCG CGGGGATAGG ACATTCCCTA

661 GTACAATACC ATAAATTATC TGGACAACTC ATATCAGATG TCCAGGCACT CTCAGAAACT

721 ATCCAAGATC TTCAGGATCA GGTTGATTCC CTAGCAGAAG TTGTCCTCCA AAACAGGAGG

781 GGATTAGATT TACTTACTGC AGAAAAAGGG GGCATCTGTC TGGCCCTCGG AGAAAAATGC

841 TGTTTTTATG CTAACAAATC TGGAATTGTT CGTGACAGAG TCAAAAAATT ACAAGAAGAC

901 CTTGAAAAAA GAAGAAGAGA CCTCCTTTCC AACCCTCTCT GGACCGGATT CAATGGACTT

961 TTACCCTACT TACTACCCCT GCTTGGCCCC ATACTCGGGT GCTTTATCCT ACTATCACTG

1021 GGACCCATCC TCCTCAATAA ACTCACGCGC TTTCTCAGAC AACAAATAGA GGCCTTGCAG

1081 GCCAAGCCCA TACAGGTCCA TTACACCCGA CTGGAGATGC AAGAGCGAGG AGATCCCTAT

1141 CTCCAATAA

//

**Pteropus alecto Retroviral Polymerase protein 01 (PaPol-01)**

LOCUS PaPol-01_Locus21_19710_Transcript_24_Confidence_0700 1808 bp DNA linear UNA

FEATURES Location/Qualifiers

Protein_region 2..1669

/note="Contains Stop mutations"

/Translation=VESAISAQQVQYIDYTQE*YGYVLATKHTPTGVLWQHGPLRWIHLP

VSPSKVLNPYHEAVASLIQLLREESRKYFGKEPHIIVVPFTEQQLNWLFQNSDSWNIAF

AYYTGKIDNHYSGHKLLQFANMQSFIFPRVIKTTPIQNALNIFTDGSSNGKAVYAVDNQ

NPIVIQTSPASAQIIELRAVAAVFQIFAQRLLNLYTDSQYIAKALIILETVPYIATHNS

EVHSLLAQIQLAIHNRKEFCYVTHIRAHTNLPGPLSKANALVDAATHLCLLTSNVEQA*

QSHAIHHQNSSYLRKQFHLTREAARQIIKSCPTCPQFFHVPHYGINPRDLVPNQIWQMD

VTHIPEFGKLKYVHVTVDTFSGFIFASALTGEATKHVIDHCLRCFAAIGCPQILKTDNG

SGYTSAAFKTFCSQLHIEHKTGIPYNPQGQGIVERAHGSLKTQLEKIKKGELYPYSPQN

YLNHALFVLNFLNLDAKGHSAAERLWHPTTTSNYATVRWKDPLTGQWNGPDPVLIWGRG

HVCVFPQGADGARWLPERLVRHAENEHRDYSSDGNTD*

/label=Pol

ORIGIN

1 GGTAGAATCC GCTATATCTG CCCAGCAGGT ACAATATATT GATTATACTC AAGAGTGATA

61 TGGATATGTT CTCGCTACAA AACATACACC TACTGGTGTT TTGTGGCAGC ATGGACCCTT

121 ACGATGGATC CACCTCCCTG TGTCCCCTTC TAAAGTTCTC AATCCATATC ATGAAGCAGT

181 GGCTTCTCTC ATACAGCTGT TAAGAGAAGA GTCAAGGAAG TACTTTGGCA AAGAGCCACA

241 TATCATAGTA GTACCATTTA CTGAACAGCA ATTAAATTGG TTATTTCAAA ATTCTGACTC

301 ATGGAACATA GCCTTTGCAT ATTATACAGG AAAAATTGAC AATCATTATT CTGGTCACAA

361 ACTTTTACAG TTTGCTAATA TGCAGTCATT TATCTTTCCT CGTGTAATTA AGACCACCCC

421 GATACAGAAT GCTCTTAATA TATTTACTGA TGGTTCCTCT AATGGAAAAG CCGTGTATGC

481 TGTTGACAAC CAAAATCCTA TTGTCATACA AACATCCCCT GCATCTGCTC AGATTATAGA

541 ATTGCGGGCA GTTGCTGCAG TTTTTCAAAT ATTTGCTCAA CGACTCCTTA ACCTATATAC

601 TGATAGTCAA TACATTGCTA AAGCTTTAAT TATATTGGAG ACTGTACCTT ATATTGCTAC

661 CCACAATTCT GAGGTGCATT CATTATTGGC GCAAATTCAA TTGGCTATAC ACAACAGAAA

721 AGAATTCTGC TATGTGACTC ATATTCGAGC TCATACAAAT CTCCCTGGTC CTTTGTCTAA

781 GGCTAATGCA CTTGTTGATG CAGCCACCCA CCTATGTCTG CTTACTAGTA ATGTTGAACA

841 AGCATGACAA TCTCATGCTA TACATCATCA AAATAGCTCT TATCTAAGAA AGCAATTTCA

901 TCTCACCAGA GAAGCAGCAA GACAAATTAT AAAAAGTTGT CCCACTTGCC CTCAGTTTTT

961 TCATGTTCCT CATTATGGTA TTAATCCCCG TGATTTGGTA CCGAATCAAA TTTGGCAAAT

1021 GGATGTAACT CACATTCCTG AATTTGGTAA ATTAAAATAT GTACATGTTA CTGTAGATAC

1081 ATTTTCTGGA TTCATTTTCG CTTCTGCTTT GACTGGTGAA GCCACTAAAC ATGTTATTGA

1141 TCACTGCTTA CGCTGCTTTG CAGCTATTGG ATGTCCTCAA ATTCTTAAGA CAGACAATGG

1201 CTCTGGTTAT ACTAGTGCCG CTTTTAAAAC ATTTTGCTCT CAGTTACATA TTGAACATAA

1261 AACTGGTATT CCTTACAACC CACAAGGTCA AGGCATTGTG GAACGTGCCC ATGGCTCTCT

1321 AAAGACTCAA CTTGAGAAAA TAAAAAAGGG GGAGTTATAC CCCTATTCTC CACAAAATTA

1381 TTTAAATCAT GCCTTATTCG TTTTAAATTT TTTAAATTTG GATGCCAAAG GGCACTCTGC

1441 AGCTGAGCGC TTATGGCATC CCACTACTAC TTCTAATTAT GCCACTGTAA GATGGAAAGA

1501 TCCGTTAACT GGACAATGGA ATGGACCAGA TCCTGTACTA ATATGGGGGC GAGGGCATGT

1561 TTGTGTTTTT CCGCAGGGAG CTGATGGAGC ACGCTGGTTG CCTGAACGAC TAGTGCGGCA

1621 TGCCGAAAAT GAACATCGAG ATTATTCTTC TGATGGGAAT ACTGATTAAT CCAACAGTTT

1681 ATGCTGCAGT GCATTGGGCT TACATCCCGG ATCCTCCGGT ACTACATCCA ACTGTATGGT

1741 CTGGGCCACA GATCCAGGTT TTTTCTGCCA TTAGCCATGG AACAGCAATT TCTATTCAAT

1801 TCTACTTC

//
